# The origin and evolution of a two-component system of paralogous genes encoding the centromeric histone CENH3 in cereals

**DOI:** 10.1186/s12870-021-03264-3

**Published:** 2021-11-18

**Authors:** Evgeny A. Elisafenko, Elena V. Evtushenko, Alexander V. Vershinin

**Affiliations:** 1 Institute of Cytology and Genetics, SB RAS, Novosibirsk, 630090 Russia; 2 Institute of Molecular and Cellular Biology, SB RAS, Novosibirsk, 630090 Russia; 3 Novosibirsk State University, Novosibirsk, 630090 Russia

**Keywords:** Centromere, CENH3, Gene duplication, Molecular evolution, Invasion of transposable elements, *Poaceae*

## Abstract

**Background:**

The cereal family Poaceae is one of the largest and most diverse angiosperm families. The central component of centromere specification and function is the centromere-specific histone H3 (CENH3). Some cereal species (maize, rice) have one copy of the gene encoding this protein, while some (wheat, barley, rye) have two. We applied a homology-based approach to sequenced cereal genomes, in order to finally trace the mutual evolution of the structure of the *CENH3* genes and the nearby regions in various tribes.

**Results:**

We have established that the syntenic group or the *CENH3* locus with the *CENH3* gene and the boundaries defined by the *CDPK2* and *bZIP* genes first appeared around 50 Mya in a common ancestor of the subfamilies Bambusoideae, Oryzoideae and Pooideae. This locus came to Pooideae with one copy of *CENH3* in the most ancient tribes Nardeae and Meliceae. The *βCENH3* gene as a part of the locus appeared in the tribes Stipeae and Brachypodieae around 35–40 Mya. The duplication was accompanied by changes in the exon-intron structure. Purifying selection acts mostly on αCENH3s, while βCENH3s form more heterogeneous structures, in which clade-specific amino acid motifs are present. In barley species, the *βCENH3* gene assumed an inverted orientation relative to *αCENH3* and the *CDPK2* gene was substituted with *LHCB-l*. As the evolution and domestication of plant species went on, the locus was growing in size due to an increasing distance between *αCENH3* and *βCENH3* because of a massive insertion of the main LTR-containing retrotransposon superfamilies, *gypsy* and *copia*, without any evolutionary preference on either of them. A comparison of the molecular structure of the locus in the A, B and D subgenomes of the hexaploid wheat *T. aestivum* showed that invasion by mobile elements and concomitant rearrangements took place in an independent way even in evolutionarily close species.

**Conclusions:**

The *CENH3* duplication in cereals was accompanied by changes in the exon-intron structure of the *βCENH3* paralog. The observed general tendency towards the expansion of the *CENH3* locus reveals an amazing diversity of ways in which different species implement the scenario described in this paper.

**Supplementary Information:**

The online version contains supplementary material available at 10.1186/s12870-021-03264-3.

## Background

Histone H3 is one of the four histone proteins that compose nucleosome cores. Canonical histone H3 has a globular C-terminal domain (HFD, the histone-fold domain), which harbors four helix motifs (αN, α1, α2, α3) and the loop1 and loop2 regions. A putative recognition site for histone chaperones involved in nucleosome assembly partially overlaps with the α2 helix [1]. The N-terminal domain (NTT, N-terminal tail) is considered to function in the formation of higher-order chromatin, and deletion of the H3 NTT affects histone-DNA interactions and substantially decreases nucleosome stability [2]. The specific role of histone H3 is that inclusion of one of its variants (the one designated as CENP-A in animals and as CENH3 (centromere-specific histone H3) in plants) in the nucleosomes defines the location of centromeres on chromosomes [3] and is essential for centromere and kinetochore formation in most organisms [4]. Within the HFD, loop1 and α2 helix are necessary for targeting centromeric histone H3 to the centromere and the region formed by the two is referred to as the CATD [5]. The crystal structure of the nucleosome revealed that CENP-A structurally differs from conventional histone H3 at the N-terminal region in the nucleosome and this specific structure may cause the unwrapping of DNA at the entry/exit regions in the nucleosome [6]. Overall, the DNA wraps less tightly in centromeric than in conventional nucleosomes and these differences suggest that they are distinct from canonical H3 nucleosomes [7, 8]. The histone H3 family comprises at least three canonic H3 variants in most eukaryotes and these members, H3.1, H3.2, and H3.3, share more than 95% identity, while CENH3s are highly divergent in sequence compared to canonical histone H3, particularly in N-terminal tail [9].

DNA regions with homology to histone *H3* genes have been found in the genomes of primitive organisms, such as unicellular algae, and *H3* gene families have expanded and diversified across plant evolution to reach 14–15 genes in land plants, such as maize and rice [10]. Arabidopsis contains 15 histone *H3* genes, including five *H3.1* genes, one *H3.1-*like gene, three *H3.3* genes, five *H3.3-*like genes and one *CENH3* gene [11]. Introns have been found only in the *Н3.3* genes, three in switchgrass (*Rapicum virgatum* L.) [12], but not in canonical histone *H3* (*H3.1*), suggesting that the histone variant *H3.1* is likely to have evolved from *H3.3* [13].

The first plants to have CENH3 identified in were *Arabidopsis thaliana* [14], maize (*Zea mays*) [15] and rice (*Oryza sativa*) [16]. Each of these species has one copy of *CENH3*, although WGD (whole-genome duplication) has been suggested in all three species. A comparison of the structure of the CENH3 and the canonical histone H3 proteins showed that they share a conserved histone fold domain at their carboxyl ends [9]. The N-terminal tails of CENH3s, however, differ from those of canonical histone H3s and vary in both length and sequence among different species. As more and more species with monocentric chromosomes (that is, those that have only one centromere) have become known, it comes to the researchers’ attention that although most of these species have one copy of *CENH3*, some have two or more, which synthesize different variants of proteins. *CENH3* duplication in diploid plants was first detected as having occurred in *Arabidopsis* species, *A. halleri* and *A. lyrata*, after their ancestor split from that of *A. thaliana* [17]. Both copies are functional, both have identical exon-intron structures (9 exons), but one of them has a 16-bp deletion relative to the original gene in *A. thaliana*. Subsequently, *CENH3* duplications were shown for diploid plants in different taxa: *Brassica* [18], *Pisum* and *Lathyrus* [19], *Mimulus* [20], *Vigna* [21]. Both copies produce functional CENH3 proteins, which fully colocalize with each other.

The discovery of *CENH3* duplications complicated the understanding of the evolution of the *CENH3* genes and raised the question about the mechanisms and causes of gains, losses and selective constraints*.* An intriguing aspect of *CENH3* evolution is that the duplications that have occurred in *A. halleri*, *A. lyrata*, and *Pisum* and *Lathyrus* species are preserved and expressed, while the closely related *A. thaliana* or *Vicia* and *Lens* species, which are in the same tribe *Fabeae* as are *Pisum* and *Lathyrus*, express only a single copy of this central component of centromere specification and function. This motivated us to extend the analysis of *CENH3* duplications to other plant taxa. To start with, we chose the cereal family Poaceae. This is one of the largest and most diverse angiosperm families, consisting of more than 12,000 species and more than 700 genera [22]. Most of the economically important species that have long been used in breeding, with numerous crosses involving closely related and distant species, are in this family. The phylogenetic classifications of this family, which were in the past largely based on morphology and anatomy, have in the last three decades been revised based on molecular evidence. Another advantage of Poaceae as an object for studying the evolutionary history of the two-component system of paralogous genes encoding the centromere-specific histone CENH3 is that this family includes not only rice (*Oryza sativa*) and maize (*Zea mays*), which have one copy of *CENH3*, but also Triticeae species (barley, wheat and rye, to name a few), which have two copies of this gene [23–25].

An increased number of cereal genomes sequenced in recent years and advances in their assembly allowed us to use a homology-based approach and to finally trace back the mutual evolution of the structure of the *αCENH3* and *βCENH3* genes and structure of the nearby regions in various tribes in the family Poaceae. We established that around 50 Mya the common ancestor of the subfamilies Bambusoideae, Oryzoideae and Pooideae developed the *CENH3* locus composed of the syntenic genes *CDPK2, CENH3* and *bZIP*. The duplication event took place within the *CENH3* locus in Stipeae and Brachypodieae species around 35–40 Mya. It was accompanied by substantial changes in the exon-intron structure of the daughter gene, *βCENH3*. The *αCENH3* and *βCENH3* genes were subject to different degrees of selection constraints. Purifying selection acts mostly on *αCENH3*s. A lower pressure from purifying selection allowed profound changes to occur in nucleotide/amino acid sequences, especially in the NTT domain of βCENH3s, in which clade-specific amino acid motifs are present. The other process is that the *CENH3* locus is expanding as the distance between *αCENH3* and *βCENH3* increases due to a massive insertion of elements of the LTR-containing retrotransposon superfamilies, *gypsy* and *copia*. Changes in the molecular structure of the *CENH3* locus and the observed general tendency towards its expansion reveal an amazing diversity of ways in which different species implement this scenario.

## Results

### Selection of cereal species and identification of *CENH3* paralogs in their genomes

**Fig. 1:**
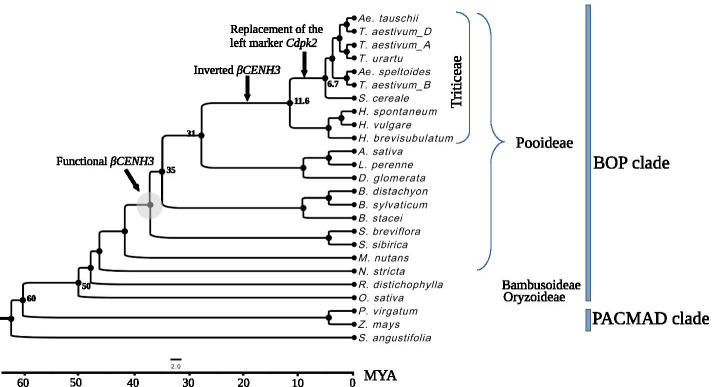
The phylogenetic tree inferred for the study species according to the classification system of Soreng et al. [22]. Under this system, ВОР and РАСМАD are two main clades: ВОР comprises the subfamilies Bambusoideae, Oryzoideae and Pooideae, and РАСМАD comprises the subfamilies Panicoideae, Aristidoideae, Chloridoideae, Micrairoideae, Arundinoideae and Danthonioideae. The subfamilial and tribal attribution of the study species and some of their details are given in Table 1. On the scale: the ВОР/РАСМАD split time (60 Mya) and various lineage split times according to Chalupska et al. [27]

Our choice of species was based on the worldwide phylogenetic classification of the family Poaceae, which was created using known molecular markers and morphological characteristics [22]. This classification put together the latest molecular advances with data from a large number of previous morphological studies and placed 771 genera of this family in 51 tribes and 12 subfamilies [22]. The well-documented presence of one CENH3 variant in maize and rice [15, 16] and of two variants in Triticeae [23–25] suggests that *CENH3* duplication occurred after the subfamily Pooideae, which includes Triticeae, split from a common ancestor with the subfamily Oryzoideae, which includes rice (Fig. 1). According to Soreng et al. [22], the subfamily between these two is Bambusoideae. Based on this, we identified a selection of promising candidate Poaceae species that could be helpful in tracing the evolutionary history of *CENH3* duplication. The list and some details of these species are shown in Table 1, where species belonging to different tribes and subfamilies are arranged in the order of divergefnce according to the classification system we are using [22]. *Streptochaeta angustifolia* in the subfamily Anomochlooideae was used as an outgroup. Another important factor for choosing candidate species was the availability of high-quality well-annotated genomes. Triticeae species are the most represented in the selection, first, because they have *CENH3* paralogs and, secondly, because their genomes are well studied. The study considers all *CENH3* genes and CENH3 proteins in the species that are considered to be the progenitors of three ancestral diploid genomes (ABD) of hexaploid wheat (*Triticum aestivum*)—*Triticum urartu* (the ancestor of the A genome), *Aegilops speltoides* (a likely ancestor of the B genome) and *Aegilops tauschii* (the ancestor of the D genome)—and the ABD subgenomes of *T. aestivum*. Additionally, the study included two copies of *αCENH3* present in the N and K genomes of tetraploid switchgrass (*Panicum virgatum*) and two copies of *βCENH3* found in oats (*Avena sativa*). Oats is a hexaploid species, and two copies of *βCENH3* (*βCENH3*_1 and β*CENH3*_2) are localized in different subgenomes (***Avena sativa*** **– OT3098 v2, PepsiCo,** https://wheat.pw.usda.gov/jb?data=/ggds/oat-ot3098v2-pepsico**)**. To identify genomic copies of *CENH3*, we used the fully sequenced genomes or extended contigs of promising candidate species and transcriptomes available in the NCBI and Phytozome databases (Table 1 and Additional file 1: Table S1). To make use of the partially assembled genomes of *Stipa sibirica* and *Lolium perenne*, we extracted available reads from the SRA at NCBI (https://www.ncbi.nlm.nih.gov) and assembled them into contigs using Minia [26].

**Table 1:** The study species and their details

Species	Tribe*	Subfamily*	Genome size (1C), Mbp, pg	Ploidy, chromosome number
*Streptochaeta angustifolia*	Streptochaeteae	Anomochlooideae	N/A	2n = 2x = 22**
*Panicum virgatum*	Paniceae	Panicoideae	1370^1^; 1.4	2n = 4x = 36
*Zea mays*	Andropogoneae	Panocoideae	3280; 2.7	2n = 2x = 20
*Oryza sativa*	Oryzeae	Oryzoideae	466; 0.5	2n = 2x = 24
*Raddia distichophylla*	Olyreae	Bambusoideae	600^2^; N/A	2n = 2x =22***
*Nardus stricta*	Nardeae	Pooideae	2050^1^; 2.1	2n = 2x =26
*Melica nutans*	Meliceae	Pooideae	2450^1^; 2.5	2n = 2x = 18
*Stipa sibirica* *Stipa breviflora*	Stipeae	Pooideae	~ 980^1^; ~ 1.0	2n = 2x = 22 or 24
*Brachypodium distachyon* *Brachypodium sylvaticum* *Brachypodium stacei*	Brachypodieae	Pooideae	272; 0.32 529; 0.44 234; 0.28	2n = 2x = 10 2n = 2x = 18 2n = 2x = 20
*Avena sativa*	Poeae	Pooideae	12520^1^; 12.8	2n = 6x = 42
*Dactylis glomerata*	Poeae	Pooideae	4420; 3.3	2n = 4x = 28**
*Lolium perenne*	Poeae	Pooideae	2770; 2.7	2n = 2x = 14
*Hordeum brevisubulatum*	Triticeae	Pooideae	8800^1^; 9.0	2n = 4x = 28
*Hordeum vulgare,* ssp. *spontaneum*	Triticeae	Poideae	5180^1^; 5.3	2n = 2x = 14
*Hordeum vulgare*	Triticeae	Pooideae	5180; 5.3	2n = 2x = 14
*Secale cereale*	Triticeae	Pooideae	8460; 8.7	2n = 2x = 14
*Triticum urartu*	Triticeae	Pooideae	5.75; 5.9	2n = 2x = 14
*Aegilops speltoides*	Triticeae	Pooideae	5680; 5.8	2n = 2x = 14
*Aegilops tauschii*	Triticeae	Pooideae	5050; 5.2	2n = 2x = 14
*Triticum aestivum*	Triticeae	Pooideae	17,280; 17.7	2n = 6x = 42

*Note*: Genome sizes are taken mainly from Leitch et al. [27]. Plant DNA C-values Database (release 7.1*)*. https://cvalues.science.kew.org/

^1^ Due to the absence of these values in Databases, they are calculated using the formula: 1 pg = 978 Mbp [28]

^2^ Li et al. [29]

* tribal and subfamilial attribution to the classification system of Soreng et al. [22]; ** Pohl, Davidse [30]; *** Hunziker et al. [31]

Two considerations are important for further reading. One relates to the analysis of the genome of cultivated barley (*Hordeum vulgare* L.). According to the most updated version of its genome, GCA_902498975.1, *βCENH3* is on chromosome 1Н 72 kb away from *αCENH3*. Unlike the *βCENH3*s in other Triticeae species, the one in question contains a 18-bp deletion in the NTT and a stop codon in exon 3 in the 3′-end of the HFD and, therefore, the protein it synthesizes should be as short as 126 amino acids (aa), which is 28 aa shorter than its ortholog in wild barley *Hordeum brevisubulatum* (Additional file 2: Fig. S1). The structure of *βCENH3* in *Hordeum vulgare* subsp. *spontaneum* (hereinafter *H. spontaneum*), which is the closest relative and progenitor of cultivated barley [33], is similar to that of its ortholog in *H. vulgare*. Because it has been shown that the C-terminal tail of CENH3 is responsible for interactions between CENH3 and histone H4 in nucleosomes and the last five amino acids in the C-terminus are necessary for CENH3 deposition [34], it is unlikely that a protein molecule so shortened will be functional. One more copy of *βCENH3* was found on barley chromosome 6H, with no alpha paralog around. This copy has a 17-aa deletion in the N-terminus, the deletion being longer than that in 1H, and a normal-size C-terminus (Additional file 2: Fig. S1). Because antibodies against βCENH3 had previously been obtained and located in barley centromeres by immunostaining [35], it seems logical to assume that this *βCENH3* sequence may be capable of producing a functional βCENH3 molecule. In further analysis, we will use both copies of *βCENH3* from chromosomes 1H and 6H, and the *CENH3* paralogs from *H*. *brevisubulatum*.

The other consideration relates to difficulties in identifying the functional *βCENH3* transcripts in *Brachypodium distachyon*. The genome of this species is small and compact, is often considered a model for the large and often polyploid genomes of Triticeae species and is well studied [36, 37]. It was therefore important to find out when exactly *CENH3* duplication occurred in Pooideae: before or after the speciation of *Brachypodium*. It had been previously argued that only one *CENH3* paralog is present in *B. distachyon* [38, 39], 1776 bp in size, which is substantially larger than *αCENH3* in the other Triticeae species. We extracted the genomic sequences of *CENH3* with adjacent regions for *B. distachyon* from GenBank, and for two other species, *Brachypodium stacei* and *Brachypodium sylvaticum*, from the Phytozome database. The sequence of the 1776-bp transcript, LOC100830307, is annotated in the genome of *B. distachyon* (NC_0161323) (Additional file 2: Fig. S2). A comparison with known sequences from other cereal species revealed that this transcript has homologies with both *αCENH3* and *βCENH3*. However, its exon 2 has a stop codon in it. Similar degenerate copies or truncated fragments of *CENH3* have been found on two soybean chromosomes (*Glycine max*) [19].

We wanted to find out whether or not the stop codon in exon 2 of the *βCENH3* gene in *B. distachyon* from GenBank was a sequencing error. To do so, we obtained cDNA from RNA, line B21 and resequenced the transcripts from locus LOC100830307. All versions of the transcripts and the primers to the 5′- and 3′-ends of *βCENH3* and *αCENH3* are schematically shown in Additional file 2: Fig. S3. The vast majority of *βCENH3* transcripts (92%) had a stop codon at the same position in them as in genome sequence NC_0161323. It is therefore obvious that the annotated *B. distachyon* genome contains both *CENH3* paralogs.

An aberrant beta form (Bstacei_316_v1.0, Phytozome) with stop codons was also found in *B. stacei* (Additional file 2: Fig. S2). Additionally, this species has the full-length beta form Brast08G079400.1.p, but its HFD sequence is substantially different from its counterparts in other species. In *B. sylvaticum*, no transcript of either *CENH3* paralog has a stop codon; here Brasy8G088800.1.p is given as an example (Additional file 2: Fig. S2). Thus, among the three *Brachypodium* species studied, only *B. sylvaticum* has been found to have *βCENH3* that is capable of producing a functional protein molecule. *βCENH3* of this species was used in further analysis.

### CENH3 protein sequence alignments and phylogenetic trees

To identify the genomic copies of *CENH3*, we ran sequence similarity searches in the fully sequenced genomes of promising candidate species and transcriptomes from NCBI and Phytozome databases (Additional file 1: Table S1), and the *CENH3*-containing genomic loci assembled by us for *Stipa sibirica* and *Lolium perenne* (Additional files 3 and 4). From the species for which only transcriptomes are known (*Stipa breviflora, Melica nutans* and *Nardus stricta*), only cDNA and protein sequences were collected and analyzed (Additional files 5, 6, 7, 8). The nucleotide or amino acid sequence of *CENH3* in a particular species or in its closest relatives was used as a search query. It was found that the transcriptomes of *N. stricta* (the tribe Nardeae) and *M. nutans* (the tribe Meliceae) have only *αCENH3*, while Stipeae species and the species in the other Pooideae tribes (Brachypodieae, Poeae and Triticeae) have two, *αCENH3* and *βCENH3*. Thus, according to the classification system of Soreng et al. [22], two functional *CENH3* paralogs appeared in Pooideae either before Stipeae and Brachypodieae split or independently thereafter (Fig. 1).

**Fig. 2:**
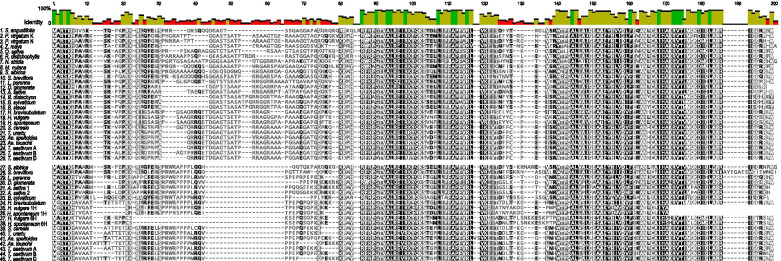
A multiple alignment of the amino acid sequences of CENH3 proteins using the MUSCLE program [40] For convenience, the alpha and beta forms are grouped into two separate blocks: 1–26 (αCENH3) and 27–45 (βCENH3). Amino acid residues identical in all species are shown as white letters on the black background.

**Fig. 3:**
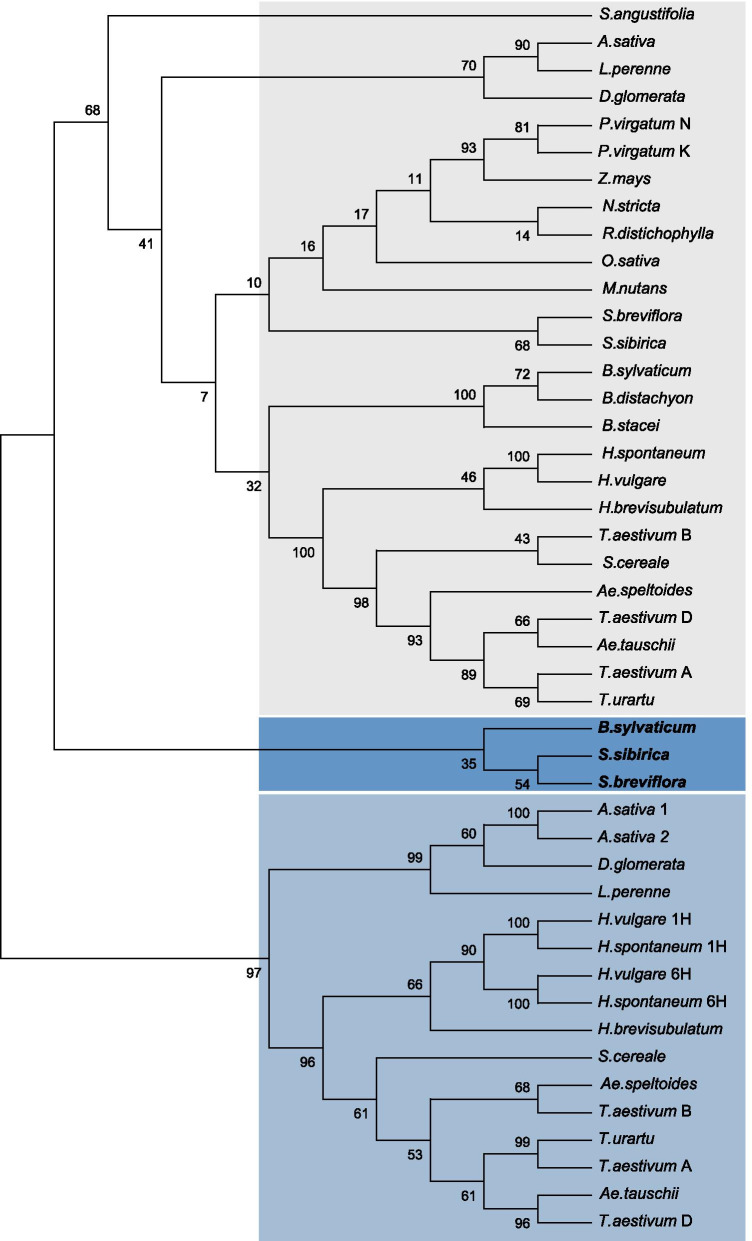
The phylogenetic tree inferred from a comparison of the deduced CENH3 proteins using the ML method, the JTT matrix-based model (Jones et al. 1992) and bootstrapping (1000 replicates). Initial tree(s) for the heuristic search were obtained automatically by applying Neighbor-Join and BioNJ programs to a matrix of pairwise distances estimated using a JTT model, and then selecting the topology with superior log likelihood value. The tree with the highest log likelihood (− 4953.06) is shown. A discrete Gamma distribution was used to model evolutionary rate differences among sites (5 categories (+*G*, parameter = 1.0416)). The tree is drawn to scale, with branch lengths measured in the number of substitutions per site. This analysis involved 45 amino acid sequences. There were a total of 200 positions in the final dataset. Evolutionary analyses were conducted in MEGA X [43]. Bootstrap values were calculated from at least 1000 replications. Amino acid sequences were aligned using MUSCLE with default settings. On the branches: bootstrap values. The αCENH3-only clade is in a gray-filled shape, the βCENH3-only clade is in a light-blue-filled shape; the βCENH3 clade comprised of *Stipa* spp. and *Brachypodium sylvaticum* is in a dark-blue-filled shape

Multiple alignments of the deduced amino acid sequences of the αCENH3 and βCENH3 proteins found in the species listed in Tables 1, 45 samples in total, were obtained using the MUSCLE algorithm [40] (Fig. 2) and Bali-Phy [41] (Additional file 2: Fig. S4). Phylogenetic trees were inferred using the Maximum Likelihood (ML) method and JTT matrix-based model [42]. Evolutionary analyses were conducted in MEGA X [43]. The tree constructed using MUSCLE with the highest log likelihood (− 4953.02) is shown in Fig. 3. The trees constructed using these alignment methods show (1) a good discrimination between the species that have only one form of CENH3 (alpha) and the species that have two forms of CENH3 (alpha and beta) and (2) a good discrimination between the αCENH3-only clade (in a gray-filled shape) and the βCENH3-only clade (in a light-blue-filled shape). The ‘core’ Pooideae tribes Triticeae and Poeae [44] form distinct clusters within both clades, consistent with their positions in the classification system, which is based on the largest number of different characters [22]. Of special interest is the position of CENH3 proteins in *S. sibirica*, *S. breviflora* and *B. sylvaticum*, because these were the first species reported to have both CENH3 paralogs at once. On the ML tree, (Fig. 3) αCENH3s of *Stipa* species cluster is well separated from the cluster with αCENH3s of *Brachypodium* species. Looking at this topology, we can readily suggest that the probable scenario is that of an independent duplication of *CENH3* in *Stipa* and *Brachypodium* species. The cluster with the βCENH3 copies of the *Stipa* species and *B. sylvaticum* (in a dark-blue-filled shape) lies separately, closer to the alpha-only clade. This intermediate position of the beta forms in these species suggests the presence of specific features—motifs—in their structure, characteristic of the alpha form of the protein.

In all species, the N-domains of both CENH3 paralogs have a more heterogeneous structure than do the C-termini. That becomes especially evident when amino acid alignments are generated with Bali-Phy (Additional file 2: Fig. S4). Specific motifs (most in the NTT) characteristic of certain groups of species are contained in the rectangular boxes with different border colors. In *S. sibirica* and *S. breviflora*, the NTTs of βCENH3 have 10 and 9 amino acid residues (red borders), respectively, that are present only in the alpha forms and are not present in the beta forms in other species except the *Stipa* species. The HFDs of βCENH3 in the *Stipa* species also have species-specific motifs in the loop1 region. The βCENH3 of the Pooideae species with both paralogs have specific NTT motifs characteristic of Triticeae and Poeae (green and pink borders, respectively). Barley stands out from all the other Triticeae species – this can be seen from the topology of the phylogenetic trees: it forms a cluster well separated from other species (Fig. 3) and has a species-specific amino acid motif in the loop1 region of the βCENH3 HFD (Additional file 2: Fig. S4). Noteworthy, both paralogs of wild barley *H*. *brevisubulatum* are closer to other Triticeae species than are those of *H. vulgare*.

### Lineage-specific evolution

Gene duplication generally implies the reproduction of the sequence of a gene in its copy. It seems logical to assume that differences in the structure of copies will characterize the rates of their evolutionary changes and the degree of their divergence since the duplication event. One of the quantitative characteristics of these differences may be the degree of homology of the amino acid and/or nucleotide sequences. A comparison of the amino acid sequences of αCENH3 and βCENH3 aligned using FASTA showed that their similarity ranges from 65 to 70% in most species (Table 2). This is substantially lower than the 75% similarity between the two paralogous proteins, CenH3–1 and CenH3–2, in Fabeae species [19] and 91% similarity between the proteins CENH3.1 and CENH3.2 in *Vigna unguiculata* [21]. However, *S. sibirica* and *S. breviflora* have markedly higher similarity values relative to other species, 77.5 and 75.2%, respectively. The difference in similarity between *Stipa* and other species is stronger in the NTTs than in the HFDs (Table 2). Thus, either the rate of divergence of the CENH3 paralogs was lower in *Stipa* species than in most Pooideae species that have two CENH3 paralogs or duplications in *Stipa* are more recent events.

**Table 2:** The similarity of the deduced amino acid sequences of αCENH3 and βCENH3

Species	Tribe	Similarity of protein copies, %	Similarity of NTTs, %	Similarity of HFDs, %
*Stipa sibirica* *Stipa breviflora*	Stipeae	77.5 75.2	64.9 58.4	87.5 89.8
*Brachypodium sylvaticum*	Brachypodieae	70.4	52.9	83.5
*Lolium perenne*	Poeae	69.0	42.7	90.2
*Dactylis glomerata*	Poeae	71.7	50.7	87.8
*Avena sativa* β1* β2	Poeae	65.7 66.1	42.7 55.6	86.7 86.7
*Hordeum brevisubulatum*	Triticeae	74.7	53.4	91.4
*Hordeum spontaneum* 1H** 6H	Triticeae	62.9 65.5	45.6 42.7	81.9 82.8
*Hordeum vulgare* 1H** 6H	Triticeae	61.4 64.9	54.0 42.7	81.9 82.8
*Secale cereale*	Triticeae	70.4	44.9	93.5
*Triticum urartu*	Triticeae	69.6	45.8	90.3
*Aegilops speltoides*	Triticeae	67.8	45.0	88.4
*Aegilops tauschii*	Triticeae	70.4	43.4	91.4
*Triticum aestivum* A*** B D	Triticeae	69.6 71.3 70.4	43.4 44.3 43.4	90.3 92.5 91.4

*Note*: Separate HFD regions are singled out according to Karimi-Ashtiyani et al. [45]

* data for two copies of *βCENH3* in *Avena sativa*

** data for *βCENH3* on chromosomes 1Н and 6Н

** data for the A, B and D genomes of *Triticum aestivum*

The type of selective pressure acting on the *CENH3* genes during their diversification was identified based on the observed ratios of nonsynonymous (Ka) to synonymous (Ks) nucleotide substitution rates (Ka/Ks = ω) separately for each ortholog. All-to-all pair-wise comparisons of full-length *CENH3* coding sequences using the Ka-Ks calculator [46] estimated ω to be less than 1 for each of the orthologs in all pairs of species being compared (Additional file 1: Tables S2, S3), indicating purifying (stabilizing) selection as the major force shaping the diversity of these genes. For *αCENH3*, this ratio was in all cases substantially lower, with most values falling in the range of 0.2–0.4 (Additional file 1: Table S2), indicating a considerable strong purifying selective pressure. For *βCENH3*, the ω values were higher, in the range of 0.4–0.7, and for two cases of *βCENH3* located on chromosome 6H of *H. vulgare* they exceeded 1 (highlighted in bold, Additional file 1: Table S3). Although the observed excess fails to reach significance, it is sufficient for concluding that *βCENH3* paralogs are under relaxed selective pressure.

The dominating impact of purifying selection does not exclude the possibility that diversifying positive selection acts on specific branches of phylogenetic trees. We extended our analysis so far as to identify specific branches that have sites evolving with ῳ > 1 and used adaptive branch site-random effects likelihood (aBSREL) [47]. This branch-site model simultaneously implements ῳ variation across branches and sites. aBSREL requires no prior knowledge about which lineages are more likely to have experienced episodic diversifying selection. Significance was assessed by the likelihood ratio test (LRT) at a threshold of Р ≤ 0.05. The most statistically significant (at *P* < 0.05) evidence of episodic diversifying selection is found on the branch leading to the cluster (node 11, *P* = 0.0073) containing βCENH3 in *S. sibirica*, *S. breviflora* and *B. sylvaticum* (Additional file 2: Fig. S5b). No diversifying selection was noted in the cluster with the Triticeae species, with the exception of the branch with βCENH3 on chromosome 6H of *H. vulgare* (*P* = 0.0447). The percentage of the sites driven by positive selection in each case is indicated in brackets in Additional file 2: Fig. S5.

In the tree constructed for αCENH3 with BSREL (Additional file 2: Fig. S5a), *B. sylvaticum* is clustered with the other *Brachypodium* species and the greatest influence of diversifying selection is exerted on the branches leading to the cluster of *Stipa* species (node 34, *P* = 0.0448) and further to *S. sibirica* (*P* = 0.0000). Thus, a good match can be observed on the branches, in which some of the sites within the alpha and beta forms of the centromere-specific histone undergo positive diversifying selection.

### The exon-intron structure of *αCENH3* and *βCENH3*

**Fig. 4:**
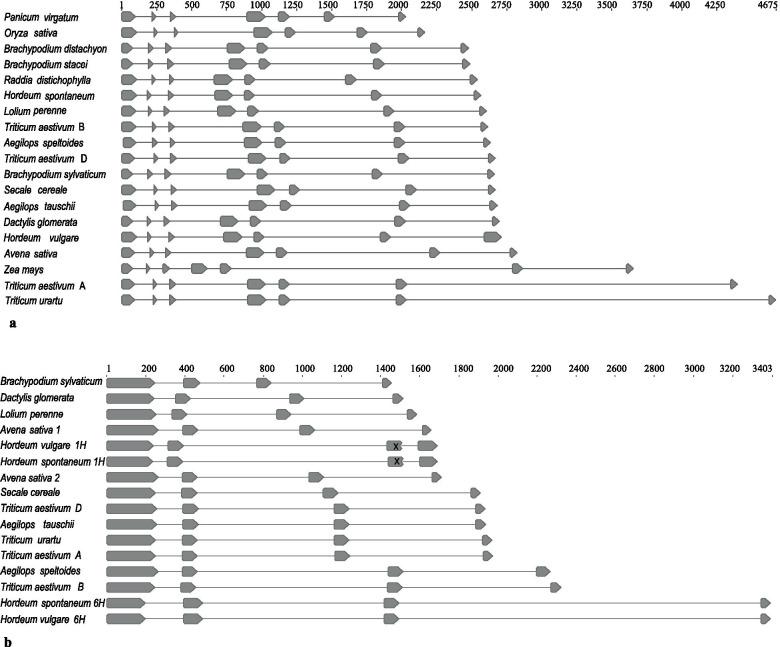
The exon-intron structure of the *CENH3* genes in the study cereals. **a:** *αCENH3*; **b:** *βCENH3*. The upper scale shows the dimensions in bp. The positions of stop codons in the third exon of *βCENH3* localized on the 1H chromosomes of barley are shown by crosses. Exons appear as finger-post arrows

Transcriptome libraries were used to identify *CENH3* transcripts (Additional files 5-8) and to infer the exon-intron structure of *αCENH3* and *βCENH3* in those cereal species in Table 1 for which both genomic and transcriptome libraries were available. A comparison of alpha orthologs showed that the *αCENH3* structure is conserved, no matter whether a given species has both paralogs or only the alpha. Each *αCENH3* gene has seven exons encoding proteins 159–172 amino acids in length and separated by six introns (Fig. 4a). The total size of the *αCENH3* genes varies considerably across species, from 2050 to 4679 bp, due to differences in intron sequences. The introns at the 3′-end of *αCENH3* are much longer than those at its 5′-end, and introns 6 of *αCENH3* in *T. urartu* and the A genome of *T. aestivum* are the longest, 2588 and 2312 bp, respectively.

The structural features of the *αCENH3* paralogs—a conserved number of exons and introns in different species and longer introns at the 3′-ends of the genes—are also typical of all *βCENH3* paralogs. However, the latter contain fewer exons and introns: four exons and three introns. An exon-intron structure similarly different from the *αCENH3* genes is found in Brachypodium, one of the first tribes to have had these two paralogs. It is logical to assume that these changes have nothing to do with the post-duplication evolutionary history, but accompanied the duplication event.

**Fig. 5:**
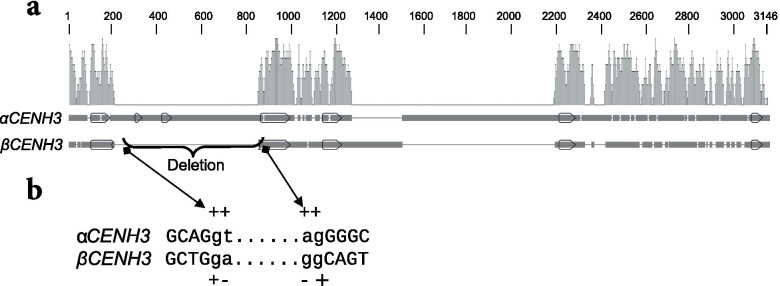
A comparison of α*CENH3* and β*CENH3* sequences in *B. sylvaticum*. **a** Homologous regions in *αCENH3* and *βCENH3*. Exons appear as finger-post arrows. Thin/thick black straight lines: the presence/absence of sequences in the alignment. In the *Identity* row: homology between the genes in this region, from 0 to 100%. **b** Aberrant splice sites in *βCENH3* after deletion of two exons together with adjacent introns. The splice sites of exon 1 and exon 4 of *αCENH3* (gt…ag). The exon sequence is in uppercase; the sequence of the putative splice site of the introns is in lowercase; +/−, matches/mismatches with the canonical GT|AG

As follows from the results presented, two functional paralogs of *CENH3* first appeared in Stipeae and Brachypodium. We carried out a detailed comparative analysis of the genomic copies in *B. sylvaticum*. The sequences of the *αCENH3* and *βCENH3* genes together with the adjacent 5′- and 3′- regions at the *CENH3* locus were analyzed and compared using MUSCLE (Fig. 5). As can be seen, the shaping of the *βCENH3* structure was accompanied by an extended deletion including the complete sequences of exon 2 and exon 3 and a part of the 3′-end of exon 1 and of the 5′-end of exon 4, together with the corresponding introns (Fig. 5a). Interestingly, the 3′-boundary of the resulting extended exon—exon 1—in *βCENH3* matched the 3′-boundary of exon 4 in *αCENH3*. Figure 5b shows aberrations in the splice sites of *βCENH3* after deletion of two exons. The exon-intron structure described is characteristic of *βCENH3* in all subsequent species on our evolutionary scale (Fig. 4B). The size of exon 1 of *βCENH3* in different species is 223–264 bp, which notably exceeds the size of exon 1 of *αCENH3*, 76–127 bp (Fig. 4). Thus, *βCENH3* has undergone such profound changes in nucleotide sequences (and, consequently, amino acid sequences) and in exon-intron structure that it is so different from *αCENH3* now.

### The structure of the *CENH3* locus in evolution

**Fig. 6:**
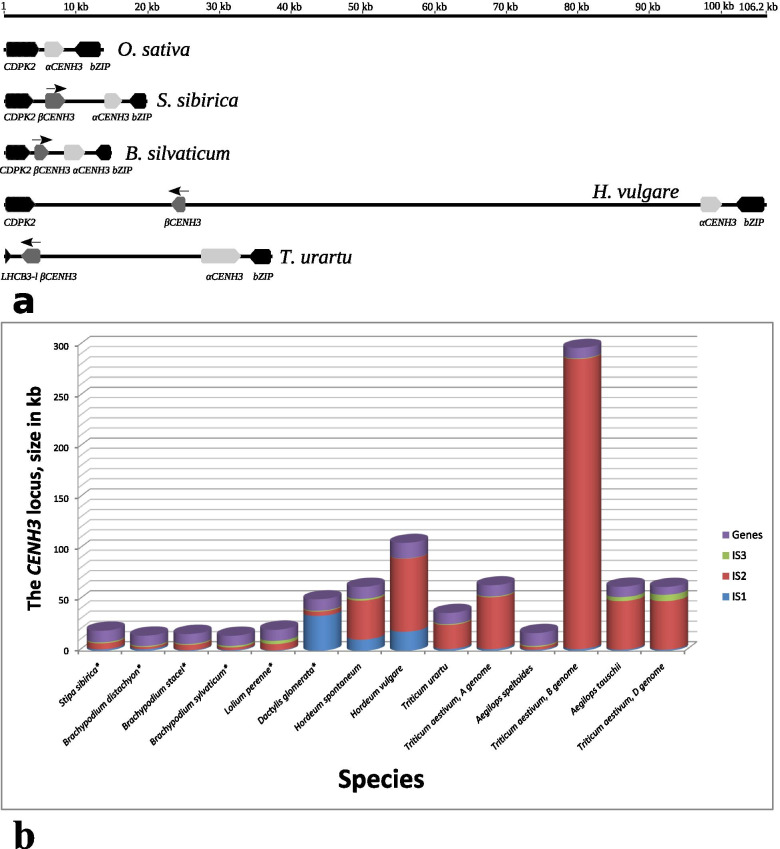
The structure and evolutionary changes of the *CENH3* locus. **a** Syntenic genes comprising the *CENH3* locus. The species with important events, such as the formation of a syntenic group (rice), the emergence of the beta paralog (*Stipa*, *Brachypodium*), the inversion of the beta paralog (barley) and replacement of the 5′ marker gene (*T. urartu*) in their evolutionary history are given as examples. **b** The evolutionary changes in the size of the genes and intergenic spacers (ISs) comprising the *CENH3* locus in Pooideae. Species not belonging to the tribe Triticeae, are marked with asterisks

We examined the genomic environment of the *αCENH3* and *βCENH3* paralogs and found that upstream of *βCENH3* in most of the studied genomes are the *LHCB-l* gene, which encodes chlorophyll a-b binding protein 3C, in Triticeae species and the *CDPK2* gene, which encodes calcium-dependent protein kinase 2, in the other species. Downstream of *αCENH3* in all species is a *bZIP* transcription factor gene. Thus, these genes, together with *CENH3*, can be considered a syntenic group or the *CENH3* locus, with *CDPK2* and *bZIP* as its left-hand and right-hand boundary, respectively. We assumed that a detailed analysis of the molecular structure of the *CENH3* locus would facilitate our understanding of its evolutionary history. Figure 6a shows a schematic of this locus in some cereal species. In maize, which, according to the classification system of Soreng et al. [22], is in the PACMAD clade (Fig. 1), no *CENH3* locus with the left-hand or the right-hand marker gene has been found; the genes closest to *αCENH3* are those for leucine-rich repeat (LRR) family protein and non-specific lipid transfer protein GPI-anchored 2.

This classification system we used assigns the Triticeae species, in which two paralogs of the centromeric histone gene were first found (barley, rye and wheat), to the BOP clade, which includes the subfamilies Bambusoideae, Oryzoideae and Pooideae (Fig. 1). According to the established order of divergence from a common ancestor of the BOP and PACMAD clades, the first species in our set is rice (*O. sativa*). In the rice genome, the above-described collinearity of genes is present and the *CENH3* locus is short, 15.7 kb, of which 2.18 kb is contributed by one copy of *CENH3* (Fig. 6a). *R. distichophylla*, a species in the subfamily Bambusoideae, has the *CENH3* locus with a single copy of the gene, as does rice.

Two functional paralogs of this gene, *βCENH3* and *αCENH3*, appear in Stipeae and Brachypodieae, both paralogs being in the same 5′ → 3′ orientation. The size of the locus in *B. distachyon* is very close to that in rice (15 kb), and *S. sibirica* has it as large as nearly 20 kb. The distances between *βCENH3* and *αCENH3* (intergenic spacer, IS2) range from 2.16 kb in *B. distachyon* to 5.9 kb *S. sibirica* (Fig. 6b). In *S. sibirica*, most of IS2, 3.27 kb, is given to the non-LTR retrotransposon LINE1-34_SBi. Additionally, the locus reveals a short 215-bp fragment of the transposable element *Harbinger* and a few relatively short (a few dozens of base pairs) tracks of simple repeats. IS2 between the *CENH3* paralogs in *Brachypodium* species has much the same composition. Curiously, the intergenic spacers in all these species do not have the smallest fragments of LTR-containing retrotransposons, the most common class of transposable elements in plants.

Poeae species are observed to have an increased size of the entire *CENH3* locus (Fig. 6b). Not only the already mentioned classes of DNA sequences—LINE, simple repeats and short fragments of various DNA transposon families—contribute considerably to the increase in these sizes, but also extended fragments of LTR retrotransposons. Two main superfamilies of LTR retrotransposons, *copia* and *gypsy*, are nearly equal contributors in extended IS2. *D. glomerata* is found to have families that are also present in the IS2 of the Triticeae species: *Eugene*, *Usier, Inga* (all belong to *copia*), *Sabrina*, *Sabina*, *Egug*, *Romani*, *Fatima* (all belong to *gypsy*). It should be noted that, despite an impressive size of IS2 in these species, not a single LTR retrotransposon family has been found to be present in them as a full-length element: most of them occur as truncated or degenerate copies. This patchwork-like arrangement suggests that IS2 has undergone multiple recombination events throughout evolution and that no transposable element has been inserted in this locus since a recent past.

The available complete versions of sequenced genomes of several Triticeae species make it possible to trace back the evolution of the *CENH3* locus in better detail than we can do to other tribes and to assess the contribution of wild progenitor species to the formation of this locus in the corresponding cultivated derivatives. In cultivated barley *H. vulgare*, the *CENH3* locus is located on chromosome 1H and occupies almost 100 kb (Fig. 6a and b). A characteristic feature of the locus is that *βCENH3* is in the opposite direction to *αCENH3*, which is indicative of an inversion. As a result of the inversion, *βCENH3* and *αCENH3* assume a head-to-head orientation, and this is typical of all other Triticeae species, making them different from Poeae species. Unlike barley, other Triticeae species do not have a stop codon in exon 3 in the 3′-end of the HFD and, therefore retain the full-length sequence of *βCENH3*. One more feature of the DNA composition of the *CENH3* locus in *H. vulgare* is a high abundance of *copia* elements in it – they are nearly four times as abundant as *gypsy* elements (Table 3). We measured their ratio in barley chromosomes 1Н and 6Н, each having one copy of *βCENH3*, and found it to be 2.05:1, which is equal to the value determined for the entire barley genome [48]. Thus, the *CENH3* locus in *H. vulgare* has an unusually high concentration of *copia* elements, substantially higher than their genome-average abundance in this species. The dominant *copia* family of LTR retrotransposons at the *CENH3* locus, as in the entire *H. vulgare* genome, is *BARE-1*, with its 7 truncated elements contributing to 19.1 kb of the locus or 40.6% of all *copia* elements at the locus (Additional file 1: Table S4). Two other *copia* families, *Maximus* and *Inga*, are represented by fragments: six in the former (17.7 kb in total) and four in the latter (10 kb in total). The *CENH3* locus is half as long in wild barley *H. spontaneum* as in cultivated barley (Fig. 6b), and the *βCENH3* gene, together with its 5′-region, is in an inverted orientation, as in *H. vulgare*. The locus has many more fragments of *gypsy* elements in *H. spontaneum* than in *H. vulgare*. The composition of retrotransposon families and LINE families in both barley species are identical; however, *Maximus* is represented in the locus of *H. spontaneum* only by one short fragment, 220-bp in length, and *BARE-1*, by two short fragments, each about 900 bp in length (Additional file 1: Table S4).

**Table 3:** The percentage abundance of the main DNA classes in the intergenic spacers of the *CENH3* locus in Triticeae

Species	*Copia* superfamily	*Gypsy* superfamily	*Gypsy/ Copia*	Transposons	Others
*Hordeum spontaneum*	27.2	21.4	0.79	7.9	6.4
*Hordeum vulgare*	50.9	13.0	0.25	3.0	3.4
*Triticum urartu*	28.9	15.8	0.55	0	14.4
*Triticum aestivum,* A genome	19.4	57.5	2.96	1.8	7.3
*Aegilops speltoides*	0	1.5		13.7	23.1
*Triticum aestivum,* B genome	22.0	34.1	1.55	7.0	4.7
*Aegilops tauschii*	23.2	44.3	1.91	2.8	1.2
*Triticum aestivum,* D genome	22.2	42.4	1.91	0.5	1.0

NOTE. *Note*: Only Triticeae species are presented, because only these species, except for *Aegilops speltoides*, have been identified as having upwards of 60% DNA sequences in the most common classes and superfamilies of transposable elements. The *Others* column is for non-LTR retrotransposons, simple repeats and unclassified sequences

A comparison of the DNA composition at the *CENH3* locus in the progenitor species of each of the three genomes of cultivated wheat *T. aestivum* (ABD) and the corresponding genomes of hexaploid wheat itself yields a more complicated and perplexing result. Due to longer IS2, the *CENH3* locus is twice as large in the A genome of *T. aestivum* as in the candidate progenitor species *T. urartu*. While the respective sets of retrotransposon *copia* families and LINE families in both species are absolutely identical (Additional file 1: Table S4), the ratio of *gypsy*: *copia* elements sharply increases in favor of *gypsy*, from 0.55 y *Triticum urartu* to 3.0 in the A genome of *T. aestivum* (Table 3), due to three *Fatima* elements, with one of them as a full-length entity. Here we note the presence of the *copia* family *WIS*, which is the commonest in various wheat species. A comparison of the *CENH3* locus in the D genome of *T. aestivum* and in its candidate progenitor species, *Aegilops tauschii,* revealed an amazing similarity not only in the composition of retrotransposon families and LINE families, but also in the contribution each family makes and in the size of each intergenic spacer.

A comparison of the *CENH3* locus in the B genome of *T. aestivum* and in its candidate progenitor species *Ae*. *speltoides* yields quite a different result. The size of this locus in *Ae*. *speltoides* is 17.5 kb, and its DNA composition is similar to that of the loci in Stipeae and Brachypodieae. IS2 contains mainly LINE, groups of simple repeats, and short fragments of DNA transposon families. The *CENH3* locus in the B genome of *T. aestivum* is giant, 30 times as large as it is in the *Aе*. *speltoides*, which is because of an increase in the size of IS2 (Fig. 6b). IS2 is enriched for retrotransposon families, while the number of *gypsy* elements is only 54% higher than that of *copia* elements, which is in contrast to the IS2 situation in the A genome. IS2 of the B genome contains four full-length copies of *copia* elements and five full-length copies of *gypsy* elements (Additional file 1: Table S4), along with numerous fragments of the same families, diverse in length and nested within one another. Additionally, there are numerous fragments of LINE and various DNA transposon families, diverse in length, too. A specific feature of the DNA composition in IS2 is the presence of 46 short fragments of the *CACTA* transposon family, totaling as few as 17,550 bp. *CACTA* is the commonest transposon family in wheat genomes; however, it is absent from the *CENH3* locus in the A and D genomes. The size and DNA composition of the *CENH3* locus in the B genome of *T. aestivum* are indicative of extensive recombination involving insertion of TEs during the evolutionary and breeding processes acting on this genome in *T. aestivum*. The insertion events had been taking place for a long period of time, as the presence of both full-length and truncated copies suggests.

## Discussion

### Molecular mechanisms of *CENH3* duplication

Gene duplication is very common in plants, especially among angiosperms or flowering plants [49]. An important role has been attributed to gene duplications in generating evolutionary novelty and adaptation [50]. They come in either of two main ways: through polyploidization, when the whole genome is doubled (WGD), and due to local gene duplication or duplication of small sections of the genome, small-scale duplication (SSD). Several WGD events in the evolution of angiosperms have been described [51–53] and a nonrandom pattern of genome duplications has been shown to occur over time with many WGDs clustering around the Cretaceous–Paleogene extinction event about 66 million years ago [54]. WGDs are normally accompanied by subsequent loss of genes or more extended genome regions and genomic rearrangements [55]. Until now, it was known that the ancestors of the Triticeae species with two paralogous *CENH3* genes (barley, rye, wheat) and the cereal species closest to them with one copy of *CENH3* (rice, maize) split around 60 Mya [32] and belong to different subfamilies. There is every reason to claim that the *CENH3* locus, which consists of the syntenic genes *CDPK2*, *αCENH3* and *bZIP*, is of ancient origin and emerged around 50 Mya (Fig. 1). In the maize genome, the *CENH3* gene occurs as a single copy and has a different environment, which probably emerged due to deletions and rearrangements of a considerable part of the duplicated genome. In all Pooideae species that we have studied, *βCENH3* resides within the locus, which excludes the WGD assumption.

Another hypothesis about the origin of several *CENH3* paralogs stems from a vague understanding of how the *CENH3* gene emerged in LECA (the last eukaryotic common ancestor). A study of the evolutionary history of 159 samples of histone H3 and CENH3 variants showed that the H3 variants evolved independently within the related species of almost all eukaryotic supergroups [1]. All core histone types are encoded in the genome of a basal dinoflagellate [1], although dinoflagellate chromatin is not organized into nucleosomes [56]. The analysis carried out by Postberg et al. [1] leaves open whether a protoCENH3 ancestral to all eukaryotic CENH3s had existed or whether extant CENH3s have multiple origins in eukaryotic evolution. The differences in the exon-intron structures of the genes encoding different variants make it even more difficult to trace the evolutionary origin of the histone H3 family. All variants of the canonical histone, Н3.1, H3.2 and Н3.3, share more than 95% identity [4]; Н3.3 genes revealing introns – three in *P. virgatum* [12], while canonical histone Н3 (Н3.1) has none. In most *CENH3* gene duplications known in plant species, both paralogs have the same number of exons [17, 19, 20], although their number varies across species. In these cases, duplication may have occurred due to homologous recombination involving unequal-crossing over or gene conversion, as has been shown for duplications of *Cid,* a counterpart of *CENH3* in Drosophila [57].

Our comparison of the structure of the *CENH3* paralogs in cereals revealed differences in the exon-intron structure (Fig. 4), which resemble the differences in the structure of the variants of canonical histone H3 and obscure the mechanism of the duplications that have occurred in Pooideae evolution. Considering all the possibilities (WGDs, the independent origin of the paralogs from the corresponding variants of protoCENH3, SSD), we believe that the tandem duplication originating from unequal crossing-over is the most likely mechanism of duplication in the study species. This view is strongly supported by a small distance between the paralogs in *Stipa* and *Brachypodium* (Fig. 6). It is likely that duplications had more complex mechanisms in *Stipa* and *Brachypodium* species than in any other plant taxon [17, 19, 20]. In cereals, the duplication was accompanied by splicing abnormalities, leading to changes in the exon-intron structure and long deletions in the N-terminal domain of the protein βCENH3. The exon-intron structure of *βCENH3* is preserved in all subsequent species (Fig. 4b). This is favored by negative (purifying) selection, which acts on both paralogs – to different extents, though. Although selective pressure exerted on *βCENH3* is lower, it is still sufficient for the daughter copies and their exon-intron structure to have been preserved for 35–40 Муr. Our argument is consistent with data on the structure of the centromere-specific histones in mosquito, *mosqCid1* and *mosqCid2*, which are subject to distinct selective pressures, have highly divergent N-terminal tails and have been coretained for over 150 Myr [58].

However, it is quite likely that DNA-mediated duplication is not the only mechanism for an increase in the number of *CENH3* copies in cereal genomes. In *H. vulgare*, *αCENH3* and *βCENH3* form part of the *CENH3* locus and localize on chromosome 1H. The emergence of yet another copy of *βCENH3* on chromosome 6Н due to an independent duplication is difficult to explain by the recombination mechanism. It is more logical to assume that this additional copy arouse as a result of an SSD event with the involvement of a messenger RNA intermediate, by analogy with the phenomenon observed and described in *Arabidopsis thaliana* embryogenesis [59]. Analysis of the functional divergence of a large number of duplicated genes in cereals [60] showed that neofunctionality often occurs in daughter copies and is associated with the mechanism driving RNA-mediated duplication in a way similar to observations in *Drosophila* [61]. The most likely explanation for this is that when this mechanism is a factor the daughter copy moves to other chromosomes and appears in a different genomic environment and, consequently, under a different regulatory system.

### Species-specific evolution of the *CENH3* locus

Our analysis of the genomic environment of the *CENH3* genes in 23 Poaceae species suggests that the duplication that led to the new functional paralogs of the *CENH3* gene first occurred in the tribes Stipeae and Brachypodieae. This is confirmed by the presence of only one copy of this gene in the preceding tribes in the phylogenetic trees, statistically significant evidence of diversifying selection of the *βCENH3* genes on the branches of *S. sibirica*, *S. breviflora* and *B. sylvaticum* and the proximity of the paralogs at the *CENH3* locus in these species. The subfamily Pooideae, which has species in the six tribes that we have used in our study, including Triticeae and Poeae, formed 40–45 Mya [62], while Brachypodieae split off 35 Mya [36, 62]. The exact age of the other three tribes, Nardeae, Meliceae and Stipeae, is unknown to us; what we know is that, according to Soreng et al. [22], they split off from their common Pooideae ancestor before Brachypodieae (Fig. 1). Because there is not a single Stipeae species with both a genomic and a transcriptome library developed for it, we have yet to be able to determine the exon-intron structure of the *CENH3* paralogs. Thus, our assumption about an independent duplication of the *CENH3* gene in Stipeae is based on the observation that its paralogs display much higher sequence homology in *S. sibirica* and *S. breviflora* than in any other Pooideae species (Table 2). In the ML tree, clusters with αCENH3 and βCENH3 in *S. sibirica* and *S. breviflora* lie adjacent to each other, indicating a high structural similarity between these CENH3 forms in these species (Fig. 3). The Stipeae species have specific amino acid motifs in βCENH3, which Brachypodieae, Triticeae and Poeae do not (Additional file 2: Fig. S4). The further evolution of the *CENH3* locus from Brachypodium to cultivated species of barley and wheat displays a general tendency towards its expansion due to an increasing size of IS2 (Fig. 6b), which in the overwhelming majority of species correlates with increases in genome size. The main contributors to the increased size of IS2 and the *CENH3* locus are TEs, primarily those in the main superfamilies of LTR-containing retrotransposons. This general tendency has specific features in Triticeae and Poeae and in each species within these tribes. These features can be more distinctly traced by considering Triticeae species, for their genomes are the best studied.

The *βCENH3* inversion that we found in the *CENH3* locus of the barley species is, in our experience, a Triticeae-only feature and occurred in their common ancestor approximately 15–16 Mya [63]. Invasion by mobile elements and concomitant rearrangements in the structure of the *CENH3* locus took place in an independent way during the formation of each species’ genome. As a result of these processes, we observe significant variations in the size of the locus, the set of TE families and the amount of changes in their structure. The hands-down champion in all parameters is the locus in the B genome of *T. aestivum*, its size being almost 293 kb, of which 285 kb is contributed by IS2 (Fig. 6b). Researchers consider five *Aegilops* L. species in the Sitopsis section—*Ае. speltoides*, *Ае. longissima*, *Ae. sharonensis*, *Ae. bicornis* and *Ae. searsii*—to be putative donors of the B genome in *T. aestivum*, and *Ae*. *speltoides* is often considered as one of the main donors [64]. However, as far as the *CENH3* locus is concerned, with IS2 as short as 2.86 kb, this species has contributed virtually nothing to IS2 in the B genome of *T. aestivum*. Our results tend to support the hypothesis of gene flow between all *Aegilops* L. species in the Sitopsis section and the В genome of wheat [65, 66]. It seems logical to assume that distant hybridization, which is considered as a stress factor that intensifies recombination, the multiplication and activity of TEs in the genomes, was the reason for such a rapid expansion of the *CENH3* locus in the synthetic B genome and its enrichment of mobile elements. This assumption is also confirmed by the size of this locus in the cultivated barley *H. vulgare* and in the A genome of wheat *T. aestivum*, which is 1.75 and 1.83 times larger than that in their wild progenitors, *H. spontaneum* and *T. urartu*, respectively. However, a comparison of the structure of the *CENH3* loci in the D genome of *T. aestivum* and in its putative progenitor *Ae*. *tauschii* shows a similarity in the size of the locus, intergenic spacers and the composition of TE families (Additional file 1: Table S4) and questions the universality of such a scenario. Additionally, the wheat A, B, and D genomes are estimated to have diverged at about the same time, between 2.5 and 4.5 Mya [67], while allopolyploidization occurred 0.5 Mya [67, 68]. While the size of IS2 is subject to substantial variation, it is really surprising to observe that the sizes of two other intergenic spacers of the *CENH3* locus, IS1 and especially IS3, are relatively highly conserved. Only IS1 in the Poeae species *D. glomerata* and *A. sativa*, in which *βCENH3* has undergone a duplication event, defies this tendency.

Analysis of the representation of TE families at the *CENH3* locus in different species provides additional evidence in favor of the independent formation of this locus in different lineages. It has been shown many times that the *gypsy*-like retrotransposon families contribute about twice as much to the plant genomes as the *copia*-like families [48, 69, 70]. In the genomes we have looked into, only in the *CENH3* locus of the A genome *T. aestivum* this ratio being equal to 3.0. In all the other genomes, the *CENH3* locus is to varying degrees enriched for *copia*-like families. This is best observed in the *H. vulgare* and *T. urartu* genomes, to which the *copia* families contribute, respectively, four times and twice as much as the *gypsy* families (Table 3). A large number of rearranged and truncated elements indicates that invasion of the *CENH3* locus by TEs has occurred many times during the formation of each species’ genome. For example, in IS2 of *H. vulgare*, fragments of the two most common *copia*-like elements, *BARE1* and *Maximus*, make up about half of the locus, *BARE1* being represented by two full-size copies. *Maximus* is not represented by full-size copies; however, six truncated fragments of this element in IS2 of *H. vulgare* (Additional file 1: Table S4) total nearly as much as two full-size copies of *BARE1*. It is most likely that *Maximus* invaded the *CENH3* locus well before *BARE1*. The *CENH3* locus in the wild progenitor of cultivated barley, *H. spontaneum*, whose genome is more ancient, is devoid of full-length copies of retrotransposons.

Analysis of the gradient of various classes of TEs in intergenic spacers revealed yet another interesting tendency. Intergenic spacers of the *CENH3* locus in rice reveal simple repeats and short DNA fragments of non-autonomous transposons *Harbinger* and *Mariner*. The intergenic spacers in the loci in Stipeae and Brachypodieae species are composed of about the same classes of DNA sequences. *Stiрa sibirica* has all these components and additionally non-LTR retrotransposon LINE. LTR retrotransposons appear in IS2 in Triticeae and Poeae, and their insertion sites begin and end several kilobases away from genes. This gradient of different classes of TEs in the vicinity of coding genes is—or at least appears to be—a common rule, because it is characteristic of the entire barley genome [48].

## Conclusion

The following are the most pivotal stages in the emergence and formation of a two-component system of encoding the centromere-specific histone CENH3 in cereals. The syntenic group or the *CENH3* locus with the *CENH3* gene and the boundaries defined by *CDPK2* and *bZIP* first appeared around 50 Mya in a common ancestor of the subfamilies Bambusoideae, Oryzoideae and Pooideae. This locus came to Pooideae, which split off ca. 40–45 Mya, with a single copy of *CENH3* in the most ancient tribes Nardeae and Meliceae. The *βCENH3* gene as a part of the locus appeared in the tribes Stipeae and Brachypodieae, which split off 35 Mya. The duplication was accompanied by splicing abnormalities, leading to changes in the exon-intron structure and long deletions in the N-terminal domain of the protein βCENH3. Purifying selection acts mostly on αCENH3s, while βCENH3s and especially their NTT domains form more heterogeneous structures, in which clade-specific amino acid motifs are present. In barley species, the *βCENH3* gene assumed an inverted orientation relative to *αCENH3* and the left-border gene, *CDPK2*, was substituted with *LHCB-l*. А head-to-head orientation of the paralogs is a distinctive feature of all Triticeae species studied. As the evolutionary and domestication processes went on, the locus was growing in size due to an increasing distance between *αCENH3* and *βCENH3*. In turn, the distance increased due to a massive insertion of elements of the main LTR-containing retrotransposon superfamilies, *gypsy* and *copia*, without any evolutionary preference on either of them. A comparison of the molecular structure of this locus in the A, B and D subgenomes of the hexaploid wheat *T. aestivum* and in candidate donor species showed that invasion by transposable elements and concomitant rearrangements in the structure of the *CENH3* locus took place independently even in evolutionarily closely related species.

## Materials and methods

### Choosing cereal species for identification of *CENH3* orthologs and paralogs in sequenced genomes

Cereal species were chosen for identification of orthologous and paralogous genes encoding centromeric histone CENH3 based on the positions these species take in the worldwide phylogenetic classification of the family Poaceae [22] and if their genome assemblies, whether full or partial, were annotated in GenBank (https://www.ncbi.nlm.nih.gov/nuccore/) and Phytozome (https://phytozome-next.jgi.doe.gov/). A total of 23 species were included in the analysis (the list is given in Table 1); their phylogenetic relationships under the classification system of Soreng et al. [22] are presented in Fig. 1. If the databases had only partial genome assemblies (for *Stipa sibirica and Lolium perenne*), the available reads were downloaded from the SRA at NCBI (https://www.ncbi.nlm.nih.gov) and assembled into contigs (Additional files 3 and 4) using the assembly software Minia [26]. The search for *CENH3* sequences in full-length genomic sequences or in the contigs assembled was done using *TBLASTN* or *BLASTN* from the AB-*BLAST* suite [71] (https://blast.advbiocomp.com). The sequence of the DNA encoding protein *CENH3* or the sequence of this protein itself in a given species or in its nearest taxonomic neighbors was used as a query.

### Analysis of the *CENH3 genes and transcripts* in *Brachypodium distachyon*

For the analysis of the genomic region containing the sequences of the *CENH3* genes from the annotated genome of *Brachypodium distachyon* (GenBank NC_016132.3) and the structures of their potential transcripts, we used line Bd21 seeds (kindly provided by Dr. N. Collins, University of Adelaide, and Dr. Y. Shavrukov, Flinders University, Australia). Total RNA was extracted from young seedlings of Bd21 plants with TRI Reagent (MRC Inc., United States) and treated according to the manufacturers’ recommendations with the DNA-*free* Kit (Thermo Fisher Scientific) containing DNase. RNA was reverse-transcribed to cDNA with the SuperScript IV Reverse Transcriptase (Thermo Fisher Scientific). Based on the annotated sequence of transcript LOC100830307, ​​the following primers were designed for the putative paralogous genes: 5′-TGGCCCGCACGAAGCG − 3′ and 5′-CCTGTGCCCACTGATACGCC-3′ (primers 1 and 2 for *αCENH3*); 5′-ATGGCTCGCACCAAGCA-3′ and 5′-TGGATGGCCAAGAGATTCGC-3′ (primers 3 and 4 for *βCENH3*). The following PCR conditions were used: 3 min at 95 °C followed by 35 or 40 cycles at 94 °C for 1 min, 59 °C for 70 s, and 72 °C for 90 s, with a final extension for 5 min at 72 °C. RT-PCR products were cloned using the InsTAclone PCR Cloning Kit (Thermo Fisher Scientific). Both strands of 15–20 clones of each *CENH3* paralog were sequenced using the BigDye Terminator v3.1 Cycle Sequencing Kit and the ABI 3130 × 1 Genetic Analyzer (Applied Biosystems Inc., CA).

### Phylogenetic analysis

The genomic sequences of *αCENH3 and βCENH3* obtained for 23 species and the deduced protein sequences were used for phylogenetic analysis. Multiple alignments were performed using Muscle [40] or Bali-Phy [41] to identify taxon- and species-specific features in the protein structures. Homology searches and pair-wise alignments were performed using FASTA [72]. The type of selective pressure acting on the *CENH3* genes was determined by estimating the ratio of synonymous (Ks) to nonsynonymous (Ka) nucleotide substitution rates (ω). Estimation of ω from all-to-all pair-wise sequence comparisons was performed by KaKs_Calculator, Version 2.0 [46].

The evolutionary history was inferred by using the Maximum Likelihood method and JTT matrix-based model [42]. The tree with the highest log likelihood (− 4953.02) is shown. The percentage of the trees with the associated taxa clustered together is shown next to the branches. Initial tree(s) for the heuristic search was obtained automatically with Neighbor-Join and BioNJ algorithms applied to the matrix of pairwise distances estimated using a JTT model, and then by selecting the topology with the superior log likelihood value. Differences in evolutionary rates among sites were modeled using a discrete Gamma distribution (5 categories (+*G*, parameter = 1.0416)). The tree was drawn to scale, with branch lengths measured in the number of substitutions per site. The analysis involved 45 amino acid sequences. The final dataset contained 200 positions. Evolutionary analyses were conducted in MEGA X [43]. Bootstrap values were calculated from at least 1000 replications. Amino acid sequences were aligned using MUSCLE with default settings.

Tests for selective pressure and positive selection on the individual branches of the phylogenetic trees were performed using aBSREL, which infers the optimized number of ω rate categories per branch [73]. Complementary transcripts of the *αCENH3 and βCENH3* sequences were codon-aligned using MUSCLE [40] and then uploaded to and analyzed on the DataMonkey server (http://datamonkey.org/).

### Determination of the exon-intron structure

The sequences of the *αCENH3* and *βCENH3* transcripts were determined using an approach similar to the one described above, which was used for the search for the sequences of these genes. In the absence of the whole-genome sequences of a given species, RNA-seq reads were downloaded from the SRA at NCBI (the accessions numbers are given in Additional file 1: Table S1) and assembled into transcript contigs using *Minia* [26] and/or Trinity [74]. *In the assemblies, CENH3 transcripts* were found and analyzed using *TBLASTN*. The lists of the assembled *mRNA αCENH3* and *mRNA βCENH3* sequences are presented in Additional files 5 and 6. The lists of the deduced amino acid sequences of the αCENH3 and βCENH3 proteins are presented in Additional files 7 and 8.

To determine the exon-intron structure of the *αCENH3 and βCENH3* genes, RNA-seq reads from the SRA at NCBI were mapped onto the corresponding reference genome sequences or the assembled contigs of genome sequences (if the whole-genome reference sequences were not available) using HISAT2 [75] and STAR [76]. The output files in SAM format were visualized and subjected to a primary analysis using Geneious 11.0.2 (http://www.geneious.com) [77]. Analyses of the exon-intron structure, splice sites and read coverage were performed using StringTie [78] and Trinity [74].

### Identification of the *CENH3* locus in cereal genomes

The primary search for and localization of the genome copies of *αCENH3* and *βCENH3* were carried out on the best-studied and annotated genomes, *T. urartu* and *O. sativa*. We downloaded these genomes from NCBI and found homologous sequences in *T. urartu* spaced 20 kb apart, using BLASTN with the sequences of *αCENH3* and *βCENH3* genes in rye as queries [70]. Rice was found to have homology only with *αCENH3*. We examined the genomic environment of the *CENH3* genes and found that upstream of *βCENH3* are the *LHCB-l* gene, which encodes сhlorophyll a-b binding protein 3C (EMS53958) in *T. urartu*, and the *CDPK2* gene, which encodes calcium-dependent protein kinase 2 (XP_015638740.1) in *O. sativa*. Downstream of *αCENH3* both species have a *bZIP* transcription factor-encoding gene (XP_015638736.1). We used the genome sequences of *αCENH3, βCENH3* and the neighboring genes as markers of the *CENH3* locus and identified these syntenic genes in the genome sequences of the other cereal species. The composition of repeated DNA sequences in the *CENH3* locus in different species was determined using the *Viridiplantae RepeatMasker* program and database [73]. The genes neighboring to *CENH3* were identified by homology with the sequences of *T. urartu* and *O. sativa* using TBLASTX at NCBI or, locally, BLASTN*.*

## Supplementary Information

**Additional file 1 Table S1.** Sources of the genome sequences and transcripts of the *CENH3* locus genes used for analysis and contig assembly. **Table S2.** Estimates of ω values from all-to-all pair-wise comparison of *αCENH3* coding sequences. Values of Ka and Ks are shown above the diagonal. The ω values are shown below the diagonal followed by *P*-values in brackets. **Table S3.** Values of Ka and Ks are shown above the diagonal. The ω values are shown below the diagonal followed by P-values in brackets. Values of Ka and Ks are shown above the diagonal. The ω values are show below the diagonal followed by P-values in brackets. Values ω > 1 are highlighted in bold. **Table S4.** The presence of the most well-represented transposable element families in ISs in Triticeae species. The total number of base pairs for given superfamily of transposable elements is shown in bold. The values after the family names are (1) the percentage abundance of the family fragments in the superfamily and (2) the numbers of the family fragments. The number of asterisks (*) represents the number of full-length copies of the element.

**Additional file 2 Fig. S1.** *CENH3* nucleotide sequences from barley species virtually translated into proteins and aligned. Accession numbers are given in Table S1. The sequence of canonical histone Н3.3 in *H. vulgare* was retrieved from GenBank (accession number 769350). **Fig. S2.** An amino acid alignment inferred using the genomic sequences of three Brachypodium species (accession numbers are given in Table S1). Asterisks stand for stop codons in the sequences of sample 2 and sample 4. The aberrant transcript of *B. stacei*_1 was predicted by mapping the transcriptome of *B. stacei* (SRR4094449, SRR4094447, DRR090154) onto its genome using HISAT2 [75] followed by an analysis of the transcripts with StringTie [78]. **Fig. S3.** Experimental confirmation of the presence of a stop codon in exon 2 of *βCENH3* in *B. distachyon*. 1. A schematic of locus LOC100830307 retrieved from GenBank. 2. A schematic of a 1776-bp transcript from the locus. Exons appear as white finger-post arrows. 3. A schematic of sequences in locus LOC100830307 homologous to *βCENH3* and *αCENH3* in the rye genome [25]. 4. Products of RT-PCR with primers of 1 and 2 for *αCENH3* and primers 3 and 4 for *βCENH3*. Arrows point to the primer positions, stop codons are shown by cross. The upper scale shows the dimensions in bp. **Fig. S4.** A multiple alignment of the amino acid sequences of the CENH3 proteins using the Bali-Phy program [41]. BAli-Phy is software that estimates multiple sequence alignments and evolutionary trees from DNA, amino acid, or codon sequences. It uses likelihood-based evolutionary models of substitutions and insertions and deletions to place gaps. Redelings showed that BAli-Phy had 3.5 times fewer alignment errors than MUSCLE and MAFFT on simulated data [41]. For convenience, the alpha and beta forms are grouped into two separate blocks: 1–26 (αCENH3) and 27–45 (βCENH3). Amino acid residues identical in all species are shown as white letters on the black background. Amino acid motifs specific for certain tribes and genera are contained within rectangular boxes with different border colors. **Fig. S5.** Branch-site (BSREL) analysis of positive selection for the αCENH3 (**a**) and βCENH3 (**b**) [47]. The upper scale indicates the length of branches. Branch lengths are scaled to the expected number of substitutions per amino acid and branch colors indicate the strength of selection (dN/dS or ω). Green, positive selection (ω > 5); black, purifying selection (ω = 0); gray, neutral evolution (ω = 1). The proportion of each color represents the fraction of the sequence undergoing the corresponding class of selection. Branch thickness reflects the amount of statistical support for evolution under episodic diversifying positive selection as determined by BSREL. The tables show the branches and nodes in which BSREL found evidence of episodic diversifying selection at *P* < 0.05.

**Additional file 3 **The DNA sequence of the *CENH3* locus of *Stipa sibirica* assembled in this study.

**Additional file 4 **The DNA sequence of the *CENH3* locus of *Lolium perenne* assembled in this study.

**Additional file 5 **The sequences of mRNA *αCENH3* assembled for this study.

**Additional file 6 **The sequences of mRNA *βCENH3* assembled for this study.

**Additional file 7.** The amino acid sequences of αCENH3 assembled for this study.

**Additional file 8.** The amino acid sequences of βCENH3 assembled for this study.

## Data Availability

The datasets underlying this article are available as supplementary information in the article and in its online supplementary material. The data will be shared on request to the corresponding author.

## Abbreviations

LTR: Long terminal repeat
Mya: Million years ago
SSD: Small-scale duplication.
WGD: Whole-genome duplication.
