# Supplementary material for: The origin and evolution of a two-component system of paralogous genes encoding the centromeric histone CENH3 in cereals
Source: BMC Plant Biol. 2021 Nov 18;21:541. doi: 10.1186/s12870-021-03264-3 (PMC8603533; doi:10.1186/s12870-021-03264-3)
Supplement: Supplementary file 1 — Additional file 1 Table S1. Sources of the genome sequences and transcripts of the CENH3 locus genes used for analysis and contig assembly. Table S2. Estimates of ω values from all-to-all pair-wise comparison of αCENH3 coding sequences. Values of Ka and Ks are shown above the diagonal. The ω values are shown below the diagonal followed by P-values in brackets. Table S3. Values of Ka and Ks are shown above the diagonal. The ω values are shown below the diagonal followed by P-values in brackets. Values of Ka and Ks are shown above the diagonal. The ω values are show below the diagonal followed by P-values in brackets. Values ω > 1 are highlighted in bold. Table S4. The presence of the most well-represented transposable element families in ISs in Triticeae species. The total number of base pairs for given superfamily of transposable elements is shown in bold. The values after the family names are (1) the percentage abundance of the family fragments in the superfamily and (2) the numbers of the family fragments. The number of asterisks (*) represents the number of full-length copies of the element. [file 12870_2021_3264_MOESM1_ESM.pdf]

Table S1. Sources of the genome sequences and transcripts of the *CENH3* locus genes used for analysis and contig assembly.

Species	Sources of genome sequences	Acc. No.	Coordinates in nucleotides	Sources of transcripts (GenBank, NCBI), Acc. No.
Streptochaeta angustifolia	N/A			SRR12490852, SRR12490853, SRR12490854, SRR12490855, SRR3233339
Panicum virgatum	Pvirgatum_516_v5.0[1]	Chr03K<br>Chr03N	21160000-21220000<br>22075000-22150000	SRR1588141, SRR1588141, SRR5838951, SRR5838951, SRR3467191
Zea mays	GCF_000005005.2[2]	NC_024464	159300480-159450593	SRR9020473, SRR10201924, SRR8368670
Oryza sativa	GCF_001433935.1[2]	NC_029260		XP_015638738.1
Raddia distichophylla	QXEO01000121.1[2]	Scaffold559	2010000-2040000	
Nardus stricta	N/A			ERR1744596, ERR1744597, ERR1744598, ERR1744599, ERR1744600, ERR1744601, ERR1744602, ERR1744603
Melica nutans	N/A			SRR6127959, ERR1744588, SRR6127955, SRR6127931
Stipa sibirica	SRR8208359[2]	cenh3-stipa		
Stipa breviflora	N/A			SRR7152929, SRR7152933, SRR7152936
Brachypodium distachyon	GCA_000005505.4[2]	NC_0161323	19100000-19220000	SRR4094488, SRR5279890, SRR6940284, ERR3317433, ERR1744559, ERR1744553, ERR174455
Brachypodium sylvaticum	Bsylvaticum_490_v1.1[1]	chr8	6974000-7000000	SRR837756, SRR837757, SRR837755, SRR7814888, SRR7814877
Brachypodium stacei	Bstacei_316_v1.0[1]	chr08	4665000-4689000	DRR090154, SRR4094447, SRR4094449
Avena sativa	Avena sativa v1.0[3]	ctg8808		SRR5097463, SRR5097544, SRR5818212, SRR5818217, SRR6388184, SRR6388185
Dactylis glomerata	QXEO01000121.1[2]	Contig128	1150000-1250000	SRR6659677, SRR6659679, SRR7758699, SRR7758701
Lolium perenne	ERR1333456, ERR1333455[2]	cenh3-lolium		SRR5605275, SRR5605277, SRR10050124, SRR10050125
Hordeum brevisubulatum	N/A			SRR7817916, SRR7817918
Hordeum vulgare, ssp. *spontaneum*	WB_v0.5[4]	WB_00002249<br>WB_00001651	1-77466<br>237759-228880	ERR776122, SRR6687590, ERR776119, SRR6687582, SRR6687591, ERR776127,

				SRR6687600, SRR6687581, SRR6687615
Hordeum vulgare	GCA_902498975.1[2]	Chr1H<br>Chr6H	452790000-452920000<br>155820000-155930000	SRR1577845, SRR3290309, SRR10960787, SRR8426746, SRR6649468, SRR3628913
Secale cereale	FKKI010016211.1[2]	contig_17452		MG384772, MG384780
Triticum urartu	KD187944.1[2]	scaffold30245		KM507184, KM507181
Aegilops speltoides	TGAC_WGS_speltoides_v1[5]	contig_196639		ERR420226, ERR420227
Aegilops tauschii	GCF_001957025.1[2]	NW_017909796	535000-597000	SRR6281363, SRR5460240, SRR5460239, ERR2190543
Triticum aestivum	LS992080.1[2]	chr1A<br>chr1B<br>chr1D	498500000-498640000<br>542100000-542480000<br>403250000-403380000	SRR8413506, SRR6941181, ERR2174403

[1)] Phytozome: https://phytozome.jgi.doe.gov
[2)] NCBI: https://www.ncbi.nlm.nih.gov
[3)] The Oat Genome project: https://avenagenome.org/
[4)] NCGR: http://db.ncgr.ac.cn/wild_barley/index.php
[5)] URGI: http://wheat-urgi.versailles.inra.fr/

Table S2. Estimates of *ω* values from all-to-all pair-wise comparison of αCENH3 coding sequences.

**Species**	*Z. mays*	*T. urartu*	*S. sibirica*	*S. cereale*	*S. breviflora*	*S. angustifolia*	*R. distichophylla*	*P. virgatum*N	*P. virgatum* K
Z. mays	-	0.268 / 1.176	0.250 / 1.116	0.240 / 1.402	0.218 / 0.934	0.201 / 1.572	0.219 / 1.032	0.139 / 0.580	0.153 / 0.504
T. urartu	0.228 (1.95e-29)	-	0.276 / 0.721	0.025 / 0.104	0.207 / 0.626	0.240 / 0.992	0.240 / 0.818	0.235 / 0.944	0.255 / 0.833
S. sibirica	0.224 (4.23e-28)	0.373 (9.55e-11)	-	0.280 / 0.735	0.139 / 0.262	0.267 / 1.381	0.207 / 0.538	0.266 / 1.179	0.272 / 0.894
S. cereale	0.171 (1.67e-40)	0.236 (0.0004)	0.380 (3.20e-11)	-	0.210 / 0.617	0.255 / 1.292	0.235 / 0.879	0.219 / 1.041	0.243 / 0.860
S. breviflora	0.233 (5.02e-23)	0.331 (2.05e-11)	0.531 (0.0082)	0.340 (5.79e-11)	-	0.269 / 1.443	0.216 / 0.564	0.230 / 1.137	0.236 / 0.910
S. angustifolia	0.128 (4.74e-50)	0.260 (1.65e-19)	0.194 (1.43e-37)	0.197 (2.85e-34)	0.186 (2.66e-40)	-	0.224 / 1.116	0.207 / 1.193	0.226 / 1.082
R. distichophylla	0.212 (1.04e-25)	0.293 (3.54e-16)	0.385 (2.65e-08)	0.267 (9.72e-20)	0.374 (1.05e-07)	0.200(2.77e-29)	-	0.218 / 0.839	0.219 / 0.695
P. virgatum N	0.240 (1.07e-14)	0.249 (1.44e-22)	0.226 (1.05e-31)	0.211 (2.29e-28)	0.202 (1.79e-32)	0.173 (6.50e-35)	0.259 (2.92e-19)	-	0.041 / 0.096
P. virgatum K	0.303 (2.55e-10)	0.306 (1.09e-16)	0.304 (4.47e-18)	0.283 (1.85e-18)	0.269 (9.67e-22)	0.209 (1.30e-28)	0.314 (2.28e-13)	0.429 (0.0161)	-
O. sativa	0.226 (6.71e-23)	0.303 (6.52e-16)	0.323 (1.46e-16)	0.255 (6.35e-23)	0.320 (5.49e-13)	0.172 (1.67e-36)	0.302 (1.09e-12)	0.188 (9.21e-28)	0.279 (1.63e-15)
N. stricta	0.232 (2.17e-24)	0.448 (1.45e-08)	0.359 (1.85e-14)	0.395 (3.71e-12)	0.387 (6.57e-11)	0.244 (4.07e-28)	0.319 (3.84e-14)	0.290 (2.24e-15)	0.333 (2.20e-13)
M. nutans	0.210 (5.94e-29)	0.329 (5.87e-14)	0.392 (2.84e-08)	0.355 (4.51e-12)	0.275 (2.72e-12)	0.185 (6.66e-38)	0.405 (1.69e-06)	0.211 (1.23e-26)	0.306 (2.60e-15)
L. perenne	0.234 (1.27e-28)	0.469 (7.63e-06)	0.399 (2.45e-09)	0.406 (1.44e-08)	0.381 (2.81e-08)	0.194 (5.82e-32)	0.328 (1.03e-11)	0.275 (3.29e-21)	0.292 (7.68e-19)
H. vulgare	0.191 (1.48e-40)	0.682 (0.1467)	0.360 (4.08e-12)	0.665 (0.1432)	0.324 (2.66e-13)	0.227 (1.08e-28)	0.294 (3.47e-18)	0.223 (1.24e-28)	0.271 (1.02e-21)
H. spontaneum	0.189 (1.46e-40)	0.707 (0.1913)	0.346 (2.37e-13)	0.670 (0.0977)	0.313 (3.87e-14)	0.225 (1.23e-28)	0.292 (2.81e-18)	0.223 (1.26e-28)	0.270 (9.83e-22)
H. brevisubulatum	0.191 (8.14e-34)	0.456 (0.0108)	0.309 (1.08e-15)	0.356 (0.0008)	0.287 (8.90e-15)	0.228 (3.67e-26)	0.243 (4.21e-22)	0.219 (2.58e-25)	0.259 (8.82e-20)
T. aestivum D	0.205 (4.02e-34)	0.050 (0000)	0.389 (2.04e-10)	0.191 (6.72e-05)	0.339 (5.23e-11)	0.236 (2.28e-28)	0.288 (7.67e-17)	0.230 (2.04e-25)	0.283 (3.14e-19)
D. glomerata	0.184 (1.88e-35)	0.409 (2.86e-07)	0.237 (4.49e-18)	0.366 (3.16e-09)	0.229 (9.59e-16)	0.199 (5.47e-31)	0.304 (5.09e-12)	0.243 (7.68e-23)	0.237 (8.08e-24)
T. aestivum B	0.190 (1.67e-36)	0.284 (0.0036)	0.372 (3.56e-11)	0.235 (0.0010)	0.363 (7.09e-10)	0.223 (5.08e-27)	0.327 (3.49e-13)	0.238 (1.79e-24)	0.315 (3.04e-15)
B. sylvaticum	0.284 (1.24e-19)	0.233 (1.55e-19)	0.410 (1.73e-07)	0.230 (3.57e-20)	0.306 (1.15e-10)	0.144 (2.67e-48)	0.307 (1.23e-12)	0.188 (2.30e-31)	0.269 (4.61e-19)
B. stacei	0.262 (2.74e-22)	0.285 (2.73e-14)	0.411 (3.01e-07)	0.293 (3.92e-13)	0.374 (1.045e-07	0.133 (1.77e-44)	0.326 (6.02e-12)	0.193 (4.36e-29)	0.272 (3.66e-18)
B. distachyon	0.300 (6.16e-17)	0.271 (2.75e-15)	0.420 (3.30e-07)	0.241 (7.47e-19)	0.306 (1.24e-10)	0.160 (5.15e-36)	0.300 (1.43e-12)	0.306 (2.61e-27)	0.295 (1.35e-15)
T. aestivum A	0.228 (1.95e-29)	0.001 (0.0000	0.377 (5.05e-11)	0.218 (0.0001)	0.325 (1.04e-11)	0.256 (6.53e-21)	0.307 (3.88e-15)	0.260 (1.02e-21)	0.318 (3.25e-15)
A. tauschii	0.205 (4.03e-34)	0.050 (0.0000)	0.389 (2.04e-10)	0.191 (6.72e-05)	0.337 (5.23e-11)	0.236 (2.28e-23)	0.295 (1.09e-16)	0.230 (2.05e-25)	0.282 (3.14e-19)
A. speltoides	0.181 (4.59e-39)	0.137 (8.78e-06)	0.427 (3.59e-08)	0.181 (4.49e-05)	0.384 (1.86e-08)	0.249 (4.49e-18)	0.328 (3.43e-13)	0.228 (6.23e-25)	0.309 (3.10e-15)
A. sativa	0.225 (1.66e-28)	0.526 (0.0006)	0.442 (1.22e-06)	0.436 (1.65e-06)	0.421 (2.79e-06)	0.196 (1.07e-33)	0.394 (4.06e-08)	0.199 (2.15e-34)	0.268 (6.83e-22)

**Species**	*O. sativa*	*N. stricta*	*M. nutans*	*L. perenne*	*H. vulgare*	*H. spontaneum*	*H. brevisubulatum*	*T. aestivum* D	*D. glomerata*
Z. mays	0.207 / 0.917	0.236 / 1.017	0.226 / 1.077	0.268 / 1.146	0.273 / 1.429	0.269 / 1.423	0.237 / 1.242	0.262 / 1.274	0.232 / 1.264
T. urartu	0.247 / 0.816	0.324 / 0.722	0.250 / 0.759	0.226 / 0.481	0.126 / 0.184	0.122 / 0.173	0.073 / 0.160	0.003 / 0.063	0.214 / 0.524
S. sibirica	0.236 / 0.731	0.300 / 0.839	0.218 / 0.557	0.250 / 0.626	0.266 / 0.740	0.264 / 0.763	0.256 / 0.831	0.278 / 0.714	0.171 / 0.722
S. cereale	0.241 / 0.947	0.327 / 0.825	0.244 / 0.687	0.232 / 0.573	0.125 / 0.187	0.121 / 0.180	0.060 / 0.168	0.022 / 0.115	0.215 / 0.589
S. breviflora	0.210 / 0.656	0.287 / 0.740	0.145 / 0.526	0.199 / 0.523	0.228 / 0.703	0.225 / 0.717	0.202 / 0.702	0.209 / 0.618	0.138 / 0.603
S. angustifolia	0.224 / 1.291	0.296 / 1.212	0.258 / 1.392	0.226 / 1.166	0.268 / 1.181	0.262 / 1.164	0.246 / 1.077	0.238 / 1.010	0.248 / 1.241
R. distichophylla	0.185 / 0.611	0.233 / 0.729	0.183 / 0.451	0.213 / 0.649	0.261 / 0.886	0.257 / 0.880	0.220 / 0.904	0.239 / 0.830	0.193 / 0.633
P. virgatum N	0.175 / 0.926	0.215 / 0.741	0.202 / 0.958	0.263 / 0.955	0.247 / 1.105	0.242 / 1.084	0.212 / 0.968	0.231 / 1.004	0.228 / 0.937
P. virgatum K	0.192 / 0.688	0.239 / 0.718	0.229 / 0.751	0.260 / 0.890	0.265 / 0.978	0.260 / 0.962	0.223 / 0.862	0.250 / 0.885	0.232 / 0.978
O. sativa	-	0.219 / 1.035	0.179 / 0.628	0.230 / 0.673	0.223 / 0.942	0.217 / 0.940	0.208 / 0.947	0.246 / 0.902	0.212 / 0.805
N. stricta	0.212 (1.85e-28)	-	0.263 / 0.726	0.268 / 0.764	0.325 / 0.911	0.318 / 0.904	0.297 / 0.769	0.323 / 0.808	0.223 / 0.717
M. nutans	0.285 (7.00e-14)	0.362 (3.51e-12)	-	0.219 / 0.664	0.266 / 0.707	0.262 / 0.703	0.225 / 0.693	0.252 / 0.749	0.192 / 0.599
L. perenne	0.343 (8.70e-12)	0.351 (2.92e-13)	0.330 (7.01e-13)	-	0.243 / 0.621	0.239 / 0.636	0.226 / 0.532	0.230 / 0.518	0.153 / 0.344
H. vulgare	0.236 (3.32e-23)	0.356 (4.84e-15)	0.376 (1.79e-11)	0.391 (2.30e-09)	-	0.003 / 0.008	0.081 / 0.205	0.122 / 0.196	0.220 / 0.533
H. spontaneum	0.232 (1.01e -24	0.352 (2.08e-15)	0.371 (1.45e-11)	0.377 (5.23e-10)	0.354 (0.3755)	-	0.008 / 0.197	0.119 / 0.186	0.216 / 0.566
H. brevisubulatum	0.220 (1.81e-25)	0.386 (2.09e-11)	0.324 (3.85e-13)	0.425 (2.21e-07)	0.394 (0.0005)	0.391 (0.0008)	-	0.070 / 0.182	0.196 / 0.630
T. aestivum D	0.273 (6.89e-20)	0.400 (1.30e-11)	0.337 (1.29e-13)	0.443 (1.35e-06)	0.621 (0.0513)	0.640 (0.0995)	0.382 (0.0018)	-	0.217 / 0.528
D. glomerata	0.263 (7.95e-18)	0.312 (1.74e-13)	0.320 (2.92e-11)	0.446 (7.94e-05)	0.384 (1.80e-08)	0.382 (6.61e-09)	0.311 (9.51e-12)	0.410 (2.94e-07)	-
T. aestivum B	0.329 (1.78e-14)	0.419 (1.37e-10)	0.392 (6.66e-10)	0.406 (3.69e-08)	0.816 (0.4184)	0.850 (0.5109)	0.393 (0.0007)	0.177 (7.34e-05)	0.390 (1.07e-07)
B. sylvaticum	0.307 (2.32e-12)	0.289 (1.40e-19)	0.299 (5.71e-13)	0.228 (3.75e-13)	0.251 (4.65e-21)	0.249 (3.35e-21)	0.196 (1.86e-23)	0.247 (2.82e-18)	0.353 (7.54e-09)
B. stacei	0.305 (8.74e-12)	0.321 (1.62e-15)	0.320 (2.00e-11)	0.323 (2.28e-13)	0.299 (2.78e-15)	0.297 (2.06e-15)	0.249 (2.76e-16)	0.301 (3.21e-13)	0.320 (1.28e-10)
B. distachyon	0.306 (8.74e-12)	0.337 (5.74e-14)	0.268 (7.79e-15)	0.277 (1.16e-13)	0.268 (5.35e-19)	0.266 (4.07e-19)	0.221 (5.25e-19)	0.269 (5.88e-16)	0.313 (3.80e-11)
T. aestivum A	0.298 (1.10e-16)	0.468 (1.21e-07)	0.324 (1.08e-14)	0.460 (4.70e-06)	0.653 (0.1087)	0.674 (0.1437)	0.432 (0.0034)	0.043 (9.27e-05)	0.401 (1.62e-07)
A. tauschii	0.273 (6.89e-20)	0.400 (1.30e-11)	0.337 (1.29e-13)	0.443 (1.35e-06)	0.621 (0.0513)	0.640 (0.0995)	0.382 (0.0018)	NA / NA	0.411 (2.29e-07)
A. speltoides	0.295 (1.09e-16)	0.419 (1.43e-10)	0.368 (1.02e-10)	0.434 (1.65e-06)	0.696 (0.1875)	0.722 (0.2414)	0.341 (0.0002)	0.118 (8.61e-06)	0.408 (2.79e-07)
A. sativa	0.302 (1.04e-13)	0.380 (2.81e-11)	0.403 (3.58e-08)	0.418 (0.0001)	0.362 (2.10e-09)	0.349 (3.37e-10)	0.328 (6.20e-10)	0.531 (0.0006)	0.463 (0.0006)

**Species**	*T. aestivum* B	*B. sylvaticum*	*B. stacei*	*B. distachyon*	*T. aestivum A*	*A. tauschii*	*A. speltoides*	*A. sativa*
Z. mays	0.249 / 1.315	0.269 / 0.947	0.261 / 0.998	0.266 / 0.885	0.268 / 1.176	0.262 / 1.274	0.248 / 1.368	0.264 / 1.172
T. urartu	0.020 / 0.070	0.189 / 0.809	0.195 / 0.686	0.191 / 0.705	8.24e-06 / 0.008	0.003 / 0.063	0.015 / 0.106	0.217 / 0.413
S. sibirica	0.265 / 0.712	0.236 / 0.576	0.225 / 0.546	0.232 / 0.554	0.276 / 0.732	0.278 / 0.714	0.276 / 0.647	0.245 / 0.554
S. cereale	0.019 / 0.080	0.191 / 0.828	0.190 / 0.648	0.188 / 0.780	0.025 / 0.113	0.022 / 0.115	0.022 / 0.121	0.213 / 0.487
S. breviflora	0.218 / 0.601	0.172 / 0.561	0.182 / 0.486	0.169 / 0.552	0.208 / 0.638	0.209 / 0.618	0.212 / 0.550	0.202 / 0.480
S. angustifolia	0.238 / 1.070	0.220 / 1.525	0.222 / 1.667	0.210 / 1.314	0.241 / 0.941	0.238 / 1.010	0.234 / 0.939	0.243 / 1.242
R. distichophylla	0.238 / 0.729	0.212 / 0.692	0.218 / 0.668	0.204 / 0.679	0.240 / 0.783	0.239 / 0.830	0.239 / 0.728	0.224 / 0.570
P. virgatum N	0.227 / 0.955	0.218 / 1.158	0.210 / 1.085	0.214 / 1.037	0.235 / 0.906	0.231 / 1.004	0.219 / 0.962	0.254 / 1.281
P. virgatum K	0.253 / 0.801	0.234 / 0.869	0.227 / 0.836	0.231 / 0.782	0.255 / 0.803	0.250 / 0.885	0.238 / 0.773	0.263 / 0.983
O. sativa	0.255 / 0.775	0.203 / 0.661	0.189 / 0.617	0.200 / 0.654	0.248 / 0.832	0.246 / 0.902	0.239 / 0.810	0.211 / 0.699
N. stricta	0.325 / 0.777	0.279 / 0.963	0.273 /0.850	0.278 / 0.824	0.324 / 0.692	0.323 / 0.808	0.327 / 0.780	0.288 / 0.758
M. nutans	0.258 / 0.658	0.185 / 0.619	0.189 / 0.592	0.176 / 0.656	0.251 / 0.773	0.252 / 0.749	0.244 / 0.663	0.224 / 0.556
L. perenne	0.224 / 0/552	0.182 / 633	0.192 / 0.593	0.172 / 0.621	0.227 / 0.493	0.230 / 0.518	0.228 / 0.524	0.124 / 0.298
H. vulgare	0.129 / 0.157	0.228 / 0.911	0.228 / 0.763	0.227 / 0.848	0.126 / 0.193	0.122 / 0.197	0.122 / 0.175	0.210 / 0.581
H. spontaneum	0.125 / 0.147	0.225 / 0.902	0.225 / 0.757	0.223 / 0.839	0.123 / 0.182	0.119 / 0.186	0.119 / 0.164	0.203 / 0.582
H. brevisubulatum	0.076 / 0.194	0.172 / 0.878	0.179 / 0.717	0.169 / 0.768	0.073 / 0.169	0.070 / 0.183	0.070 / 0.205	0.183 / 0.557
T. aestivum D	0.017 / 0.097	0.191 / 0.773	0.198 / 0.656	0.194 / 0.720	0.003 / 0.071	NA / NA	0.112 / 0.098	0.221 / 0.416
D. glomerata	0.205 / 0.526	0.185 / 0.523	0.182 / 0.568	0.181 / 0.577	0.215 / 0.537	0.217 / 0.528	0.217 / 0.532	0.134 / 0. 289
T. aestivum B	-	0.187 / 0.770	0.190 / 0.648	0.184 / 0.723	0.020 / 0.078	0.017 / 0.097	0.017 / 0.069	0.214 / 0.440
B. sylvaticum	0.243 (1.14e-18)	-	0.022 / 0.068	0.014 / 0.055	0.189 / 0.826	0.191 / 0.773	0.191 / 0.708	0/198 / 0.587
B. stacei	0.293 (3.92e-13)	0.327 (0.0098)	-	0.027 / 0.070	0.196 / 0. 700	0.198 / 0.656	0.199 / 0.603	0.204 / 0.613
B. distachyon	0.255 (1.58e-16)	0.253 (0.0054)	0.381 (0.0344)	-	0.191 / 0.720	0.194 / 0.720	0.194 / 0.660	0.191 / 0.607
T. aestivum A	0.255 (0.0013)	0.229 (4.13e-20)	0.279 (1.26e-14)	0.265 (1.05e-15)	-	0.003 / 0.071	0.015 / 0.116	0.218 / 0.424
A. tauschii	0.177 (7.34e-05)	0.247 (2.82e-18)	0.301 (7.16e-13)	0.269 (5.88e-16)	0.043 (9.27e-05)	-	0.012 / 0.098	0.221 / 0.416
A. speltoides	0.245 (0.0081)	0.270 (2.21e-15)	0.329 (8.10e-11)	0.294 (3.21e-13)	0.128 (2.51e-06)	0.118 (8.61e-06)	-	0.219 / 0.420
A. sativa	0.486 (4.11e-05)	0.336 (3.60e-10)	0.332 (5.67e-11)	0.314 (2.11e-11)	0.513 (0.0002)	0.531 (0.0006)	0.521 (0.0006)	-

Note. Values of *Ka* and *Ks* are shown above the diagonal. The *ω* values are shown below the diagonal followed by P-values in brackets.

Table S3. Estimates of ω values from all-to-all pair-wise comparison of βCENH3 coding sequences.

**Species**	*T. urartu*	*S. sibirica*	*S. cereale*	*S. breviflora*	*L. perenne*	*H. vulgare 6H*	*H. vulgare 1H*	*H. spontaneum 6H*	*H. spontaneum 1H*	*H. brevisubulatum*	*T. aestivum D*	*D. glomerata*	*T. aestivum B*	*B. sylvaticum*	*T. aestivum A*	*A. tauschii*	*A. speltoides*	*A. sativa 2*	*A. sativa 1*
T. urartu	–	0,186/0,756	0,033/0,128	0,263/0,775	0,193/0,534	0,195/0,277	0,196/0,237	0,195/0,277	0,195/0,219	0,053/0,116	0,019/0,086	0,218/0,448	0,030/0,105	0,275/0,700	0,000/0,013	0,019/0,086	0,047/0,082	0,207/0,445	0,211/0,431
S. sibirica	0,246(2,73E-15)	–	0,188/0,895	0,134/0,366	0,253/0,785	0,286/1,191	0,284/0,838	0,286/1,191	0,282/0,811	0,213/0,877	0,192/0,875	0,283/0,745	0,201/0,892	0,177/0,578	0,187/0,771	0,192/0,875	0,205/0,769	0,273/0,766	0,284/0,815
S. cereale	0,258(0,001165)	0,210(6,85E-21)	–	0,260/0,918	0,193/0,516	0,181/0,358	0,191/0,297	0,181/0,358	0,190/0,280	0,050/0,172	0,029/0,110	0,225/0,504	0,030/0,122	0,277/0,907	0,033/0,141	0,029/0,110	0,058/0,074	0,203/0,467	0,208/0,451
S. breviflora	0,339(8,90E-12)	0,367(5,40E-06)	0,283(4,75E-17)	–	0,306/0,855	0,360/1,456	0,327/0,807	0,360/1,456	0,325/0,777	0,271/0,834	0,250/0,913	0,311/0,792	0,251/0,898	0,272/0,547	0,264/0,789	0,250/0,913	0,279/0,795	0,358/0,606	0,373/0,637
L. perenne	0,361(1,05E-07)	0,323(1,02E-12)	0,374(4,17E-07)	0,358(1,85E-12)	–	0,275/0,682	0,285/0,473	0,275/0,682	0,279/0,457	0,183/0,599	0,181/0,527	0,131/0,327	0,185/0,511	0,256/1,031	0,193/0,548	0,181/0,527	0,212/0,563	0,125/0,344	0,128/0,339
H. vulgare 6H	0,705(0,167867)	0,240(2,83E-23)	0,507(0,003461)	0,247(4,78E-28)	0,403(1,72E-07)	–	0,201/0,226	0,207/0,204	0,207/0,204	0,196/0,341	0,196/0,316	0,313/0,577	0,191/0,271	0,347/1,078	0,196/0,294	0,196/0,316	0,202/0,341	0,285/0,588	0,285/0,570
H. vulgare 1H	0,829(0,474312)	0,339(2,62E-10)	0,643(0,083173)	0,405(5,52E-08)	0,602(0,010539)	0,890(0,70384	–	0,201/0,226	0,006/0,015	0,162/0,272	0,182/0,276	0,362/0,413	0,175/0,248	0,381/0,695	0,198/0,254	0,182/0,276	0,207/0,279	0,294/0,411	0,312/0,417
H. spontaneum 6H	0,705(0,167867)	0,240(2,83E-23)	0,507(0,003461)	0,247(4,78E-28)	0,403(1,72E-07)	**1,013(0,999995)**	0,890(0,70384)	–	0,207/0,204	0,196/0,341	0,196/0,316	0,313/0,577	0,191/0,271	0,347/1,078	0,196/0,294	0,196/0,316	0,202/0,341	0,285/0,570	0,285/0,570
H. spontaneum 1H	0,889(0,585155)	0,347(6,18E-10)	0,678(0,117377)	0,418(1,21E-07)	0,611(0,015234)	**1,013(0,999995)**	0,422(0,082035	1,013(0,999995)	–	0,163/0,254	0,181/0,259	0,369/0,392	0,176/0,230	0,382/0,689	0,196/0,236	0,181/0,259	0,194/0,257	0,297/0,389	0,304/0,393
H. brevisubulatum	0,458(0,023422)	0,242(3,98E-18)	0,292(0,000838)	0,326(4,81E-13)	0,306(5,07E-10)	0,575(0,025668)	0,594(0,041908	0,575(0,025668)	0,641(0,097414)	–	0,051/0,158	0,221/0,531	0,042/0,143	0,271/0,693	0,053/0,129	0,051/0,158	0,067/0,141	0,209/0,529	0,221/0,516
T. aestivum D	0,219(0,002016)	0,220(1,86E-19)	0,263(0,002915)	0,274(1,60E-17)	0,344(8,47E-08)	0,619(0,059954)	0,659(0,150227	0,619(0,059954)	0,699(0,203857)	0,322(0,000982)	–	0,218/0,484	0,019/0,071	0,263/0,834	0,019/0,100	NA/NA	0,045/0,052	0,207/0,455	0,211/0,440
D. glomerata	0,487(0,000228)	0,379(1,55E-09)	0,446(1,72E-05)	0,392(2,91E-10)	0,401(7,91E-05)	0,543(0,000817)	0,878(0,457688	0,543(0,000817)	0,941(0,655677)	0,416(6,38E-06)	0,451(4,70E-05)	–	0,239/0,443	0,303/0,841	0,218/0,464	0,218/0,484	0,245/0,477	0,117/0,317	0,117/0,316
T. aestivum B	0,288(0,005036)	0,225(1,17E-19)	0,244(0,002085)	0,279(9,76E-17)	0,362(5,30E-07)	0,705(0,164893)	0,704(0,200989	0,705(0,164893)	0,765(0,271277)	0,295(0,001401)	0,273(0,009704)	0,541(0,001916)	–	0,239/0,812	0,030/0,120	0,019/0,071	0,032/0,048	0,212/0,420	0,225/0,405
B. sylvaticum	0,393(4,32E-08)	0,306(4,09E-10)	0,305(1,02E-14)	0,497(0,000129)	0,248(9,45E-21)	0,322(7,27E-15)	0,549(0,001035	0,322(7,27E-15)	0,555(0,001135)	0,391(4,31E-08)	0,315(6,18E-13)	0,360(2,78E-11)	0,239/0,812	–	0,276/0,721	0,263/0,834	0,272/0,738	0,297/0,703	0,300/0,743
T. aestivum A	0,001(000)	0,242(1,18E-15)	0,235(0,000429)	0,335(4,52E-12)	0,353(5,33E-08)	0,667(0,083757)	0,780(0,283665	0,667(0,083757)	0,830(0,473999)	0,412(0,011117)	0,189(0,000633)	0,471(0,000138)	0,030/0,120	0,383(9,56E-09)	–	0,019/0,100	0,048/0,096	0,207/0,460	0,212/0,445
A. tauschii	0,219(0,002016)	0,220(1,86E-19)	0,263(0,002915)	0,274(1,60E-17)	0,344(8,47E-08)	0,619(0,059954)	0,659(0,150227	0,619(0,059954)	0,699(0,203857)	0,322(0,000982)	NA/NA	0,451(4,70E-05)	0,019/0,071	0,315(6,18E-13)	0,189(0,000633	–	0,045/0,052	0,207/0,455	0,211/0,440
A. speltoides	0,577(0,139224)	0,267(1,10E-14)	0,774(0,592871)	0,351(1,08E-11)	0,376(2,32E-07)	0,590(0,030127)	0,742(0,232205	0,590(0,030127)	0,752(0,220748)	0,474(0,030474)	0,851(0,534215)	0,513(0,000507)	0,032/0,048	0,369(1,70E-09)	0,494(0,088112	0,851(0,534215	–	0,243/0,431	0,254/0,421
A. sativa 2	0,464(0,000151)	0,357(1,83E-10)	0,435(3,55E-05)	0,590(0,000762)	0,363(3,11E-05)	0,485(0,000142)	0,717(0,115983	0,500(0,000232)	0,765(0,209335)	0,396(1,27E-06)	0,455(0,000121)	0,370(7,07E-05)	0,212/0,420	0,422(1,35E-07)	0,450(9,01E-05	0,455(0,00012	0,564(0,003107	–	0,011/0,044
A. sativa 1	0,490(0,000514)	0,349(3,92E-11)	0,461(0,000128)	0,585(0,000555)	0,378(6,83E-05)	0,500(0,000232)	0,749(0,161979	0,500(0,000232)	0,774(0,209577)	0,428(8,04E-06)	0,480(0,000354)	0,371(7,52E-05)	0,225/0,405	0,404(1,21E-08)	0,476(0,000324	0,480(0,00035	0,602(0,012896	0,250(0,011066)	–

Note. Values of Ka and Ks are shown above the diagonal. The ω values are show below the diagonal followed by P-values in brackets.

Values ω >1 are highlighted in bold.

Table S4. The presence of the most well-represented transposable element families in ISs in Triticeae species.

**Species**	*Copia* families	*Gypsy* families	DNA transposons	LINEs
H. spontaneum	**13919 bp**<br>*Inga*, 84.2%, 3fr<br>*BARE1*, 12.9%, 2fr	**10921 bp**<br>*Sabrina*, 87.0%, 4fr	**3817 bp**<br>*Mutator*, 73.3%, 6fr<br>*Mariner*, 18.3%, 4fr	**2676 bp**<br>*Nadine*, 100%, 1fr
H. vulgare	**47163 bp**<br>*BARE1*, 40.6%, 7fr<br>*Maximus*, 34.2%, 6fr<br>*Inga*, 25.1%, 4fr	**12047 bp**<br>*Sabrina* 59.8%, 2fr<br>*Surya*, 39.8%, 2fr	**2742 bp**<br>*Mutator*, 98.4%, 5fr	**2676 bp**<br>*Nadine*, 100%, 1fr
T. urartu	**7570 bp**<br>*WIS*, 77.9%, 5fr<br>*Angela*, 21.8%, 1fr	**4148 bp**<br>*Sabrina*, 53.0%, 2fr<br>*Egug*, 24.1%, 1fr<br>*Latidu*, 22.9%, 1fr		**3660 bp**<br>unnamed, 100%, 2fr
T. aestivum,<br>A genome	**10395 bp**<br>*WIS*, 83.7%, 2fr<br>*Angela*, 15.9%, 2fr	**30792 bp**<br>*Fatima*, 62.0%, 3fr*<br>*Egug*, 25.7%, 2fr<br>*Sabrina*, 5.0%, 1fr<br>*Latidu*, 4.4%, 3 fr		**3660 bp**<br>unnamed, 100%, 1fr
T. aestivum,<br>B genome	**63449 bp**<br>*WIS*, 41.3%, 4fr**<br>*Barbara*, 29,3%, 2fr**<br>*Angela*, 23.9%, 3fr*	**98009 bp**<br>*Egug*, 23.9%, 6fr**<br>*Laura*, 14.3%, 1fr*<br>*Sabrina*, 13.6%, 4fr<br>*Romani*, 11.2%, 1fr*<br>*Vilma*, 10.3%, 1fr*<br>*WHAM*, 9.7%, 2 fr<br>*Lila*, 8.9%, 2fr<br>*Lisa*, 5.5%, 1fr	**20068 bp**<br>*CACTA*, 87.4%, 46fr<br>*En-Spm*, 11.0%, 4fr	**14104 bp**<br>*Karin*, 60.4%, 4fr<br>unnamed, 24.8%, 1fr<br>L1, 14.1%, 3fr
Ae. tauschii	**12272 bp**<br>*Angela*, 86.3%, 4fr<br>*WIS*, 13.5%, 1fr	**23409 bp**<br>*Sabrina*, 61.1%, 3fr*<br>*WAHM*, 36.7%, 1fr	**871 bp**<br>*Harbinger*, 76.9%, 3fr	
T. aestivum,<br>D genome	**12272 bp**<br>*Angela*, 86.3%, 4fr<br>*WIS*, 13.5%, 1fr	**23416 bp**<br>*Sabrina*, 61.1%, 3fr*<br>*WAHM*, 36.3%, 1fr	**291 bp**<br>*Harbinger*, 100%, 1fr	

Note: The total number of base pairs for given superfamily of transposable elements is shown in bold. The values after the family names are (1) the percentage abundance of the family fragments in the superfamily and (2) the numbers of the family fragments. The number of asterisks (*) represents the number of full-length copies of the element.
