# Supplementary material for: The origin and evolution of a two-component system of paralogous genes encoding the centromeric histone CENH3 in cereals
Source: BMC Plant Biol. 2021 Nov 18;21:541. doi: 10.1186/s12870-021-03264-3 (PMC8603533; doi:10.1186/s12870-021-03264-3)
Supplement: Supplementary file 2 — Additional file 2 Fig. S1. CENH3 nucleotide sequences from barley species virtually translated into proteins and aligned. Accession numbers are given in Table S1. The sequence of canonical histone Н3.3 in H. vulgare was retrieved from GenBank (accession number 769350). Fig. S2. An amino acid alignment inferred using the genomic sequences of three Brachypodium species (accession numbers are given in Table S1). Asterisks stand for stop codons in the sequences of sample 2 and sample 4. The aberrant transcript of B. stacei_1 was predicted by mapping the transcriptome of B. stacei (SRR4094449, SRR4094447, DRR090154) onto its genome using HISAT2 [75] followed by an analysis of the transcripts with StringTie [78]. Fig. S3. Experimental confirmation of the presence of a stop codon in exon 2 of βCENH3 in B. distachyon. 1. A schematic of locus LOC100830307 retrieved from GenBank. 2. A schematic of a 1776-bp transcript from the locus. Exons appear as white finger-post arrows. 3. A schematic of sequences in locus LOC100830307 homologous to βCENH3 and αCENH3 in the rye genome [25]. 4. Products of RT-PCR with primers of 1 and 2 for αCENH3 and primers 3 and 4 for βCENH3. Arrows point to the primer positions, stop codons are shown by cross. The upper scale shows the dimensions in bp. Fig. S4. A multiple alignment of the amino acid sequences of the CENH3 proteins using the Bali-Phy program [41]. BAli-Phy is software that estimates multiple sequence alignments and evolutionary trees from DNA, amino acid, or codon sequences. It uses likelihood-based evolutionary models of substitutions and insertions and deletions to place gaps. Redelings showed that BAli-Phy had 3.5 times fewer alignment errors than MUSCLE and MAFFT on simulated data [41]. For convenience, the alpha and beta forms are grouped into two separate blocks: 1–26 (αCENH3) and 27–45 (βCENH3). Amino acid residues identical in all species are shown as white letters on the black background. Amino acid motifs specific for certai [file 12870_2021_3264_MOESM2_ESM.pdf]

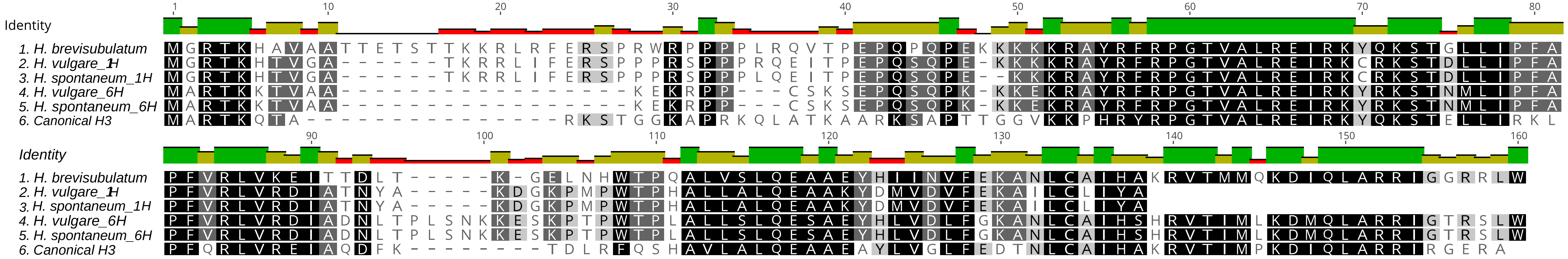

Figure S1. CENH3 nucleotide sequences from barley species virtually translated into proteins and aligned. Accession numbers are given in Table S1. The sequence of canonical histone H3.3 in *H. vulgare* was retrieved from GenBank (accession number 769350).

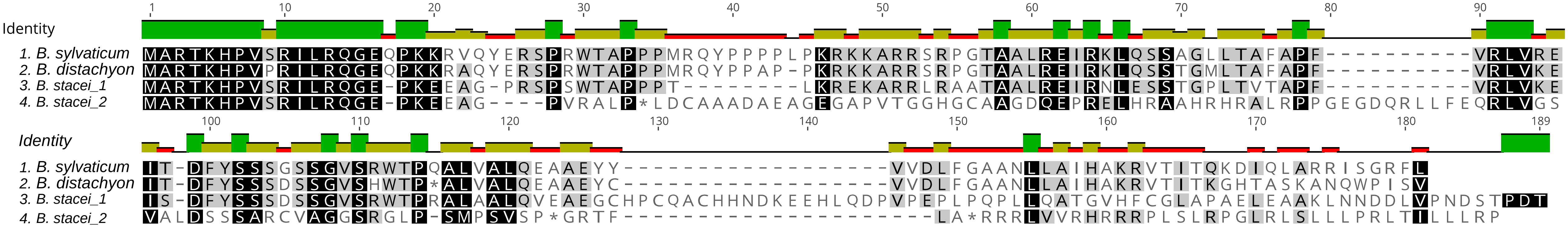

Figure S2. An amino acid alignment inferred using the genomic sequences of three *Brachypodium* species (accession numbers are given in Table S1). Asterisks stand for stop codons in the sequences of sample 2 and sample 4. The aberrant transcript of *B. stacei_1* was predicted by mapping the transcriptome of *B. stacei* (SRR4094449, SRR4094447, DRR090154) onto its genome using HISAT2 [70] followed by an analysis of the transcripts with StringTie [73].

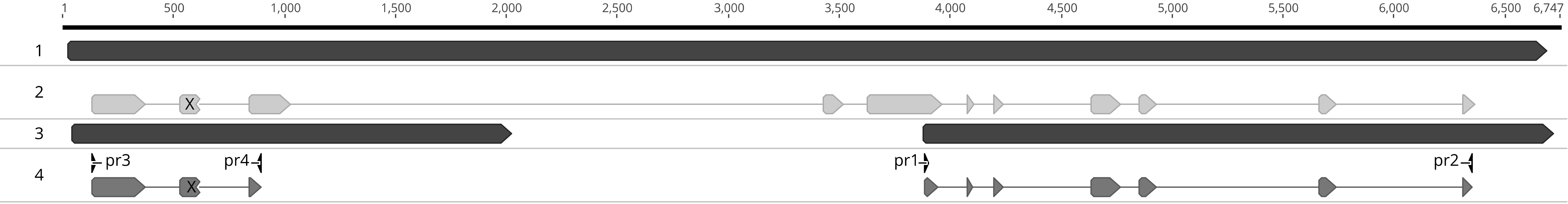

Figure S3. Experimental confirmation of the presence of a stop codon in exon 2 of βCENH3 in *B. distachyon*.
1. A schematic of locus LOC100830307 retrieved from GenBank. 2. A schematic of a 1776-bp transcript from the locus. Exons appear as white finger-post arrows. 3. A schematic of sequences in locus LOC100830307 homologous to *βCENH3* and *αCENH3* in the rye genome [25]. 4. Products of RT-PCR with primers of 1 and 2 for *αCENH3* and primers 3 and 4 for *βCENH3*. Arrows point to the primer positions, stop codons are shown by cross. The upper scale shows the dimensions in bp.

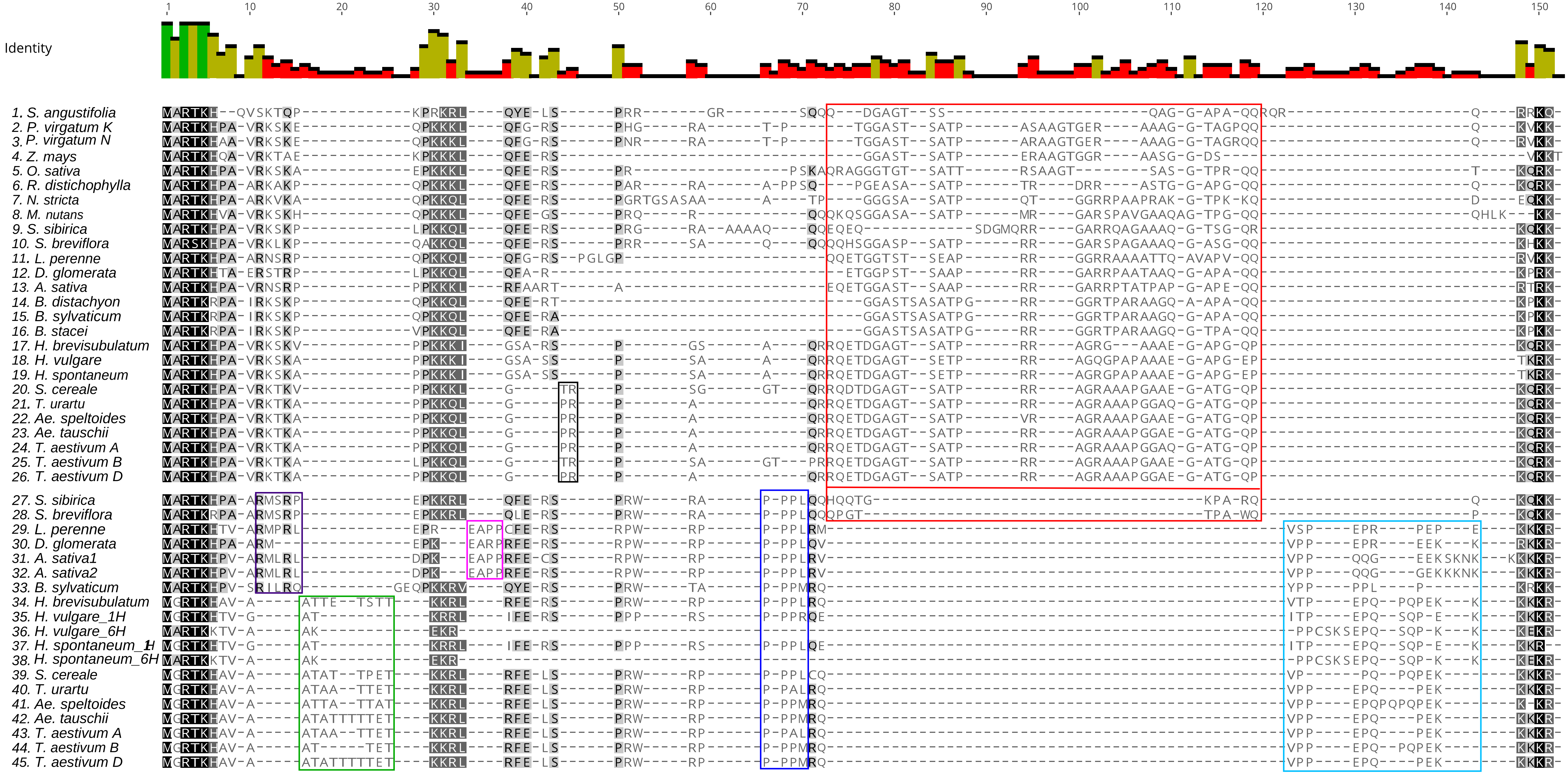

Identity
1
10
20
30
40
50
60
70
80
90
100
110
120
130
140
150
1. S. angustifolia
2. P. virgatum K
3. P. virgatum N
4. Z. mays
5. O. sativa
6. R. distichophylla
7. N. stricta
8. M. nutans
9. S. sibirica
10. S. breviflora
11. L. perenne
12. D. glomerata
13. A. sativa
14. B. distachyon
15. B. sylvaticum
16. B. stacei
17. H. brevisubulatum
18. H. vulgare
19. H. spontaneum
20. S. cereale
21. T. urartu
22. Ae. speltoides
23. Ae. tauschii
24. T. aestivum A
25. T. aestivum B
26. T. aestivum D
27. S. sibirica
28. S. breviflora
29. L. perenne
30. D. glomerata
31. A. sativa1
32. A. sativa2
33. B. sylvaticum
34. H. brevisubulatum
35. H. vulgare_1H
36. H. vulgare_6H
37. H. spontaneum_1H
38. H. spontaneum_6H
39. S. cereale
40. T. urartu
41. Ae. speltoides
42. Ae. tauschii
43. T. aestivum A
44. T. aestivum B
45. T. aestivum D

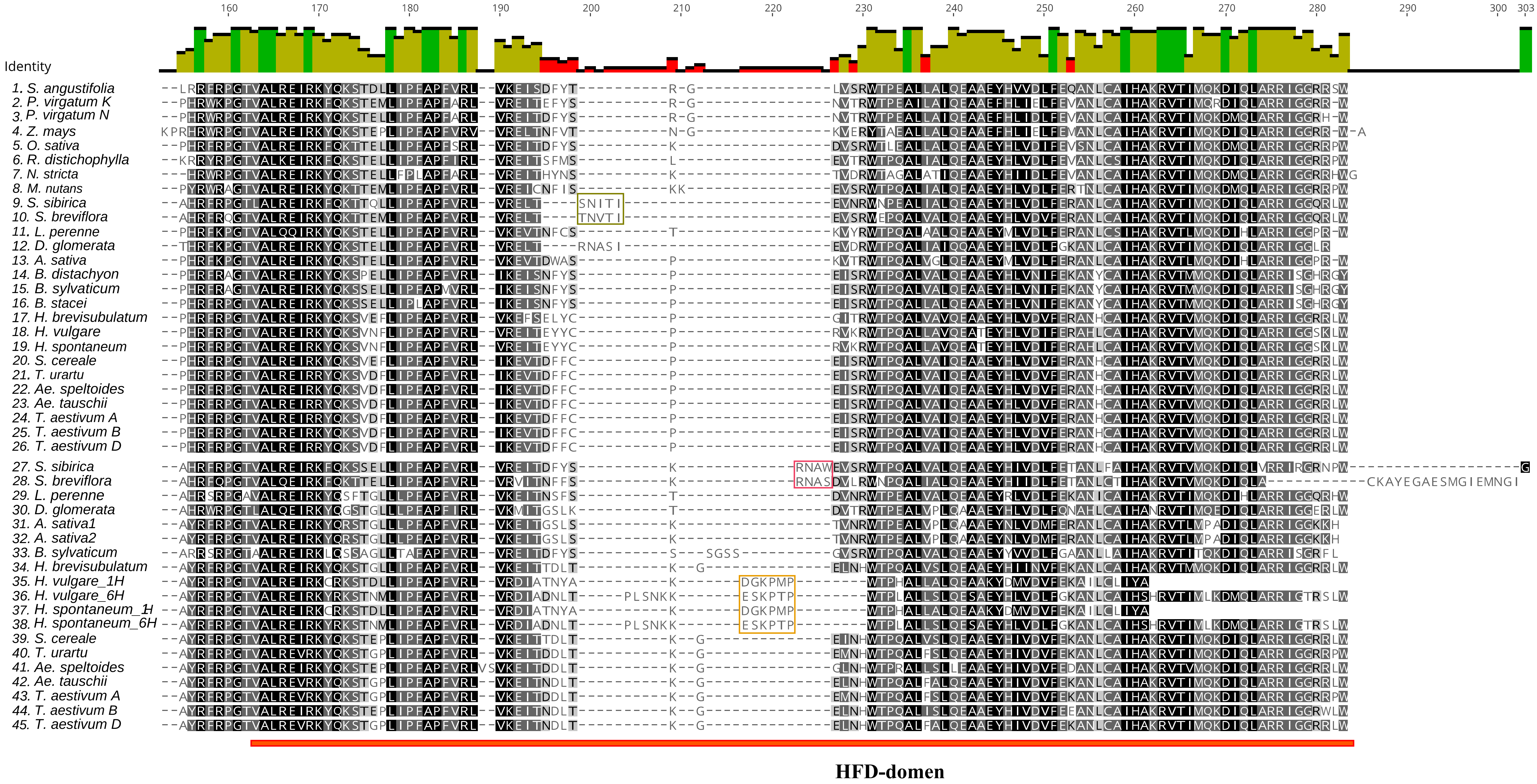

Figure S4. A multiple alignment of the amino acid sequences of the CENH3 proteins using the Bali-Phy program [35]. BAli-Phy is software that estimates multiple sequence alignments and evolutionary trees from DNA, amino acid, or codon sequences. It uses likelihood-based evolutionary models of substitutions and insertions and deletions to place gaps. Redelings showed that BAli-Phy had 3.5 times fewer alignment errors than MUSCLE and MAFFT on simulated data [35]. For convenience, the alpha and beta forms are grouped into two separate blocks: 1 – 26 (*αCENH3*) and 27 – 45 (*βCENH3*). Amino acid residues identical in all species are shown as white letters on the black background. Amino acid motifs specific for certain tribes and genera are contained within rectangular boxes with different border colors.

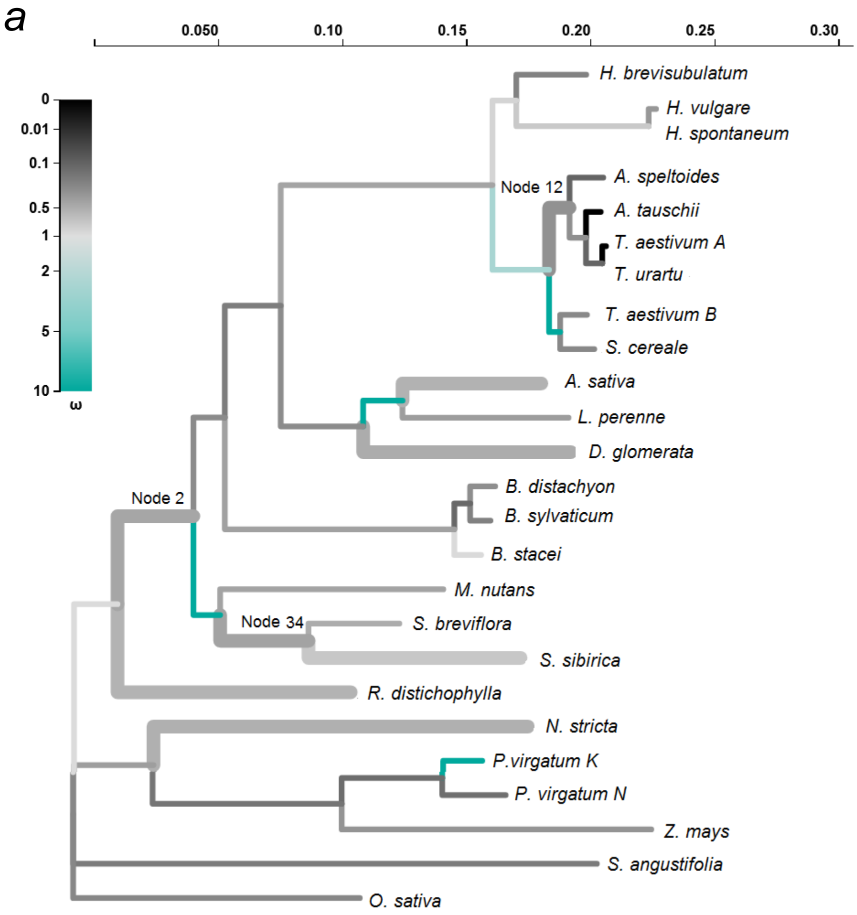

Name	Test p-value	Uncorrected p-value	ω distribution over sites
S. sibirica	**0.0000**	**0.0000**	**ω_1 = 0.385 (91%)**<br>**ω_2 = 165 (9.5%)**
**Node 12**	**0.0017**	**0.0000**	**ω_1 = 0.00 (98%)**<br>**ω_2 = 100 000 (1.9%)**
R. distichophylla	**0.0053**	**0.0001**	**ω_1 = 0.251 (90%)**<br>**ω_2 = 16.4 (10%)**
**Node 2**	**0.0059**	**0.0001**	**ω_1 = 0.00 (96%)**<br>**ω_2 = 45.1 (4.0%)**
D. glomerata	**0.0062**	**0.0001**	**ω_1 = 0.324 (94%)**<br>**ω_2 = 42.1 (6.2%)**
N. stricta	**0.0082**	**0.0002**	**ω_1 = 0.360 (84%)**<br>**ω_2 = 3850 (16%)**
A. sativa	**0.0167**	**0.0004**	**ω_1 = 0.194 (94%)**<br>**ω_2 = 29.3 (6.3%)**
**Node 34**	**0.0448**	**0.0011**	**ω_1 = 0.297 (97%)**<br>**ω_2 = 122 (3.2%)**

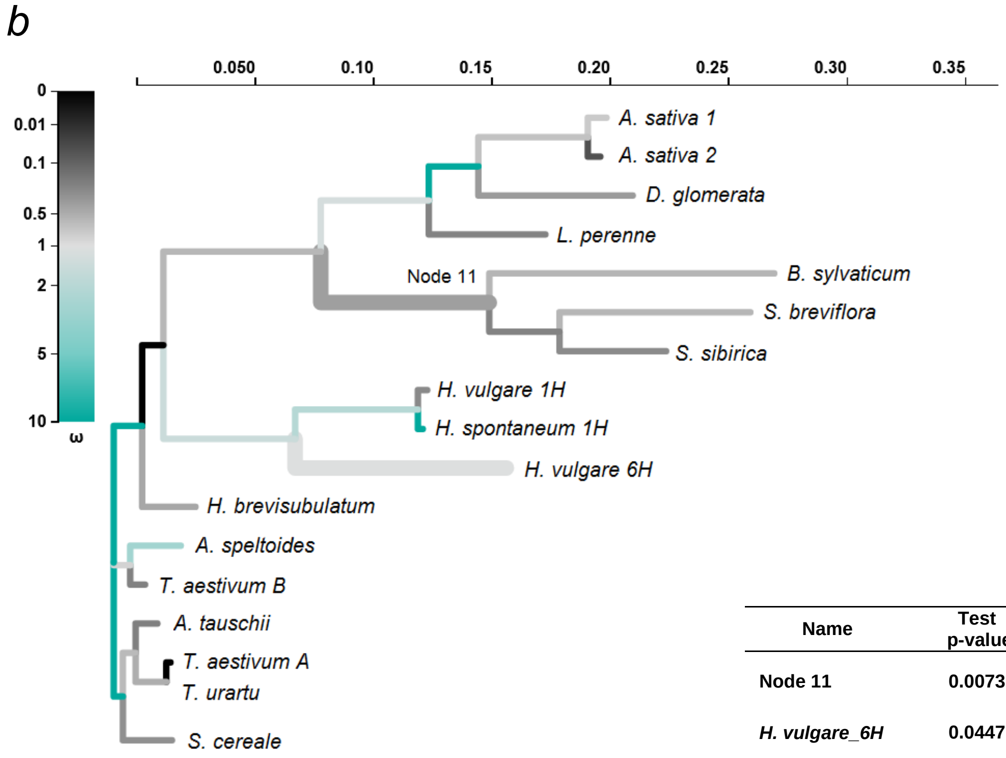

Name	Test p-value	Uncorrected p-value	ω distribution over sites
**Node 11**	**0.0073**	**0.0002**	**ω_1 = 0.174 (94%)**<br>**ω_2 = 501 (6.4%)**
H. vulgare_6H	**0.0447**	**0.0015**	**ω_1 = 0.918 (95%)**<br>**ω_2 = 169 (5.4%)**

Figure S5. Branch-site (BSREL) analysis of positive selection for the α*CENH3* (a) and β*CENH3* (b) [40]. The upper scale indicates the length of branches. Branch lengths are scaled to the expected number of substitutions per amino acid and branch colors indicate the strength of selection (dN/dS or ω). Green, positive selection ($\omega > 5$); black, purifying selection ($\omega = 0$); gray, neutral evolution ($\omega = 1$). The proportion of each color represents the fraction of the sequence undergoing the corresponding class of selection. Branch thickness reflects the amount of statistical support for evolution under episodic diversifying positive selection as determined by BSREL. The tables show the branches and nodes in which BSREL found evidence of episodic diversifying selection at $P < 0.05$.
