# Supplementary material for: The origin and evolution of a two-component system of paralogous genes encoding the centromeric histone CENH3 in cereals
Source: BMC Plant Biol. 2021 Nov 18;21:541. doi: 10.1186/s12870-021-03264-3 (PMC8603533; doi:10.1186/s12870-021-03264-3)
Supplement: Supplementary file 3 — Additional file 3 The DNA sequence of the CENH3 locus of Stipa sibirica assembled in this study. [file 12870_2021_3264_MOESM3_ESM.pdf]

**Additional file 3. The DNA sequence of the *CENH3* locus of *Stipa sibirica* assembled in this study.**

```
LOCUS       Cenh3       21134 bp    DNA     linear   UNA 08-NOV-2020
DEFINITION  Locus Cenh3 Stipa sibirica.
ACCESSION
VERSION
KEYWORDS    .
SOURCE      .
  ORGANISM  Stipa sibirica.
FEATURES             Location/Qualifiers
     gene            1187..5087
                     /standard_name="Cdpk2"
     mRNA            join(1187..1881,1981..2035,2832..2942,3041..3273,
                     3724..3931,4054..4113,4801..5056)
                     /standard_name="Cdpk2"
     repeat_region   complement(4302..4517)
                     /rpt_family="HARB-N4_ZM"
                     /standard_name="repeat region"
     mRNA            join(6716..6920,7616..7799,7913..7933,8078..8153,
                     9036..9423)
                     /standard_name="CenH3beta2"
     gene            6752..9423
                     /standard_name="CenH3beta"
     mRNA            join(6754..6891,7622..7752,7847..7933,8078..8153,
                     9036..9423)
                     /standard_name="CenH3beta"
     CDS             join(6771..6891,7622..7752,7847..7933,8078..8153,
                     9036..9091)
                     /standard_name="CenH3beta"
     repeat_region   complement(7391..7481)
                     /rpt_family="LINE1-63_SBi"
                     /standard_name="repeat region"
     repeat_region   9743..13014
                     /rpt_family="LINE1-34_SBi"
                     /standard_name="repeat region"
     repeat_region   14998..15039
                     /rpt_family="(CGC)n"
                     /standard_name="repeat region"
     gene            15005..17313
                     /standard_name="CenH3alfa"
     mRNA            join(15005..15167,15406..15456,15822..15949,16038..16112,
                     16499..16574,17165..17313)
                     /standard_name="CenH3alfa2"
     CDS             join(15041..15151,15296..15321,15417..15456,15822..15949,
                     16038..16112,16499..16574,17165..17313)
                     /standard_name="CenH3alfa0"
     CDS             join(15041..15167,15406..15456,15822..15949,16038..16112,
                     16499..16574,17165..17214)
                     /standard_name="CenH3alfa"
     mRNA            join(15041..15151,15296..15321,15417..15456,15822..15949,
                     16038..16112,16499..16574,17165..17313)
                     /standard_name="CenH3alfa"
     repeat_region   15120..15167
                     /rpt_family="(GCA)n"
                     /standard_name="repeat region"
     repeat_region   15339..15382
                     /rpt_family="(TTTG)n"
                     /standard_name="repeat region"
     mRNA            join(complement(18391..18785),complement(19988..20059),
                     complement(20272..20714))
                     /standard_name="bZip"
     gene            complement(18403..20714)
                     /standard_name="bZip"
ORIGIN
```

1 gtatgatatt ttcttctaat cttggaagtt acaagcccag tgttgtccta gcagaaacat
61 cttttctct cgaacatttg aagacagttg tgcacttcct ttcatgaacc atctttattt
121 tgttgtcaca cttttatttt tgcgatttct ttgatgaatt gtaaaagaaa tgctcatctt
181 actatattat acttgtgatg aattgacaga tgaactcaca tatgcatcgg cactagaaag
241 cactcattcc tcgtaatatt acttatgtgg tagtagtgat atagtcctcg tctccttagt
301 ccttcggttt gtttgatgct tgaactttta agtcttttgt ggtacacaac cactgtcaga
361 atctgatgaa tacaatattc agttgaatgc catctgcatt tgtattgtag tagcaatgtc
421 taaccactgt tctttgcagt ctagcatatg agtacagtac taagtcagat cagatcagct
481 gcaaaggatt gcaaagtgga cacaattgtt ctgctggagc tctctgcaac gagaaagagg
541 tacacacctg catatggtta gcccaaataa acatgaggtc tttgtctttt tacagccctc
601 catctctagt gttcagcggg tgaagaaagc tcgctgtaag ctaaaactag attaggcctc
661 accgagtgaa gaccaattca atgtagccct atagcagtag caacctggtc aacacaatta
721 tttttggcca ggcatctttt ttccatcatc ttatttctct attcttgtca tcttgccctc
781 tgcacaaaaa gaagtacttg atgagtttat tccctttgt tggttgttgc acatgtccct
841 ttcttctgtc gtcacaatct taaacaccct atcattgtgc tctctcagct ggtttgtata
901 atgatcagag ataagctgtt tatatatacg tccctttcta gtcccaacta ctgcttccac
961 tttgtgttct gctgtttccc aatcttcctt cttctcccca gagacctgca atcaatttgt
1021 tcttcctgta gtgcttacca ttttgatttc ttgtatgaaa aaggaccaat tttgagtgct
1081 tgtgccctct agtcttcttt catcagtatt tggttccttt agctattagg tttttagtcc
1141 aataaccaat tgaagttagt aattttgctg tgcaatgtcc aagactgaaa gcagaaggct
1201 gtccgacgac tatgaagttg tggatgtcct tggccgaggt ggcttctcaa tagtgagaag
1261 aggagtgagc aagtctgaag gaaagattca agttgccata aagactctca gaaggcttgg
1321 cccgacgatg acggggatgc aacaagggtc aaagggtgcc ccaagctcgg ggctccctct
1381 gtggaagcag gtatcgatct ctgatgtgtt gctgactaat gagatacttg tcatgaggag
1441 gatagtggag aatgttgcac cacatccaaa tgttatcaac ctgcatgatg tgtatgaaga
1501 tgtgcatggt gtccaccttg tccttgagtt gtgttcaggt ggtgaactgt ttgataggat
1561 aataggacgt gaccggtatt cggagtttga tgcagctgct gttattagcc agattgccag
1621 tgggttgaat gctcttcata aggcaaacat catacacaga gacttgaagc cagagaattg
1681 cctgttctcg gacagagaag ataattccac attgaagatc atggattttg gtttgagttc
1741 tgtagaagat ttcagtgacc caattgtggc gctgtttgga tcgatagatt atgtttcacc
1801 agaagcactc tcgaggcaag aggtttcagc tgctagcgat atgtggtctg ttggggtaat
1861 tctgtatatt cttttatctg ggtatgttgg gcattcccct tggcttgatt tttttcttca
1921 tagtttgcag gctttatttt gtatatccat cttttgaaag gtttaatttg tatatttcag
1981 atgcccgccg ttccatgctg caactaatcg agaaaagcag caaaggatcc tgcaagtaag
2041 tcttcctctg aactaacatc tggggaattg tttggaattt ttcttgtaat tcatatttct
2101 agcttccaac tttttagcct ttctacaacc agctatgaca atataataaa ttgaacaggt
2161 tggctcactt gtagttccgg aaggattaaa aataatttct ctgttagtat ctttgcgttt
2221 gcctatgaaa cagaacatcg tgctcattaa tgaaacattg ttgagatata cagtttactg
2281 ctgatctcaa aagacacttt gctgtatgct ctttcagtac aaattccggg tgaggcattt
2341 gattcattcg gtaatgtcag catgagtgtc aatcttctat cagctagttt ggaccaattg
2401 attgtgtggt atagaaacat acctgccaat cactgaactt tctctttttg ttagttattg
2461 gcacttgaat ttcgatttgt cttatccact gtccagttcc agtgggcaag tttccttgag
2521 atatcaaatc agtagctaat tagaatttct ttaggtgtaa acaaatattt ttcttccata
2581 cgaagccaca agagtttaac attatttact cacataaaca acaaaatatg agtatggatg
2641 tagggttaat tggagattgt gtcataagta agctgctcaa aaaggaattc aaacctttgt
2701 tttttaagaa ttaagggttc ataaaaactt gattatttca gtgtagaaac tccaattgct
2761 gaaactgttt tgcattactc agggtcttcc tctaatttct gctgtgaaac tgtttgctgt
2821 gcttcttcca gggtgaattc tgttttcagg agcatacatg gaaaacaata tcttcatcag
2881 ccaaagagtt gatttccggt cttctttctg ttgaacctta caagaggccc acagcaagtg
2941 atgtctgtct cgtgctccct tttctggatc ttcttacttg acttgatatt agtttatata
3001 actattcttg acttggggac catttataat tacaaaccag cttttgaggc atccttgggt
3061 gataggggac tgcgccaagc aagataccat agatgcagag gtcgtttcaa aactgcaaag
3121 gttcaatgct agaaggaaat tgcgggcagc agcaatagcc agcgtcctga gcagcaaagt
3181 ggcattgagg acaaaaaggc tgagaaacct tttaggaacc catgacctta cctccgagga
3241 gctagataag ctgcggcttc attttgcaca gatgtgagta tgcggccgta tccaatcttt
3301 aatttctctt tctttattac ttgaatttgg gaacatttct cagttagtga taagatgaac
3361 actgtagctg ttcaaaatgt gggtacccat atgttctctc aaaaagcaat attaaacatc
3421 aaatatgtct catttttgca agacaaatgc atgccgatta ttcctgtgtg ttgaatgaga
3481 taataatcga ttattgtgtt gcttctatca catcaaacta tgctcgtgtc agaagctagt
3541 aaaggaactc tcaaaacttc aatagttctt tttttcaaga aaccaaaact tcaatagttc
3601 cagtccctag taaaagctgc atagttctgg atgcaacatg tgctacctgc tatgtcttgt
3661 gttcaagaag gatatctgct atgttttgtt gagtactgag tgtactaagt gcttgctgtg

3721 cagatgtgca gatggagaga acgccacatt aacagaattt gagcaagtgc tgaaagcaat
3781 gaaaatggac tcactgattc ctctagctcc tcgagtgttt gatttgtttg acaacaaccg
3841 tgatggaacc gtcgacatga gggagatcct ctgcgggctc tccaacctca ggaactcacg
3901 aggtgatgat gctctccggc tttgcttcca ggtaaattca gataacacaa agccagagaa
3961 gatttgtaat gctatacgtt gcagtagcaa tatatgtaca aaattatcac tagaggttat
4021 tccacattct gacgcacgaa aacactattt cagatgtatg ataccgatcg gtcgggctgc
4081 atcagcaagg aagagctggc atcaatgctc cgggtaagga ttattccgag tgatttgttg
4141 ccccgggagc aatgtaccct cctcgatcca tcgtacattg ttgctttaag atccgcacaa
4201 atttactata gcacgtatcc attttatatg ttaaaagaca attctatgat gtctttatgt
4261 ctaaaacatg atgattccga tctagattca agtataactt agagggtgtt tgggacagct
4321 ccagctcccg gctccacgtt agatttgaag attaaaagct aaaataaagt agtttatata
4381 tctattttta atatacaatg tcaacgaaat atgacttcta accatctctg atgtacctcc
4441 agcttcagct ccacgaatct gtgaagctat gaaaacccag ctccaccaaa ttcatggagc
4501 tggagctatc ccaaacaccc ccttaatcat caatttattt tcaatgccta acatacatgt
4561 aaatagacta gtacgagcta aatatgaatt ccttcattac tatgagtaca atgttgtaca
4621 gtgtaaagac atagaatatg caaattttaa agcacgatcc aacggtggat aggagagggt
4681 atagccctcg ggttgtaggg ggcaatattt ccctattcag accacagatc aaacattatt
4741 cggcgcccgg ttcataagtt ttgacaaagc tgagatgttg ttgctgttct gatatgctag
4801 gcgttacctg aggactgcct tcccggcgac atcgcggagc cggggaagct ggacgagata
4861 ttcgaccaga tggacgccaa caacgacggc gtggtcacct tcgacgagtt caaagctgcg
4921 atgcagaagg acagctccct ccaggacgtg gtcctctcgt ccctgcgacc tgggcgatca
4981 tgcgtgcagc caaactagcc gccccctctg tctgaactct caactccccc acacccacca
5041 gccactgaaa tggcaaacca gcccccccat gtcctgttct attaactcgt aagtttatta
5101 tgatggattt tgatcatttc tgccgtaagt tacgttgtta tttgggtatg aatgtgattt
5161 atgggttcga atcttatatc ataaaattca tcaccagaat atgtttgaat aatttgatag
5221 tcggcggaaa gttggaaaga agaacgactt gtggtctgat gtttaggctc caggttcgaa
5281 attctttccc tggatatcag cctacgtaaa gtgtacgtga tttaccattt ttgaaaagaa
5341 aactttactc tgggcagtgg cagttaactg acgactctct taagacatat gtccaaagaa
5401 agttacaacg tactactgcc tcgtgattcc agcgaggcaa aaaatgtgag gccataggtt
5461 tcaacaagct tctacccaac gcgttatata aggccaccaa acatggtggc ccacttttct
5521 ctctcaacac ttttctctat gggtttctct ctttcatttt atcccctttc ccaaattccc
5581 cctctcctca tatccagcca agaccacgat catgctcatg gccatggctt ctttcgcaaa
5641 cttgatcact gtcgccgcct agcttatcgc tggccatggc cggagctcct cgccgcctac
5701 gcctagatcc gcctcatcat ggcagggtca tcttcatcgt ccacccctat ttgtccctcc
5761 cgagctcgat ctcaccgcgg agtagctcct agcgcgctcc cgcgcgaacg agcttcgaga
5821 gccacgccga gcggcagggc gtgcgcagcc tctacatggc gtttagatcg ggccgtcgac
5881 cgagctcgcc gtcttcctac accatggttc gcgctcgtcg tcttcttcca ccaaccgtcc
5941 gctaccgtgc tcgtgtgtgc ctgggtccgc caccgaggtg ctcgtccgtg gccagatccg
6001 gcgtggtgga gctcgagatc gttgttcgga gcgtgagatg gaggtcactt gccatggtgc
6061 gtgctctttt tcctccctct ccttttcttc ctctctcctc cttttccctc cacttagata
6121 ggcaggcaac ttggttcttc cgtaagcact cgtttcactc tgcaagttgg accatccgga
6181 ccgacgcggt gaggcggggc agcagtccag catggcgtgt ccaacatgct cttaagagtc
6241 tttaaaggtc gattcaagtt agcttcacat gcagtgcaca aacggtatca aaccgtcagt
6301 gaagtgtgtg accacaaatc ccatctattt atcgctttgt ctgattgttc ggttcatgtt
6361 cattgtagaa tcctgtccca ggaaaaagaa gggaaaaaaa aagcacaggt tacacaacgg
6421 ctctccgagc tgcacttcaa ttttcaccca cacctgaaat ggcacacacc gtacaagacc
6481 caattttttt gaaacattac aagcgaccca attcgaacgc agggaaaatt cgaacccgtc
6541 gcgcttctcc atgaagagcc gctccccact cactgcccac cggggcccac gcacccgatc
6601 aaacgacacg cgtcaactaa ccgttactca cgctcaccgc gaccgccgca taaaaccacg
6661 taatttggca caactcgagg gtctcattcg caaaaagagg aaaaaatatt ccccggccat
6721 tgctcgccgt cgccgcgctc cggagagagt tcagaccacg gccgcggcga atggcccgca
6781 cgaagcaccc ggcggcgagg atgtcgaggc cggagcccaa gaagcggctc cagttcgagc
6841 gctcccctcg ctggcgggcg ccgccgccgc tgcagcagca ccagcagaca ggtgagcccc
6901 ttttccgccg tggctgccct gtaagcgaac agtgcaggat agaatcgttt ggtatctcgc
6961 accaaacttc tttagaaacc acacgaccaa agaaaagatg catatatagg aatatggctg
7021 gttccttctt tgcacaaaat ccgcaagagt tcagaacaac tgtgttgatt tcatttcagc
7081 atgtgtttag aagcgtgtta gtttgaaaac agccaaagga aagtacgaat cttgggcgaa
7141 acatggaact cccaaatggc cgggatatga acagcagtaa tccgcgtgaa attagtgata
7201 gcatataggg gtgacaagca gaagaccatt gctactgtac tgccaaatca gtccatcagg
7261 ttcttcagaa agagtgacat tttcataaat gcctgtaagc tcaagccact gcaggaggaa
7321 cctagagtca cacatctcac cgagttgtgt ccagtagatc agcagttgtc ttgtcctccc
7381 atgttcatga gtataaaccc caatattgaa tgcctaagtt ggaggtacca aaccataaaa

7441 tcttcccacg acctgattct ttttaccgtt acccaaagac cacatcacag tatcacacca
7501 ttccagaaat gagaagtttg ctcagttgag gcatgggact ctcagatttg gggttgttct
7561 cttccggttc gttgtttgtt cgaagtgtga ttcactttag ggtgtgttct tggagaagca
7621 gggaagcctg cgcggcagca gaagcagaag aaggcacacc ggttccggcc aggcacggtg
7681 gcgctgaggg agatcaggaa gttccagaaa tcctccgagc tgctcatccc gtttgcaccg
7741 ttcgtgcgtc tggtgggcac ctcttgcttg ctctttatat caaagatttg gaatggaatg
7801 tgtggtgtgt aattgaaagc ctttgctgct gatgatggat ttgcaggttc gggagatcac
7861 tgacttctat tcaaagagga atgcgtggga agtgtcgcgc tggactcctc aagcgctcgt
7921 tgcattgcag gaggtgaatg ctggaacctg tcgtgttact atacctagtg gtttgattag
7981 agagcttaga ttaaaattga tgtgttactt gcgctgttaa tcactgttgt atgtatttgc
8041 ttcgaattct gatgtgattt ttcctctccc tgtgcaggct gcagagtacc acatagtaga
8101 cttatttgaa actgccaatc tcttcgccat ccatgcgaag cgtgttactg tcagtaagtt
8161 atcactgaat gcacacttct tctttctatt tgcataacta tgctaaacga aacatgccac
8221 aggttacaga tataaaaaca tttcgattac aggattcgtt tggacacttg tgctgatatc
8281 tgatgagatg tctagttttg atgttggatg aagtagcaaa atttctagtg attaatcaga
8341 agaagattta gttggtttgt gcaaacggtc attgaataat tctcaaatct caatgggacc
8401 tagtagtttt tattctcaat ggacatgcct aacaattatg gcctgtgaat ggactgagaa
8461 tttgctaatg ctgaagaatt tagttctgct tcgtttattt atttattttg aaccggagtc
8521 caaaggcctc aatccattaa ttaagtagaa gataattgcc cagttaatta gtggaaaacc
8581 cggctaaaac cgatacaaaa agctcacaat gccatcacct taccgcacat gagctccacc
8641 ctcgtttaga tatcaaagct tgtcacgata gcagaaaatt caaggatcta agtatcattt
8701 agctgtatat ttcttatctc agggctagac aaatggtatc aaatcagaga aattttgaaa
8761 cttggcatat atgtgctgtt ctttattctc cttaaagctt tgcaacatct agaatgcatt
8821 ggaaacataa tcgttgttgt gatttgtacc ataacaaata cagtagatgt tttgatttag
8881 agatgacata atatatatct gaaaaggatg ctcctgaaca aacaaacgaa ctgctttatt
8941 atgaaataat ttgttagaat aaggttagga aaatacatta gcagtttagc ctgttctaat
9001 tctaatgacg aatgattatt tttttgttgc tgcagtgcaa aaggacatac agctcgtaag
9061 gcgtatcagg gggcggaatc catggggata gagaggaacg gcatttaggg actaatcaaa
9121 gatgcttttc tgaacctgaa gcacgatgaa cggcatgata tctgaacagt tggtcttaac
9181 atcttaggaa ttagtgtagg ttatcgtcaa tcagggagct tgtcgtcagt tcattctgat
9241 ttggtagatg ttagaattcg tttgaacctt cagttttatg cctaacataa ttggtctagc
9301 tcatagtttc tgcaaacaac ccaaggatat gggttttaaa atgttgactt gaaatcatcg
9361 cgtatttttc atgttaattg catggaattt ctctagaacg tagttcccgc tggtatagct
9421 gactgacctt ggggaaataa ttggagggta tttttgagtg tcctgtcatt gtgaatgttg
9481 gttcatacat catcatgctt gattgaagaa ccctgtgctc aacgaactgc acccaaggcg
9541 gtcttgtgtt tccaaattat cggttgagtg ggcagtagat tcagattaat ttgtagaaga
9601 atatgcagtg aaagtagcct cctgtctgaa tttctttccc cctgccaggt acagaaaata
9661 caaacaagca ctcacgccat gccttaactc gtatacattt tatttctgga gtttcgatcg
9721 cgagattgtc tctgctggcc agttctatgt gaaggcttgt ttacatgata agttgaagca
9781 ttttcggtgg actttagttt ttgtatatgg ggctgcacat gaggatcaaa aggaagtttt
9841 tttgactgag tttgcctaga tttgtgctga tcagaccaca cctttgtgtt agtgggtggg
9901 gatttcaata tcttgaggtt tgcttcagaa aagaataaat ctatgaggca tcataggagt
9961 caggatatgt ttaatttagt cattagttta tataagatga gggagttggt tatgtcgggg
10021 ggaaaattta catggagtag tcatcaatcc tcccctactt tggagaaatt agatagaatt
10081 ttggtttctc aaggttggga ggggcacttt cccttagcta gtgtgagtaa agttccaagg
10141 tgtttatcaa accataatcc tttattgatt tcttttggtg agactgaggt taaaagactt
10201 aaagattttt gctttgattt ggcttggctg aaacgtcctg atttttttga tagagtccaa
10261 cagtcctggt gtgtcctcac taagtgcaag gggaacttgg actgtttcct gacaaagtta
10321 aaacggttga aacaatctct aaaagggtgg ggttctaatt tgaggggtga tcagattaag
10381 aggagaaaag atattcttct tgaattgcag gatcttgaat tagaagaaga attagttggg
10441 cttaggaggg agggcctggc tagaagggct gccctggaat ctgaactgtt gtctagttat
10501 gatgaggagt tattttggtc tcaaatatca agtgagaatt ggctgttaaa aggggataac
10561 aatattgcat atttccatag gattgctaat gggaggaaaa gaaagaatac catttttagc
10621 ctcatggatg gtgaggaagt tatcgagggc gatgaaaatt tgttggctta tgacactagt
10681 tattataaat ctttgtttgg tccttctgag ggtaataggt ttcaattggt agacaattta
10741 tgggatcctt ctgagcaggc tactgcagag gaaaatgagt ccttatgtag gccttttgag
10801 gaagaggagg tgaggcaggt tgttctttcg atggagaaaa ataaagtagt tggagtggat
10861 gggttcccaa ttgaatttta tcaacattgt tgggatatag ttaagattga tttgatgctt
10921 ttatttcaag atttgcatgc tcataatctt gatctttatc gtatgaacta tggaattatt
10981 actcttttag ctaagactca ggatgctgat gtgatacaaa agtatagacc tatttgtcta
11041 ttgcgggttt gttttaaatt tttttctaag gtgttatctg ttcgtctaga tctggtcttg
11101 catcgtataa ttcagcagtg tcaaaatgct ttcatcaagg ggagaaatat tatggatggg

11161 gttctttctt tacatgagat tttacatgaa acaaagagaa ataagcaata gggtatagtg
11221 ttgaagttag attttggaaa agcatatgat aaggtggagt ggaattttct ttttaattgc
11281 ttggagttac gaggtttcca ttctacttgg tgtagatgga ttaaggctgt ggtgaagcaa
11341 tgtactctca atgtgaaagt taatggggtg acaggtccta attttgatag tttcaaggga
11401 gttaggcaag gggatcctct ttccccсttg ctgtttaatg tggtagctga cagtttggca
11461 aaaatggtac aacaggctca aagtaatggt ttacttactg gtttggtgtc tcacctgatc
11521 cccaatggag tggctatact acaatacgta gacgatacta ttttgtttat tcaagatgat
11581 ctggaagcta ctagaaattt gaaacttcta ttatatttgt ttgaagggat ggctgggttg
11641 aaaatcaact tttaaaaaag tgagctcttg atggttttgg aggatgctga gaaggctcag
11701 gtgtgttcaa ttgccaaata gggtcttggc ctattaagta cttgggtgta cttgtttgtg
11761 gttgcaagtt acatgttagt gattggaaaa gtctaaaggg caagcttaag aaaaggttgg
11821 atggttggca agggggagcc ttgtctttag gaggtaggag aactcttatt aatacttgtt
11881 tgacaagtac tcctatctac catatgtcta tgtttcgttt acctaaaaca gtactatcca
11941 atatggataa aactagacga atgttctttt ggcgggggga gggccaagaa aagaaaattt
12001 caaatggtaa aatggaagct tatttgtcgt cctaaaaact aagggggttt aggtattaag
12061 gatctggagg tcatgaacat tagtctactt tgcaagtggt ggtggcggct agagcatgat
12121 caaggcttat ggcaaacttt ggttaaaaag aaatatgttg gtaacaaacc tattgttttt
12181 tataaacgca aacaatctga ttctgcttgt tggtatgacc ttttataaat aagagaattt
12241 tatttgttga ataggatgat gttattgggt aatggggcta gggtgagttt ttaggaggac
12301 acttgtgtgg ttctacctcc ttgagaaggt aatttcctac tctgtttaat atttgtgtca
12361 acaaataatg cactgtaact aaaattgcta ggagacaatg acaactgtct cttaagagat
12421 ggcttaatga ggatttgtat tgcaagtatg agaaattgag ggatatgttg gctagagtaa
12481 tcttgaatgc tgaggaagat agacctcact ggagagcctc ttcatctggt cggttcactg
12541 tcaaatctgt gtatacacag ctagctgatg tttggcataa tcgttctttt aagcatttat
12601 ggaaagctaa agtgcctcat aaaattcaga tttggttatg gatgatatgg cacaatgcta
12661 ttgccacaaa agatatatgt tacatagagg gtggtatgtg gatgaaaagt gtagcttttg
12721 tgaagcacca gaaactatac accacttgtt ttttcaatgt ctagcagcta aatatatgtg
12781 gagcaaaatt agcattgcta ttggggcttc ttgtcgtcca ggcaactttg gtcaatattt
12841 taattggatt ggacaattta tacctgctag tgcaaacatt caaattgttg gtttggcggc
12901 atgttgttgg gctttgtaga agttgaggaa cagagcttgt ttgaggagga caaattaatt
12961 aaatgcccag cggagattgt ttgttatgcc tactcattta tcaagtattg ggcacagctt
13021 caggcattaa gggacaaagt tgtgcttcaa cacggtgcag cggcgctaca ggcggtggcg
13081 ttagcccatc atgaagctgc tgaggatgca tctgcttcga tagagactct ggcttctcaa
13141 ccgttgctgg atgcacctga tgtgtaggtt tcaatgagcg tttggctatc tcatgtgttt
13201 gtattttggg tagatggttg ttgtatcaac cactgatggc tttgaattga tggtttggtg
13261 tagcttgtgt ttagaagaat gttggactag ttagggtgtt acatgtctgg ttgttactct
13321 gaacatactc tgactctgta gttccgttat ataaatagtg gaacccgggg gtgcttcccg
13381 tatggaaaaa aaagattcag gtactgcttc tgcatggcat gggtgtgaag gtgatctcgt
13441 ttggaagttg gaacctttgc tcctaacaca tgcatgcgtc gcgtggtagc gtggtctgca
13501 cctcctagca agttcggtgt ccaccgtcca tgccagtgtc agatctgtgc atgttttcgg
13561 tacaggtttg tgcgtgtgca tatcttacgg aaacttgtca cataaaagaa tcgccatgtg
13621 tgttcccaac gataacataa acttgtcacc aggggaatcg aagatgtttg aataactcta
13681 tacttagcga aaagttgaca acaatgagtt ttgccccatc gtcaggtgtc tagtgatccc
13741 ctaaaattga tacgcaccga agagagccgc ctctgcaaat cttctgccgc taaatttcat
13801 cggacattaa acaaacaaag gcccatgtag cgtcaatttt tcgattctct acgaacttca
13861 cacgaaacgg tgcgtaaccg agagtagttt accattttat gcacttgtac gtgtaaaact
13921 ggcccctttg atttttcctc gatttaaaga ttctaaaaaa aaaattttac tttacatcct
13981 tgaattgttg ggagagtata aaaaacacct tgaacttcta aacggtgcat tttatcctcc
14041 cttccccctg ctagttaaca aggttttgac catctaatac cactatgtta acatttaggc
14101 caacgtcaac aaccagctac caaatcaatt gaaacaacac gctctaattg ttctctttcg
14161 tagcaccctc tgatgatttt agctctatgt tctttcttct cagtccacgg acagtgtctc
14221 gaagatcaaa taaaatctga tttacaaata cgttattctc tcccttgaac caagcaaaga
14281 agctgcaacc tgcttcctac aaattcaaac gaaattttac aaaattgaga acatttgtgc
14341 taaacaatcg aacaataact gaaatctcaa cccaaatcaa ctcactcgag ctttagcaca
14401 tttgatgttc cgccttttct agaggagggg ggcaacgaga gaggaaagag agaggtggct
14461 acatgacagg cttagatgat caaaatcatg tttgactagc tagggatgtc ctgtgcctgg
14521 tttagatagc taaggaataa aatgcacctt tagaaattca ggatattttt taggatattt
14581 tttagacttt cgtaaaaatt caggaggaag atagacatct ttcttctcga aatctcaaga
14641 gaattgtgaa ggataccaat cgagtcggat cggaatcggc aagttccgtc ccgacccgtc
14701 gcgcttcgcc cggaacatgg ctcgggtcgg ttcgtttcga ccccctcacg agtcacgatc
14761 cgcgacaatt tccattcgaa cacatcgcgc gtctctcctg gaaccgctcc cctccagttt
14821 cccatctcgc accgccgcaa cagcacacac accggtggac acgtcccact gccaacccgg

14881 gcccacgcgc cccaacgttc ggctcgcgtt ggcatagcaa ccgctgctca cacaacacaa
14941 ctcacctcac cgcgggataa attgaaacga cccgagggtc tgattcgcaa aaatcctcgc
15001 cgagtcgccg tcgccgccgc cgctccccgc cgcggacgcg atggctcgca cgaagcaccc
15061 ggcagtgagg aagtcgaagc cgctgcccaa gaagcagctc cagttcgagc gctcccctcg
15121 cggcagggcg gcggcggcgg cgcagcagca ggagcaggag cagtcaggtg agtctccctc
15181 ccccctcccg cttgccttat gagcgaatgc cgccgccgcc gcttcgttgg gcgcgattcc
15241 ccgcccgtga tgctgagttc ttgctctctt tctgtgtatt tccttttgct tgcaggtggc
15301 gcgagcgcct ccgcgacccc ggtgcgtgcg ggcgcaaatt tgtttgggtt gttttcgttt
15361 gtttgtcttt tttttttgt ttctgacttg cgcgtcacct gtttgatgga atgcagagga
15421 gaggtgcgcg gcgccaggcc ggagcagcgg ctcaaggtgc ggtgttcttt gggttttgag
15481 ggtttcccgg cgcgtgttta ggccgcgccc taggggttgt ccgcgcgttg cttggggttt
15541 ttcgccttgt tcttttttc tctatgattc cccttcgtta tgcggacatt tcttcggaat
15601 gttactcaag gagcaacctt tatgcttctt ccgatcttgt tctttgcccg taggggtgga
15661 gtggatcgac agtttttctt tacttggtgc aatttttagt ttatcttgta gcaagatgat
15721 ggcaaagtgg acatggaact atcagttttg gagttgctct cttctggtta gttattcttt
15781 ccattggtga ttgactctag tatgtattct tggagaagca gggacatctg ggcaacggaa
15841 gcagaagaag gcacaccgat tccggccagg cacgttggcg ctgagggaga tcaggaagtt
15901 ccagaagacc acgcagctgc tcatcccatt tgcaccgttt gtccgtctgg tgggtacctc
15961 tggcttgccc ggctctattt caaagatttg gaatgtgaag tgtgtgatgg gagcttttgc
16021 tgctgatgaa tttgcaggtt agggagctca ctagtaatat taccattgaa gtcaaccgct
16081 ggaatcctga agcgctcatt gcattgcaag aggtcaatcc tgaaatctct cttgttattg
16141 tacctgatgg cttgattagg gaactgtaat gtgcatgaat taaagatccg aagactgtca
16201 gttattggat agataaaatt tcaaaataat tttcctaaat tttctgtatt gattccactg
16261 ccatatttat cagcacttat gtaggaaggg aatgaaggtg tctcagtata gcttttgttt
16321 gtagcaatat gcttgttatt gctgaataat gaagactgaa atgaggcgtc gagaagaaca
16381 tagaactgac tatatttctt gtgacctatg aaataagtta atgtgttaat tgctctttca
16441 gtcattgtcc tgcatatttg cttcaaattc tcatgtgatt tttcttctca ctctgcaggc
16501 agcagagtac cacttggtgg acttatttga aagggcaaat ctgtgcgcca ttcatgcaaa
16561 gcgtgttacc attagtaagt gttcatcgaa tgaacccatt tgtttctcca gttataaaag
16621 catttcagtc gcatgattat ttttaacact tgcgcatata tttgatgtga tatctagttt
16681 tgatgttgtt tgaaatggca aattttctat tgatcaatga taagaacatt tatagtttgg
16741 tttgtgcaag ctacaattct caaatctcat ttaaggcttg tgaatggacc ggaattcgct
16801 aatgctgaag aatatagttc cagttcattg agataccaaa gttggttaat gtagcagagt
16861 aattcaagga tctaggtttt gatttacctg tttatgtctt atcttagaga cagaaacatt
16921 gtatcaaatc agagaatttt gcaataggaa aatatgctat tgttccttaa agcttggcaa
16981 cacctagaag gcattaggaa catcaccatt gttgttatct gtatcgtaag aaatagatgt
17041 ataatgtttt gatttcagcc atgacttcat gtacgtcatg gacaaacgga actgttttat
17101 gaagaaaata catggataga ttagcacgtt cccattctcc tgacattttt tgttgttgct
17161 gcagtgcaaa aggacataca gctcgcgagg cgtattgggg ggcaaaggct gtggtgatac
17221 taattgaaga tgttgttgtg aatcctgcag catgatgtat cactgctctt aacatcttag
17281 gaactatgct aggtgtatct tcatttaggg agcttatctc cttgttaaat ctgatttgat
17341 gatacatgtt aggagtcgtt tgatttttca gcatctgctc cattttaatt ggtctaattc
17401 agagtttctg cagacagccc agtgatatga attgtgaaat gtaaacttga aattgttgag
17461 gaattgtggt actagagcaa aagaaagagg ggaaccattt tccctagcta ttcactgttc
17521 atctaatctg tccgagcata tgctgatgcc attggtaatt ctaattcaaa atttctactg
17581 gcagaaaaga ccaacgaaga attgcatcta cttgtgtttg aaaatattta ccctaattct
17641 gctttggatc cgacgatctg cgtcgcactt ctcagctttt ttggaccagg caatgcagaa
17701 gtatcagagc tgaggaagcg agcacaaatg gagatggatg ctaggtagga taggcacctc
17761 ttaagtgctg atggtgagag gaaggaagat agtgaagtga aatcccactt acgaatgtga
17821 agctcttgaa gctttccatg tgtcggagtg cactagtagc tggggtgctg agtggtcagg
17881 attcaagagc gcggcaccat gagtcctaag catggcatgc gatgtgttgc tgtgtttttc
17941 ctgagttata gtgaaactgt tttacccaac tatgatgtgg atcatggcca taactttgat
18001 gtatatttca gtatctagtc catatccagt attttccgca acatttcaaa aatattccat
18061 attttccaca aatgtgtatt tgtaaattct gtcgatgacc tccatttttt ctagattatt
18121 tcccctgcct gaaattgaca tgaactttag gctattgaca aggcatcctc gcagtttcaa
18181 tcacaagtca gagaggtgtg tagttcaccg cgacaggtca cttcaactgg ccaccagcac
18241 ctggggctca aatctcccca agttgtagtg atgatcacga gcgtcatgac gcagatagga
18301 tgcacgaatg cacagatcag atgtgcctga gaattggctg tcgatttctc taaagaaata
18361 tcttatccta aacagaggta gtgcaggtgt tcactaggtc aaacccaatg gcactctcta
18421 gtctccacgg agcagtaacg tgaccatgat taaaccacgt cgggcataac ttatcatgta
18481 caaggaagca gctctgataa atcaaagatg tgcctggcag atttacgtac tgagaaagcc
18541 tcggaaaaga tcagctgaga caagctaggg ctgcttctgt tctacaaact atgagccacc

```
18601 gggctgggac aagctagaac ctcaagctaa caactgtttc tactgcaaac acacaaagta
18661 tataagcggt gtaatctaca aaattagtat ggacatcatc atcgtcaaag aggggaagaa
18721 cttgttctcc ggagttgata cttgggttct gggggagggg cagagagcag tatcttgtcc
18781 aacttctgaa aatttacaaa actaatatcg ttagagagat cagacatgac atgaatatga
18841 tgtaataaga tgtcctaagc taagtattgt ttacgcagtc tcaagaacaa gaatcaatta
18901 cttcatgcca acaaaagttt atgcatcaac agaataggag tattgtaatc tcattgtgtt
18961 aggccaaaca aaaaatggac taagatcata gccttataaa aatatgaact tacattacat
19021 ggaaggatat gattacaaat atcctacaat gtttgcttta catatctcac cagaatcagt
19081 tctaagtgat ctgatgtgtt tactagataa atgagcccca cctattcgaa ggataaaatg
19141 aaactggacc caaaatacag aaacacaagc ataggtgctc ggtggctttg cacataatta
19201 tgatttaatg agcagtaatt accaatgtaa cgatttctcc aagtaatctg tttgtcgggt
19261 gtggttcatg ccaacacatg aaatgtacat tttatgaagg atttgtttgg cactttaaaa
19321 tatgtgaagg actggaacct ggattgatgg ctacttccag gattgtcaat tgcataaagg
19381 actgagactt tcattattta aatcgtatca tggactactg gataccataa aaattctaac
19441 caggtgacaa aagtcaccgg gcaatcaatt ctattatgct aagtacagag acatgattcg
19501 aataaaaatt cggcaagata acccatgtat acatattcaa taatcaggaa taatgttctc
19561 gacagagcaa agcaactaag gtaagggcaa taagcatctc agtgaattac tacgcaacca
19621 gagattgtcc ataaagttct accagtcaat ggagtagttc ctcctgcaaa ctgcttatta
19681 aaatttaaaa atagcatgca ccgctgagaa atcactagca tgcttaataa ataagaatca
19741 accaaaacga cggactcaaa catatgtgct ttcaacaatt aaaatatatg caacatatca
19801 aatccaccct gtaaaacatt gaaatactgg caaaataatg atgatgttat tagaatatta
19861 catacttagt tcactatcaa tataaagtgg aatgctttag ttgaaagtgg aatgctttag
19921 ttgaataccc tccaccaaaa agaaggggaa aagataaaca gtattaggat aacaggagat
19981 aaagtacctt ctgccccttt agcctctcgt tctcctcttc tagacgagat accttgtttt
20041 caagctcgtt agtgtaagcc tggcaagcat acaaggaaaa taagttacgc caacggcata
20101 gcagctatga tatgcttgca tttctagatt aaacagtgta aaataaaatg tcaaaaccaa
20161 tacattcgaa gataaattat cgttaagctc tgatgttcaa accagaacat tataacagga
20221 cagaaatcaa atacattgaa atcagatata tacaggacaa gagaaagaga cctgtttcct
20281 cgctcttgat cgtgcagctg attccctatt ttttatcatt ctcttctgcc ttctttctac
20341 caccttatca ggtacacccc ctgaagcacc acgcttgcgc ccaggtgtct gaggatcaga
20401 aagtgcaccg agcattggtg aagaactctg gccatccgag taaatcgatt ccaggatagc
20461 acctgttccg acagtcaatg gctgagggcc caactgactt gccatgtaag aacctcccat
20521 gcttggatgc tggggctcca aagtttgctg ctggtactga tgtaaccact gtgctccggc
20581 gttcaaattg ggcgcaccag ccacaataac actgctccca actgcatcaa tgttggctgg
20641 caactcattc gaatccttca ggtacccttc tgtgacaacc ccagctttga ccaggaaatc
20701 ctcaagtgtc atctcgccaa gtgtcggctg cctctcctgt ctgcgccggc caccctcagc
20761 aattcccttc ggtgcatcct ggatgccctt ccacaactca tccacggtct tcttgctcag
20821 ctccggaggc acggtgatgc tcccctggcg ctgcaggccc gaggccggtg cagactggct
20881 cgtgacgccg ccaccatcgg ggtccacgcc gtcaggaaac acgctcttga gcagctcgtc
20941 gaggttcatg ctaagcaagg gctcccccaa atggttctgc acctcatcga gggtcaggct
21001 atacagggac ccttgcctcg ccaggccctg catctgcccg cgctgcgccg agccagcgta
21061 gctaccgccg gtgccggtgc tggtgctgcc gcctccagcc tgcgacgcca ttgcctgaat
21121 catcaaccgg cact
//
```
