# Supplementary material for: The origin and evolution of a two-component system of paralogous genes encoding the centromeric histone CENH3 in cereals
Source: BMC Plant Biol. 2021 Nov 18;21:541. doi: 10.1186/s12870-021-03264-3 (PMC8603533; doi:10.1186/s12870-021-03264-3)
Supplement: Supplementary file 4 — Additional file 4 The DNA sequence of the CENH3 locus of Lolium perenne assembled in this study. [file 12870_2021_3264_MOESM4_ESM.pdf]

Additional file 4. The DNA sequence of the *CENH3* locus of *Lolium perenne* assembled in this study.

```
LOCUS       CenH3         20826 bp    DNA     linear   UNA 30-DEC-2019
DEFINITION  Locus CenH3 Lolium perenne
ACCESSION   .
VERSION     .
KEYWORDS    .
SOURCE      .
  ORGANISM  Lolium perenne.
FEATURES             Location/Qualifiers
     gene            5..3497
                     /standard_name="Cdpk2"
     mRNA            join(36..974,1057..1111,1857..1967,2056..2288,2528..2735,
                     2891..2950,3052..3497)
                     /standard_name="Cdpk2"
     mRNA            join(3876..4286,4369..4443,4901..4976,5571..5767)
                     /standard_name="CenH3beta"
     gene            3969..5866
                     /standard_name="Cenh3beta"
     CDS             join(4035..4286,4369..4443,4901..4976,5571..5620)
                     /standard_name="CenH3beta"
     repeat_region   5868..5908
                     /rpt_family="(TAT)n"
                     /standard_name="repeat region"
     repeat_region   6620..6936
                     /rpt_family="LINE1-62_SBi"
                     /standard_name="repeat region"
     repeat_region   7601..7663
                     /rpt_family="LINE1-62_SBi"
                     /standard_name="repeat region"
     repeat_region   complement(10388..10458)
                     /rpt_family="TREP1311"
                     /standard_name="repeat region"
     repeat_region   complement(10633..10715)
                     /rpt_family="LINE1-73_SBi"
                     /standard_name="repeat region"
     repeat_region   complement(10746..11469)
                     /rpt_family="LINE1-59_SBi"
                     /standard_name="repeat region"
     gene            11903..14741
                     /standard_name="CenH3alfa"
     mRNA            join(11919..12085,12172..12197,12285..12324,12667..12797,
                     12883..12957,13853..13928,14540..14705)
                     /standard_name="CenH3alfa"
     CDS             join(11983..12085,12172..12197,12285..12324,12667..12797,
                     12883..12957,13853..13928,14540..14586)
                     /standard_name="CenH3alfa"
     misc_feature    join(13928,16714)
                     /note="Geneious type: Junction"
                     /standard_name="junction 17"
     repeat_region   14740..14836
                     /rpt_family="TREP1084"
                     /standard_name="repeat region"
     repeat_region   complement(15176..15275)
                     /rpt_family="TREP73"
                     /standard_name="repeat region"
     repeat_region   15582..15694
                     /rpt_family="TREP1069"
                     /standard_name="repeat region"
     gene            complement(17630..20767)
                     /standard_name="bZip"
     mRNA            join(complement(20485..20742),complement(19340..20362),
                     complement(19040..19111),complement(17695..18010))
                     /standard_name="bZip"
```

```
     repeat_region   20672..20704
                     /rpt_family="G-rich"
                     /standard_name="repeat region"
ORIGIN
        1 ctgtctgttc ccaactactg tttccagttt gtgctctgct gttccccaat cttttttttc
       61 cccagagacc tgcgatcaat ttgttcttcc tgtatcagtg atcactgttc tgatttcttg
      121 tgtgaaacag gtcgatttgt accggtttat tgctgatttt gggtgctcgt gccctcttgg
      181 cttccttctt accggtattt gttaccttca gctgttaggt tcttgagtca aacaactggt
      241 tggagctact cttgctgcta cgcaatgtcc acgactgaaa gcagaaagct gtctgatgac
      301 tatgaagttg tggatgtcct cggccgaggt ggtttctcga tagtgagaag aggagtcagc
      361 aagtctgaag gaaacaccca ggtcgcgata aagaccctcc gaaggctcgg cccagcgatg
      421 atggggatgc agcaagggtc aaagggtggc gcaccgagct cgggcctccc gatgtggaag
      481 caggtatcca tctccgacgc gttgctgact aatgagatac tcgtcatgag gaggatagtt
      541 gagaatgttg cgccccatcc gaatgttatc agcctgcatg atgtgtatga ggatgtgaac
      601 ggcgtgcacc ttatccttga gctgtgctct ggtggtgagc ttttcgacag gataataggc
      661 cgcgagcggt actcggagtt tgatgcggct gctatcatta gccagatcgc cggtgggttg
      721 aaagctcttc acaaggcgaa catcatacac agagacttga agccggaaaa ttgcctgttc
      781 acggacagaa aagaagattc cacgttgaag atcatggatt ttgggttgag ttctgtagaa
      841 gatttcagtg acccgattgt ggcgttgttt gggtcgatag attatgtttc accagaagca
      901 ctatcgaggc aagaggtttc agctgcaagt gatatgtggt ctgttggggt gattctgtat
      961 attctcttat ctgggtacgt tggccatcct ttcttctgat aattctcttg gcttgactca
     1021 tatatcatag ttcacaggct ttattgtaca ttgcagatgc cctccatttc atgctccaac
     1081 taatctagaa aagcatcaaa ggatcctcca agtaagtatc catctgaact agcatctggg
     1141 gaaaaatttc tgcaatggtt atattattgg tttggagttt tcttgtgatt catatctcca
     1201 gtttttcacc tatacagaac ttctacaacc tagtatgaca atacctgtag tggaaaaggg
     1261 tgttaaataa atttctttga atgcatttgc ttatgaaaca agattgttgt gcccatgaat
     1321 catctgatct ccataaactg tatgccctat gctcctatgc tcttccagta taaattatgg
     1381 gcgagccatg taatgtcaga atgagtgtcc accttgtgtc agttggattg gaacaattga
     1441 ctgtacttag tatagaaaca tgcctgtcaa tcattgctga agtttcttct ctgttagtta
     1501 ttgccacttg aatttttatt tgtcttatct gatgtctagt tccaaacttc caatggacaa
     1561 gtttctttga gatatttaag tcagtagcta actagagatt cctttaagga tgcaaacaaa
     1621 tactgtctta catgtaatcc acaataatat aacgtgttct actactcaga ataacaccca
     1681 aaactgtgag ccatcatgga tgtggggtta attgactttg tcataagcca ctcaaaaagg
     1741 aattggtacc ttttctttga agaattaagg ccttataaat acggatgata tggtgtctta
     1801 gaacttgagc acttgagtgt ctatacaaaa ttacaaataa tcctgcttac ttgcagggtg
     1861 aattcagttt tgaggagcac acatggaaaa caataacttc atcagccaaa gaactgattt
     1921 ccagtcttct ttccgttgaa ccttacaaaa ggcccaccgc gagtgatgta tatctcctgc
     1981 tcccttttgt gattcttcgc acttgacttg atataactgt acttagctgt gtgcccattt
     2041 atcatgacaa cccagctttt gatgcatcct tgggtgatag gagactgtgc caagcaagat
     2101 cgaatggatg cagaggttgt cacaaaactg caaagattca atgccagaag gaaattgcgg
     2161 gcagcagcta tagcaagcgt cctgagcagc aaagtggcac tgaggacaaa aaggctgagg
     2221 agtcttttag gaacccatga tcttacctcg gaagagctag ataatctgcg gcttcatttc
     2281 gcacggatgt gagtatatgg gtttatctac aatatgttgt ctagtgcttg atatatcggt
     2341 aacagcgtta ctcagttaga cgagatgttc aaaatgtgga taatcccaat atggatagtt
     2401 gaccatgatg tataagcaga ttaaagtttg aatagttgca gtcccttgta agaagctgcg
     2461 tatttctgga atctgtaact ttgttgaatg aatacctgaa cttatttaca aagtggtcct
     2521 ggtgcagatg cgcagacgga gagaatgcca cgctaacaga gttcgagcag gtgctgaaag
     2581 caatgaagat ggactccctg atccctctag ctcctcgcgt gtttgatttg tttgacaaca
     2641 accgtgatgg aaccgtcgac atgagggaga tcctctgcgg gctctccaac ctgaggaact
     2701 cacgtgggga tgatgctctg cggctctgct tccaggtata tacccaaaat cacactccac
     2761 tagagaaaga tttgatgcta cagattgcag tagcaattca atgcatcatc tcgatgtttg
     2821 ctttcagact ggttcctaaa ctgacacgct aggttgattc aacatctgac aacaagaaac
     2881 atgatttcag atgtacgacg aggaccggtc aggctgcatc agcaaggaag agctggcgtc
     2941 gatgctccgg gtaagaggga ttgagactag ctctaccaga ccaagcgttc ttcagatggt
     3001 ttggtttaag atgttgacaa agctcttgtt ctgaaattgt gaaatttcta ggccctgccc
     3061 gaggagtgcc ttccgggcga catcgcggag ccagggaagc tggacgagat gttcgaccag
     3121 atggacgcca acggcgatgg caagatcacc ttcgacgagt tcaaggccgc gatgcagaag
     3181 gacagctcac tccaggacgt ggtcctctcg tcgctgcggc ccagcgggca ttagccagcc
     3241 tccttcagat taaatctgta gatgatgatc gaaacaaagc cccctgtctg tctgaatttc
     3301 attttcttgc cagtgccatg tggcgaactc tgtgatatga tcgtttcttc aaaacgtatg
     3361 ttgtggtgtg acatttcttt cgtacggatt tggtagctgg caccgcgtca actgacgcgt
     3421 ttttccatct ctggaagtca gctaactttt ctggatcagt tcttgatgta gtcttaccat
```

3481 ttaaaactta tttcggctag aatctgaaga tatcattttt tttttgcat aacgtaaatt
3541 tcaaaaagct ttggcacctt cttctgaaaa tggttgtgca gcacactcat aacctgcgtc
3601 atagcaattt gttacgtgca ttatttggtg attcttgtaa agccgaaaat gttcatacat
3661 tttgcaaaat tttacaaggt taagttttta cccatcctta gcttacatat ttttgcacaa
3721 ttttccgtgc ttggattttt ttttttgaa ttgcgggatg gttattgtgc cgaatagtat
3781 tttcctagta gtagatattt tgttgttccc cacccactga tagtcggcaa aaactaacgg
3841 ctcatcgctg ctccagcttc acatctgtca gctgcccatc tgccaaccaa atcattcgaa
3901 agcgcccgac tgaccgttcc tcctactcaa cacactcgcc gcagactgct agctactaag
3961 ctcagtagct tagctagagt cacaccacgc acggcacggc tcggcgtacc tacactacac
4021 ttcacgccgc cgcgatggcc cgcacgaagc acaccgtggc gaggatgccc aggctggagc
4081 ccagggaggc gccgccctgc ttcgagcgct cccgtccctg gaggccgccg ccgccgctac
4141 ggatggtgtc gccagagcct cggccggagc cggagaagaa gaagagggcg caccggtccc
4201 gcccgggcgc ggtggcgctg cgggagatca ggaaatacca gagcttcacc ggtctgctcc
4261 tccccttcgc gccatttgtg cgcctggtgc gtgcctccgt cgtgtcctct gctctgtcag
4321 tcagggtatc cttgtctgac cgtcatccat atatcgatga atttgcaggt taaggagatc
4381 accaactcct tctcgaccga tgtgaaccgc tggactcctg aagcgctcgt cgcgctgcaa
4441 gaggtcagtc agtgctcaaa ctctcacgta ctactgttac atttagagta ctcctggtac
4501 tttttaccga gcataaattt aaggcgaaac catcaatgtg ggatagatag atatatattc
4561 aggtaccgtc ttgcagctta atttcgattc aggaagctga atggcgtcgt ggtgatgatt
4621 tgtgacttgt gagtcataat gccgactgat tatctgtatc tagttccaaa aagaagtacg
4681 gaagaaccac tgaagttcaa tttttcattt agacaggatc gttaccgcac ctagtaactt
4741 actttagtat aggctcttgc tagaaatgcc ttcttctgca ccaccagcaa actaacagag
4801 gtcactatat tttgcgtgtc ctagctgtac ttccagacac tgtccttcat catattttgc
4861 tttgcattga atatggtttt ctcttctttt ccatatgcag gctgcagagt atcgcttggt
4921 agacttattt gaaaaggcaa atatctgcgc catccacgcc aagcgagtta ccatcagtaa
4981 gttgtcactg aatgagcact tcttttcgtt tgcacttatc ttatttttgg taattgcgaa
5041 ggtaacatgc caaagttatc aaagcatttc aatcacagta tcatctttgc ttttatttga
5101 ggtgatattt agttttatgt tatttgaagt tgcaaaaatt ataatggtta ataataagaa
5161 gatctacaaa tagcagatcc catctcaaca ggaccttcta ttcttttttc tcaacgagca
5221 tgtggaacaa acggtacata ttctgacagt tatggctagt gtgctagaat tttcaatcct
5281 gcatttagac accaaatctg gttggttacc attgcagaaa actctgtgtt tatttcctat
5341 attgagaata caaacatagt atcaaaataa gaatttttat ttgtaaaata tgtctgtatt
5401 ttctacttgc gtactgtttg tcgtaacaaa ttgatgtcac tcgtcatcat catcgttctt
5461 gtttctatct taacaaattg atggtatcaa ctagcaatta ctttatatat ataaagaaaa
5521 tacatgtata gattagcatg ccctcattca cctgaccttt tttgctacag tgcaaaagga
5581 catacatctt gccaggcgca tcgggggca aaggcattgg taatactaat ggaagatgct
5641 ttcttgggtc ggggtgtgca aatatagtgg tcataataat cggttacgtt tgtgagtgta
5701 tcattaacaa gtgtgggtgt gtattggtta gcttgtcggt tctgacttga tgcgtgttag
5761 ggctcagatg agttttcagc ctttgctcca ttttaattgt ctaattcaga gtttctgcga
5821 acaacactat gatatggatt ctgaaattac tagtaattgt tgctcactat tattactgtc
5881 attattatta ttatttttat tattattagg ggagcacagg gacaaggtag attcgaatta
5941 tttgcacaaa tgtgattcta gcattgcaca tgaatatgta cattcgtaac tgtatgagta
6001 gatgcacttt tggcacttcc tgtgcttcta ctattgttca ttctaggtag aggcacttca
6061 atgtttacac aaagatagtc tgctagtatt tcctttctct tgtttgcaat aatcttgtac
6121 ctaagagaac acttttacat gttcttttct tgatgaaagt gccattggct catctctagt
6181 tagccaaaat tcagacattt gactttgtaa ttatagctgc atctttctca ttacaactga
6241 atcagaactt tcctttaccc attaatgaac tattggtatg aacaactgta cctacttcag
6301 aattagggtg ctaagtgata gtagttctca tgacatgact tacattttga aagagcttag
6361 ttgttctctt ctaacaacta tgagagctct tagcacaata gagcttgttc gatcagtagt
6421 ttcaccctga atcttctaac aactatgaga gctcttagca caatagagct tgttcgatca
6481 gtagttaccc tttgtgagat tttaattaca ttacccatta caaggtaatg atcaatgggt
6541 gtaggtgaaa aaatccaata attgtatttg tggaatttga aaaatcatgt aattgattaa
6601 tgctaaaata tgatgaagga caattttctc ctggataaat ttaaatctaa aattttgact
6661 tataaaacta actccttatc tcttgtggcg agagtggaac ttattaatta tgtgtttgac
6721 tccattcctg tttattacat gtctaatatc attttctcta gaaagtttct agcaaaaatg
6781 acctctatca taaggaactt ccggtggaca cgagtggatt tagagccttc ttctaaacct
6841 ctttgtctgg cagcttggaa gaatattttc gaaccaaaaa agaaagggmt gcttmgggat
6901 tcgmcatctt caggctgtca attatgggct tatcctttct ccttcctgag aattgcatcg
6961 aatcctagaa gtcagctctc ctgtatcctt caatccaaat atttcagtga ctcgtcgatt
7021 tagaggccga agcccaaagt ccccaaatta gctctctaga cctcaaccct taatgttatc
7081 ccactgcttc aacaaaactc tttctgccag attgccatag ggaatttttc aatttggagt
7141 acgccttggt gcttgtcctg gaccaagatc gacgttgatc ttatcattca gcctcagggt

```
 7201 tatacctatc cagctaaggt gtctggtctt tggacccctc tccaaaagtc ttggaatgtt
 7261 ctccttattg attctctcct cctccctacc acaatggcct caatcaaatc cactcaaatt
 7321 ttaaactctt aagaggaaga tatcatttgt tggaattcac yccmamwgwk mwtkgyyamw
 7381 mwaaatctac tcaccgtgcg tstttacagt ttttgcaaga tgcgggtatg ccaagacaga
 7441 agaaatcaca aaaaatattc tcatgaggta tagaaatgta agaaaatgat tcatagggtc
 7501 aaaactttag tttgaaggat tttgaggaaa gtattgtgaa cgggagaaag agttgtcagg
 7561 tattcaaagc atattggaaa tatttgttgt aggtgctgat tggttgaaga tgatttacac
 7621 cttttcttca acattccttt tgctagagcg gcctggcttc tcagtccttg gtttataaga
 7681 tcagattctt tcgtagcaag cttgaactaa ctcatttcaa acctgctggc tattgatcat
 7741 ccttgtgctg acattcttaa catatgcacc tttctctggt gtctttggaa agcaaggaac
 7801 gaggaacttt tttgcaggaa aaaagacaac cctatcagat tgcaatcaca tcacatctct
 7861 tattgaggac ttaggaatgc ttggtggaac acacttggag gcttctcatg acaagaggaa
 7921 agaaactcaa cgctaattca aggttatatc gcccgcacac attttgtttt tacatgtccc
 7981 aaaatctact ctgatgcggc ttggatgaaa aggaacaact cttcttcgac gcctacaggg
 8041 caggggcaa tcttaatatg agggggcag aactagctaa aatcacacaa ataaggagct
 8101 gccatgactt gtgttcaaag gaaaatcatg agcataggtg gggctgcccc cccccccctt
 8161 gtcccccctt gtctccatcc ctgcctacgg gattgggaac ctacttccat gagcagatcc
 8221 tgagctctca cacggatttg gtcatcaaag ccaaatttgt gctagtttct tctactctgc
 8281 aagctgaagc ctaagcgata agcactgctt ccggtagatc actttgatgt tgcgatgaat
 8341 cttcagaaac ctattttctt cacagatagt tgtaactcgg acaaggcagt gtcggcttct
 8401 ggagtacaaa atccaacaat gctttgggag ataagaaggc aaacaattaa attccacaca
 8461 atttggttgt tcctttggac tcctcggtct ttcatgccag aagagaaacc aatggagtag
 8521 ctcactgttg ctcacaacag gctaaactat cgtttcgatc acaacctaca agttcatgta
 8581 ggaacacagc tcataccatt tcttcttgcc caattattgt tgctcttcag cagtctcagg
 8641 ttttggacat tgtaatcctt gatgtagaat gcttctgagc tgaatgtatg tttgggtcgg
 8701 tgctctgcac catccctatt aaaaaaaagt atccattagt catcgaaaaa tggtcatgcg
 8761 aaaaaccttt cctcctatgc actacatgcg agcaacaact actagatcaa agtaccacat
 8821 ttgcgggttc actaaaacca taacctcata gtccaaaata tcgatgccgc gcaagaaatc
 8881 cgctaatcga accctaccgt accggttacc ctaatagatc tagtatgcat gtcattggaa
 8941 tctctagcac tatatacgtg tccgttcaga tctacgaaaa aggagcatcg aagaagatag
 9001 tagttgtgat tgctgccatt ggagacggtc gatcgtatcc tagatcgagg ttgtgtgcag
 9061 gccgatgaaa cctcgctcca aaccggccgc ggtggatgtc accttgcgct tcgagtcccg
 9121 tccccacttt ccctcgtcat cttgcgaagt agtggattcg tcggcggtag cgcagccgct
 9181 gtagcgatgt gacaggatgg tggttagagt gagacggggg agacggggag agtgggcggt
 9241 gtggcgattt atgatgtgtg ttcgtgaaga aacgcggtgt tgtcctctcc ttccctgcaa
 9301 gtgcaactga gagatgagaa tagagcaggc tccaaagaaa cgatcgattc aacagtatct
 9361 accaggcgaa gcctagttat gatctaagaa ccgcgctgat acaacgcaca cggcctacca
 9421 gaacaaccga ttgggccaaa ttatattcca ttgagcctgg ctggccgaaa tcgtgcatca
 9481 ctgggcgcct gtgcgcgttg cgaacgagcc gtcgaccact tttggcccat catttggtag
 9541 cctgtgctgc accggcccga atagagttgc aaattgtttg gatgcctgga gacagcgttc
 9601 catctctgtt cagccacaac acatgtgttt ggttgccatt ttgggcaccc tgataagagc
 9661 ttctcctcac catattcaaa tatggtgacc ttaccatcat ataacggcac acaaacagct
 9721 caaacacgat cataagcaca acaaacactt atacacaacg aacatagtac ttcacatgtc
 9781 ctaatctagt gtcagagtgg taagtcgctt gcctaaacct gggggcagac tccccaggtc
 9841 caaaagtagt gttcaaacgc ctcctagagg ccagcacagg taacatgatc agacataaac
 9901 ataggcagcg atggagcagc aacaccttaa tggtgaggat tagcaaaagg gccctcgcgg
 9961 tgcctcttgc agccgaagac gctggagcag gacgtctcct ttcctgaaga cggcgaactt
10021 gaagtcgtcg tcctaagggg tgaagatgas cgtgtcgtcg acgtgcagca aaatcctggt
10081 gcagaactcg ctccaggact tgatgamccc gacgcgctgc cctgtccact gcagctgcac
10141 cttccactcg caacggtcgc ccaagtataa gcagaccttc acatgggggt cattgttctt
10201 gagcttgtac ttcctgatga ggggctgctc catgtacnnn nnnnnnnnnn nnnnnnnnnn
10261 nnnnnnnnnn nnnnnnnnnn nnnnnnnnnn nnnnnnnnnn nnnnnnnnnn nnnnnnnnnn
10321 nnnnnnnnnn nnnnnnnnnn nnnnnnnnnn nnnnnnnnnn nnnnnnnnnn nnnnnnnnnn
10381 nnnnnnnatg caaggatgat atgaatgcat gatgttttta aaattttgaa aacttgggat
10441 gttacagaac cactctccac cctgcttcaa atccagctag gtaagtctat taaaaattaa
10501 actattccta gaattccaaa tccccgacaa gacagcagaa gagatgaaat taaattgcaa
10561 gaaatttttt gttacaaagt tatctactag ctatgtctaa gtacttttcc accttatgat
10621 taaacatttt ctcaatcaaa agccaaatgc ttctggacac tatgcaatca aagaaaagat
10681 gatgcacact ctcaaattct ttgcagaaag agcactctat tggtttggga atacccctac
10741 gtctcaaatt atccctagtc atgattttat tctgtgaaaa caaccataaa aaacctggac
10801 tctatgagga attttcaagt cccaaactgc atgcaagaaa atatgaatta ctcccataaa
10861 attgactaaa gcatacatgg atttcgaaga atataaatca tttgtttcat acgaccacat
```

10921 aagttgatcc tcgtcagaat tcaaatctat ggtattagca ataaccgaaa ctcctgccat
10981 tgcactatca tatcatcaga gaaagttctc ctaaaatcac atctcagagt tgttccatcc
11041 tacacctcac taacagattt aatttgttga ttagagacaa attatatatc caaaactgcg
11101 tagctaaatt agtattacca aaccaaatgt cttcccaaaa tctaacactc ctaccgtttc
11161 caattttcca tttataacca agcctcactg cttgtgcagc ttccatcact cccttccaaa
11221 aagttgatgg atgagcatca tggcagcaaa gtatattggg attcctagta ttatatttat
11281 tatctattac ttttcccaca gagtcccttc atcatgtata tatctcttaa cccaagatcc
11341 tagtacataa atatttaagt cttgtaggtt cgggatacct aaacctctaa actctttcct
11401 catacaaaca aacagaatgc cagttagcta aataaatctt attattccct tcttcagatg
11461 tgcattccac acaacagtta gctaactgaa tgtttattag tttcaaagtc cacttggggg
11521 cgacaggacg cgtctctcgt ttccacggaa atgacaggtc ggtaacggaa atcaggttaa
11581 cacatggcat attttggtgg ttttgccaaa taaaatgacg gtgcaattcc gtggcaacca
11641 aacaacataa ctttggaatt gccaaatgga taccaaacga gctcttagca gttgactgag
11701 aattaacgga accataataa aatatgaaag tattcggcaa gttccgttgt gaacctatcg
11761 cgaaccgctt tcacammcac ccctgccact gccacctggg cccgcgcgcc caacgttcga
11821 ctcgcgctcc taaccggcct ctactcccac acatagcgcc ttttttttcta ggggaagacc
11881 acaactgact tcacttgcta cctgcgacaa tcccagggtc tcgtttgaaa aaaaaaaaag
11941 acaagagcca tcctcgccgc cgtcgagagc ccggccgccg cgatggctcg cacgaagcac
12001 ccagctgcga ggaactccag gccgcagccc aaaaagcagc tccagttcgg gcgctccccc
12061 ggcctggggc cgcagcagga gacaggtgag cctcccatcc ctctcctgcc ttacgagcga
12121 atacgccgcc tctttgatgc ggaaatgcta agctcgtgct ctcttttgca ggcggcacga
12181 gtacgtcgga ggcaccggtg cgtgtgagcc tgtctatctg ggtttctctg tttcgttttg
12241 tctttttctt ctgatttgct cgttacatgt tcggcggaat gcagaggcga ggtgggcgaa
12301 gggcggctgc agcgacgact caaggtgcgg tcttcttgtg tcattctctt gttttatgtc
12361 acgtgtttag ggctttaggc tgctttctcg ggtcatttcc gatcattccc cttccttact
12421 gtgaatattt ggtggggtta caactcaagg tgtaatttgt atgcccctct cgttgctgtt
12481 ctttgctcct accgctggat caacagtttt actttactac cctggtgcag tttttttgtt
12541 tatcttctat tgacatgatg acgagggtgg acatggaact ctcagttttg tttggagttg
12601 ctctctgcca ggtagctgtt atttgggttt gtgactgtct ctagtttgta ttttttcttg
12661 aagaagcagt ggcacctgtg caacagaggg tgaagaagcc gcaccgattc aagccaggca
12721 ctgtcgcact gcagcagatc aggaagtacc agaagtccac cgagcttctc atcccgtttg
12781 caccctttgt ccgtctggtg ggtacctctg ttaagtcccc tctctatcga cggtttagat
12841 cgcagagtgt gatgggagat attgttgttg ataaacttgc aggttaagga ggtcactaac
12901 ttctgctcca ccaaggtgta ccgctggaca cctcaagctc tcgctgcgtt gcaagaggtc
12961 aatgctgaaa cctgtcatgt accattggac ctgatggttt gattagagaa ccatactgct
13021 tgttaaccta agatcgaaaa ccatgaattg tgtgatagat aaatttatcg ccataattca
13081 tagtttgtta atgctctggt attaattctg ttgatgtatt tatttatcaa cacttattca
13141 aaagaaagag aaagtattgt ctgggtttaa cctttgtttg tagtagcggc ttattttttt
13201 atggctggta actgaaactg aaatgtaatg atgagtggaa gggtatccgt agtctttagt
13261 gtgtctggtt gtttgttcta agatatacat cattacgtca cgcatgcaaa actgaactat
13321 tatttaggtt ctattttttta aaaagaataa gtttgtaact atgcggtgaa ggaagaccta
13381 gtgatcagtg gcaccttaaa tttgaatcag gaaggatata gtgtgatgga gaagattgaa
13441 tgttgtcatg tgatggttat gaggcctaac agtgtaaact atgcagtgaa ggaagaccta
13501 gtgatcagtg tggtacctta aatttgaatc aggaaggata tagtgcgatc ctgaagattg
13561 aatgttgtca tgtgatggtt atgtgtccga acagtgtatc ttatatgtat ccagtacaac
13621 aacataacta ctttggaacc ataaagttta ttgtccattt atagaacatt gctaatgtat
13681 ggaaaactta ctttagtcgc ctcttgccgg aatgtattat tctgcatgac cagcaaaata
13741 gcaaagagtg gactagtcca tttgacctat gaataagtta atgtcctaac tgtactgtca
13801 attactatcc tgcttgctcc tcacatgatt ttctttccct ttccctctgc aggctgcaga
13861 atatatgttg gtagacttat ttgaaagggc aaatctctgc tccatccatg caaagcgtgt
13921 taccctcagt aagtcggcac tgaatgaaca cttcttttca tttacagtat tatgcaacag
13981 gaaacatgct agaattgtta aagcactcga attacagtgt catctttgct tttatttgat
14041 atgatattta gttgcaaaat ttgcagtgat taatgacact aagacctaca attatcagat
14101 atcttttgtt ctccacgaac atgtcaaaca aacagttaat atgctaacaa tatggtttct
14161 gagttgccta gaattttctc atgctgcata tttagataag taccaaatct ggtgagtaca
14221 acaggaaatt tagggtaact aattacatgt gtatttgtta tcttgaggac aaacaggcat
14281 caaatcaata tttttactgt tgtaatatat gttttatttc tttcttaaag ctgccaactg
14341 ttgtatggta acaaacacac aaaaaacatt atgtatcatt gttattcttg tatcgtaaca
14401 aayaatacat gttttcaacc agctttgatt gtatatatct taagaaggat gctccttaat
14461 aaaagcgact attttatgaa gaaattacat gtatagagta gcatgttcac atttacctga
14521 cttgttttta ctgctacagt gcagaaggac atccatcttg ctaggcgtat cggggggcca
14581 aggtggtgat actaatggaa catattttct tcatcccagg ggcaaagtcc gttgcgatac

```
14641 cgatgggaga tattttctgt attgtggggt gcaatggcag tggtgatagt gatcaaatag
14701 acaaatatca gtggtgatac tcattgttag gaagtattgt acaccctccg acccatatta
14761 cttgctagta atatagatgt atctagaaat attttagtta tatactagtg gcaagtaata
14821 tgaattgggg ggagtagttg tatcttcatt tagttagctg gtcttcgaag ttgattctta
14881 tttgttatgt atatcttagg ggtcatttga ccttcccagc ctttgcgctt tctgttaata
14941 gtccaagaat gcgattgtgg aataacttgc taagaggttc ctttgatctg ctttgtaaga
15001 ggttctcctc tccaccttgt atcggttcgg tcgttgcggc tttatttata aagcgggaca
15061 aaagccaatt tcaaagagaa ataacttgct attgggagtg atgttttggc acatgggagc
15121 gtatgcaccc tttattttga aatacatgtt agacatattt taaaatgtca aaaaaatctt
15181 aaaatgtcaa aaaaatcgaa acaaaaattt cgcacgtaca tcttcatatg ctacatgctc
15241 acaaagtcgt ttcatgaaaa attgatatgt catgtggcgt gtgtaaaaag acaaaattcg
15301 gtgctgaaac aaagacttat cacaagataa attttctctt tttcgctaga ctacaaaaaa
15361 tatcattttt ttgtgaaact tgacgaatac acatatatta tggagatgta aatgtaattt
15421 ttttgtcaaa aaattttaac acatgaaaat atgtttttat ggtagaggga tcatacgcac
15481 ccgggagccg aattgagttt ctgcttgtta ttgttgtgga caaaagccaa tttcaaggaa
15541 atgcgtgtac aaacatttga tactatttgc tctgatctta ttactccctt cggattcata
15601 ttatgtgtcg tttgtatgga tttatctaga catatattaa ttctggatac atccataata
15661 gcatagctat aagcaatatg aagcggagag agtatttctc ttttaaaatc ctgtctattt
15721 accggccatg gagagcgagc agaacatgtt gtatcgtgga ggtacgggcg catagttgtt
15781 atgtggggcc acacacttgt atcttttgag taagatttat ccctagtcat gatctgatgc
15841 agtgcaaaag ttcagtaaca accacttgta gcggcatgtg cagaagagct tcagttaacc
15901 actgccgatg ttgatcgaac aagacgtaca tccaacggtt atcatggaag gcatcttcgc
15961 ggcatctcct ctaggctcac atgctagtgc tcgtttcgat ttgctgcttt acaggtcacc
16021 atgtttgtaa ggctataagg gcatgtccaa ttcactgccc tagaggtact gcttcacacc
16081 tttagcnnnn nnnnnnnnnn nnnnnnnnnn nnnnnnnnnn nnnnnnnnnn nnnnnnnnnn
16141 nnnnnnnnnn nnnnnnnnnn nnnnnnnnnn nnnnnnnnnn nnnnnnnnnn nnnnnnnnnn
16201 nnnnnnnnnn nnnnnnnnnn nnnnnnnnnn nnnnnnnaca gtattatgca acaggaaaca
16261 tgctagagtt gtaaaagcat ttcaattaca gggtcatctt tgcttttatt ttatgtgata
16321 tctagttttg atgttatttg aagctgcaaa aattgtagtg attaatgata ccaagaccta
16381 caattatcag actttagcag gtcctcttat cttttgttct caatgaatgt gttaaaactg
16441 atcctaattt ttctcaagct gcatgtttag ataccaaatc tggttactat agcaagaaat
16501 tcagggttaa ttgcctgttt atttcttatc ttgaggatac aaacaagcac cagatcatat
16561 tttttacttg taaaatatgt ttttattaac aaacacacta gaatcatttt atatatctta
16621 agaaggaagc tcctgaataa aagtgactat tttatgaaga aaatacatgt ttagcatgtt
16681 cacattcatc tgactttctt tttgcttcta cagtgcaaaa ggacatctat ctcgctagac
16741 gcatcggggg acgaaagtgg tgataccgat ggaataacat cattgttagg aagtatattg
16801 tagctgtttc ttcatttagt tagcatgtcg tagtcatttg acctttcagc ctctgcgctg
16861 ggattgtcgt agtcattttg gagcttccac aaacacttga tttcgttgtg gatttcatgc
16921 tactagtgga gcaacaggag gaggagcaaa tcagaaatgt gtgtacagac atttgaaacc
16981 atatctctct aatcttatta tttctctcgg ccatggagag cgagcaaagc atgctgtgca
17041 gctgctattt attttattat ttcaaccagt gtctaaaaag tgcctgtccg acttagttat
17101 tttttattat ttcaaccagt gtctaaaaag ttctgaactt ctctctctaa tcttattatt
17161 tctctcggcc atggagagcg agcaaagcat gctgtgcagc tgctatttat tttatttttt
17221 caaccagtgt gtaaaaagtg cctgttggac ttagttattt ttaattattt caaccagtgt
17281 ctaaaaagtt ctgaacttct ccaagattcc tcgggaggga agaaagtgaa ttctaaaaat
17341 agtctcgatg aacagcatgg gatcagcatg aatttttgtg ataagaaagg aatttaggct
17401 ggtccacaca tcctctcagt tctaatccca acccaggatc tgatgcagtt caaagttcac
17461 aatgacaggc acttgtatcg cccccaggca caccattctt ccatagatgc aacaacgatg
17521 gaattggctg tcaatttctc agccaaaata tgtttgatga aacagagctg ttcatcaagt
17581 tataccactc tggcagaaac agaaagcaaa agacactttc tatggccacg gcaacataca
17641 tgatcatgat gaacaagaga cagctatttt aacttatcat atacaaggaa gaagctccga
17701 tataaaatta aacatgtgcc tggcagattt acaaactgac aaagcctcgg aagagatcat
17761 ctaggacatg ctaggggccg ctgctactga cctacaaact gtgaaccatc gggctgcgac
17821 aagctagaac ctcgaactac cataccagct gtttctaccg cgagcacggg gaatatatat
17881 gcggtgtaat ctacaaaaat taatatgaac atcgtcatcg ccgtcgctgt caaacagggg
17941 aggaacttgt tcttcggagc cgatacttcg gttctggggg tggcgcggag atcattagca
18001 tgtccaactc ctgaaaattt tgtgaaattg aaaaataaaa acagtaagta tcattagaca
18061 catcagacat acatgaacac cactacaccg taagctagtg gattgcaaag aagtgtaagg
18121 ctaagaatta tgcatagcca caaggtacgg agcatcataa tttcatctag gactgtaaga
18181 tcatggcatc acagaacaaa tttacatgga gggatatgtt tatgaacatt tgctacatat
18241 aattaacata atcagcatca tctccaagta ataatatgat atgttcaata gataaatgag
18301 cgtcaccatt tgaaggatca aatgaacaar ataaagaaac acaagcatag gtgtcctgtg
```

```
18361 acttgacatg taattacgat ttaatgagta ctaataacta atgtgttgat ttctaacagt
18421 tatatgtttt gggttgtaag attttattcg cactaatgca ttgatctcta gtaattttaa
18481 tccatatgtt gagatgtatg caacatatag aacaccctgt caaacattaa aatgctggtg
18541 aaatgatgat aattaacgtt ttaaaacatt gaaacctagc ggccaagtta atgcagtaag
18601 acaagtcagt aaatcagggc tcaataacat tctgaagaac tccataagag ttccctctga
18661 atgtcgaata tacagctgat catatttcca acaattataa tgttcacact aacacaatct
18721 agccaaattt tgtgggctca aacttgcata tacttacata cattatagca tgacctggta
18781 tgtttcacct ttcaaaagaa gacttagcat aatcgttatt tcaatcccaa aacttagtta
18841 gattagctca gtaatcaaga aaagtaactt tgagactctt ctttcttgac acaaaaatta
18901 gtttgactat ctgaacaaga acagtgttaa aagaacctac agttcactgc aaggcatagt
18961 ggaataacct agttttaaat atcctccccc aaaaagaagg gggaagttaa cagtattatg
19021 ataataggaa ataaggtacc ttctgcctct ttagcctctc gttctcctct tcgagatgag
19081 ataccttgtt ttcaagctca ttagtgtaag cctgccaagc agaatgcatt agaatggcgt
19141 agaagaacaa cacgccacta cagtgccata gaacctaata atatggttgc agtcttaggc
19201 taaactatgc aagagatata ccgaaatcaa tatattcgag gataattttt cattacgctt
19261 tgatgttgca atcagaaata tagcaaaaca aaactcaata cataggagat cagaagtagg
19321 caggacaata gaagtagacc tgcttcctcg ctctcgatcg cgcagccgat tccctgtttt
19381 ttatcattct cttctgcctt ctttctacaa ccttatcagg tacaccgcct gaggaggcac
19441 cacgtttgcg cccaggtgtt tggggatctg aaagtgcact gagcatgggt gaagtgatct
19501 gggcatccaa gtaaatcgat tccaagatag ctcctgtagc gacagccagt ggctgagggg
19561 ccaactgagt tgccatgaaa gaacctggca tgcttggatg tggaggctcc aaagcttgct
19621 gctggtactg ctgtaaccac tgtgcctgtg ctgtcgagct caaactgggc gcaccagccg
19681 cgattacact gctcccagct gcatccatgt taggtgggaa gtcactcgac gaatccttca
19741 aacacccttc cgtgacgacc ccagctttca gcaggaaatc ctcgagtgtc atctccccaa
19801 gcgtaagctg cctctcccgc ctccgccaac caccctcctc gccgcttttt ttcttgggcg
19861 aatccaggat gcccttccac acctcatcaa cagtcttctt gctgagctcc ggaggcatgg
19921 tgatgctccc ctggcgatgc aggcccgagg gcggctcaga ctggccggcg acggcaccat
19981 cggggtccac gccatcagga aacacgctct tgagcagctc atcaaggttc atactgagca
20041 agggctctgt cagctggttc tgcacctcgt cgagggtaag gctgtacaag gatccttgcc
20101 tggccaggcc ctgcggctgc ctgcccgacg ggccagcgta gccaccgcct ccagcctgcg
20161 actgcgaatg cgacgccatt gcctgaatca tgaacaggcg ctcgcttgaa ctggtcgccc
20221 ctatggggat tccaccccca caaagacctc gggagcgacg acgcccaaag cagcctcctt
20281 tccggcggca gagccttggc acagagatca ccgaaacgca gatccacgag accgtaacac
20341 agcacgcacc agcgaattga acctgctaca gatagacatc agggttgcat caggttgaat
20401 cgcatagttg ttccgcacaa gacactactt tggatgagaa ttacaagggc gaacaagaag
20461 aacaggcagg gaatgtaatc caacctcgaa ttatctctgg acacgacggt aaccagcgga
20521 ttcgagggta gtccggcggc cggatctccg ccgctccaac cttccagctg gaacgtcgcg
20581 cccgggagtt ccgcgcgatt cccgcccgcc ttgcgctgga tcgaggcccc gcgcggtcag
20641 tcgtggttcg ggctgctggc agctaccatt ggggagggag ggaggaggag ggggcgacgg
20701 tggggttccc gggagaggca gaggaggcag ccatggtgga agtgggactg gaggagaaga
20761 aagaaactgc tgctgggggt tctgatgggc aacgggggaa atgggcccac tctgaagcgg
20821 agatgg
//
```
