# Supplementary material for: The origin and evolution of a two-component system of paralogous genes encoding the centromeric histone CENH3 in cereals
Source: BMC Plant Biol. 2021 Nov 18;21:541. doi: 10.1186/s12870-021-03264-3 (PMC8603533; doi:10.1186/s12870-021-03264-3)
Supplement: Supplementary file 5 — Additional file 5 The sequences of mRNA αCENH3 assembled for this study. [file 12870_2021_3264_MOESM5_ESM.pdf]

Additional file 5. The sequences of mRNA *αCENH3* assembled for this study.

```
>H.spontaneum
ATGGCCCGCACCAAGCACCCCGCCGTGAGGAAGTCCAAGGCGCCGCCCAAGAAGAAGATCGGGTCC
GCTAGCTCCCCGAGCGCGGCGCAGCGCCGGCAGGAGACAGATGGCGCCGGCACGTCCGAGACTCCG
AGGCGGGCCGGGCGGGGGCCGGCCCCAGCGGCGGCTGAAGGGGCACCTGGGGAACCGACGAAGAGG
AAGCCACACCGGTTCAGGCCAGGCACGGTGGCACTGCGGGAGATAAGGAAGTACCAGAAGTCGGTC
AATTTTCTCATCCCGTTTGCACCGTTTGTTCGTCTGGTCAGGGAGATCACCGAATACTACTGTCCT
CGAGTCAAACGCTGGACTCCCCAGGCGCTCCTCGCAGTTCAAGAGGCTACAGAGTATCACCTCGTC
GACATATTTGAAAGGGCACATCTCTGTGCCATCCATGCAAAGCGTGTTACCGTCATGCAAAAGGAC
ATACAGCTCGCAAGGCGTATCGGCGGGTCGAAGCTTTGG
>H.brevisubulatum
ATGGCTCGCACCAAGCACCCGGCCGTGAGGAAGTCCAAGGTGCCGCCCAAGAAGAAGATCGGGTCC
GCGCGCTCCCCGGGCAGCGCGCAGCGGCGGCAGGAGACAGATGGCGCGGGCACGTCGGCGACTCCG
AGGCGAGCCGGGCGCGGGGCGGCGGCTGAAGGGGCACCTGGGCAACCGAAGCAGAGGAAGCCACAC
AGGTTCAGGCCAGGCACGGTGGCACTGCGGGAGATAAGGAAGTACCAGAAGTCGGTCGAGTTTCTC
ATCCCGTTTGCGCCATTTGTCCGTCTGGTCAAGGAATTCAGCGAGCTCTACTGTCCTGGAATCACA
CGTTGGACTCCCCAAGCGCTCGTCGCCGTTCAAGAGGCTGCAGAGTATCACCTCGTCGACGTATTT
GAAAGGGCAAATCACTGTGCCATCCATGCAAAGCGTGTTACCGTCATGCAAAAGGACATACAGCTT
GCGAGGCGTATCGGCGGGAGGAGGCTTTGG
>H.vulgare
ATGGCCCGCACCAAGCACCCCGCCGTGAGGAAGTCCAAGGCGCCGCCCAAGAAGAAGATCGGGTCC
GCTAGCTCCCCGAGCGCGGCGCAGCGCCGGCAGGAGACAGATGGCGCCGGCACGTCCGAGACTCCG
AGGCGGGCCGGGCAGGGGCCGGCCCCAGCGGCGGCTGAAGGGGCACCTGGGGAACCGACGAAGAGG
AAGCCACACCGGTTCAGGCCAGGCACGGTGGCACTGCGGGAGATAAGGAAGTACCAGAAGTCGGTC
AATTTTCTCATCCCGTTTGCACCGTTTGTTCGTCTGGTCAGGGAGATCACCGAATACTACTGCCCT
CGAGTCAAACGCTGGACTCCCCAGGCGCTCCTCGCAGTTCAAGAGGCTACAGAGTATCACCTCGTC
GACATATTTGAAAGGGCACATCTCTGTGCCATCCATGCAAAGCGTGTTACCGTCATGCAAAAGGAC
ATACAGCTCGCAAGGCGTATCGGCGGGTCGAAGCTTTGG
>A.sativa
ATGGCTCGCACGAAGCACCCGGCCGTGCGGAACTCGAGGCCGCCGCCCAAGAAGAAGCTCAGGTTC
GCGGCCCGGACGGCGGAGCAGGAGACAGGCGGCGCGAGCACGTCGGCGGCACCGAGGCGAGGCGCG
AGGAGGCCAACCGCAACGCCGGCTCCAGGGGCACCTGAGCAACAGAGGACGAGGAAGCCGCACCGG
TTCAAGCCAGGCACGGTCGCACTGCGGGAGATCAGGAAGTACCAGAAGTCCACCGAGCTGCTCATC
CCGTTCGCACCTTTCGTCCGTCTGGTTAAGGAGGTCACTGACTGGGCGTCACCCAAAGTGACCCGC
TGGACTCCTCAAGCGCTCGTTGGGTTGCAAGAGGCTGCAGAGTATATGTTGGTAGACTTATTTGAA
AGGGCAAATCTCTGCGCCATCCATGCAAAGCGTGTGACCCTCATGCAAAAGGACATCCATCTCGCC
AGGCGTATCGGGGGGCCAAGGTGG
>A.speltoides
ATGGCCCGCACCAAGCACCCGGCCGTCAGGAAGACCAAGGCGCCGCCCAAGAAGCAGCTCGGGCCC
CGTCCCGCGCAGCGGCGGCAGGAGACAGATGGCGCGGGCACGTCGGCGACTCCGGTGCGCGCCGGG
CGGGCGGCGGCCCCAGGGGCGGCTGAAGGGGCAACTGGGCAACCCAAGCAGAGGAAGCCACACCGG
TTCAGGCCGGGCACGGTGGCACTGCGGGAGATCAGGAAGTACCAGAAGTCGGTCGACTTTCTCATC
CCGTTTGCACCATTTGTTCGTCTGATTAAGGAGGTCACTGACTTCTTCTGTCCTGAAATCAGCCGC
TGGACTCCCCAAGCGCTCGTTGCAATTCAAGAGGCTGCAGAGTATCACCTCGTCGACGTATTTGAA
AGGGCAAATCACTGTGCCATCCATGCAAAGCGTGTTACCGTCATGCAAAAGGACATACAGCTCGCG
AGGCGTATCGGCGGGAGGAGGCTTTGG
>A.tauschii
ATGGCCCGTACCAAGCACCCGGCCGTCAGGAAGACCAAGGCGCCGCCCAAGAAGCAGCTCGGGCCC
CGTCCCGCGCAGCGGCGGCAGGAGACAGATGGCGCGGGCACGTCGGCGACACCGAGGCGAGCCGGG
CGGGCGGCGGCCCCAGGGGGCGCTGAAGGGGCAACTGGGCAACCCAAGCAGAGGAAGCCACACCGG
TTCAGGCCAGGCACGGTGGCACTGCGGGAGATCAGGAGGTACCAGAAGTCGGTCGACTTTCTCATC
CCGTTTGCACCATTTGTCCGTCTGATCAAGGAGGTCACCGACTTCTTCTGTCCTGAAATCAGCCGC
TGGACTCCCCAAGCGCTCGTCGCGATTCAAGAGGCTGCAGAGTATCACCTCGTCGACGTATTTGAA0
```

AGGGCAAATCACTGTGCCATCCATGCAAAGCGTGTTACCGTCATGCAAAAGGACATACAGCTCGCG
AGGCGTATCGGTGGGAGGAGGCTTTGG
>aT.aestivum
ATGGCCCGCACCAAGCACCCGGCCGTCAGGAAGACCAAGGCGCCGCCCAAGAAGCAGCTCGGGCCC
CGCCCCGCGCAGCGGCGGCAGGAGACAGATGGCGCGGGCACGTCGGCGACACCGAGGCGAGCCGGG
CGGGCGGCGGCCCCAGGGGGGGCTCAAGGGGCAACTGGGCAACCCAAGCAGAGGAAACCACACCGG
TTCAGGCCAGGCACGGTGGCACTGCGGGAGATCAGGAGGTATCAGAAGTCGGTCGACTTTCTCATC
CCGTTTGCACCATTTGTCCGTCTGATCAAGGAGGTCACCGACTTCTTCTGTCCTGAAATCAGCCGC
TGGACTCCCCAAGCGCTCGTCGCGATTCAAGAGGCTGCAGAGTATCACCTCGTCGACGTATTTGAA
AGGGCAAATCACTGTGCCATCCATGCAAAGCGTGTTACCGTCATGCAAAAGGACATACAGCTTGCA
AGGCGTATCGGCGGGAGGAGGCTTTGG
>B.distachyon
ATGGCCCGCACGAAGCGCCCGGCCATCAGGAAGTCGAAGCCGCAGCCCAAGAAGCAACTCCAGTTC
GAGCGCACAGGCGGCGCGAGCACCTCGGCGTCGGCGACCCCGGGGAGACGTGGAGGCCGGACCCCG
GCGCGAGCGGCGGGTCAAGCGGCACCCGCACAACAGAAGCCGAAGAAGCCACACAGATTCCGGGCA
GGCACGGTGGCGCTGCGGGAGATCAGGAAGTACCAGAAATCCCCTGAGCTGCTCATCCCATTCGCA
CCCTTCGTCCGTCTGATTAAGGAGATCAGTAATTTCTACTCACCTGAGATCTCGCGCTGGACTCCT
CAAGCTCTCGTTGCTTTGCAAGAGGCTGCAGAATACCACTTGGTAAACATATTTGAAAAGGCAAAT
TACTGTGCCATCCATGCGAAGCGTGTTACCATGATGCAAAAGGACATACAGCTTGCGAGGCGTATC
AGTGGGCACAGGGGGTAC
>B.stacei
ATGGCCCGCACGAAGCGCCCGGCCATCAGGAAGTCGAAGCCGGTGCCCAAGAAGCAACTCCAGTTC
GAGCGTGCAGGTGGCGCGAGCACCTCGGCGTCGGCGACCCCGGGGAGACGTGGAGGCCGAACCCCG
GCGCGAGCGGCGGGTCAAGGGACACCCGCACAACAGAAGCCGAAGAAGCCACACAGATTCCGGCCA
GGCACGGTGGCACTGCGGGAGATCAGGAAGTACCAGAAATCCTCTGAGCTGCTCATCCCATTAGCA
CCCTTTGTCCGTCTGATTAAGGAGATCAGTAATTTCTACTCACCTGAGATCTCGCGCTGGACTCCT
CAAGCTCTCCTTGCTTTGCAAGAGGCTGCAGAATACCACTTAGTAAACATATTTGAAAAGGCAAAT
TACTGCGCCATCCATGCGAAGCGTGTTACCATGATGCAAAAGGACATACAGCTCGCGAGGCGTATC
AGTGGGCACAGGGGGTAC
>B.sylvaticum
ATGGCCCGCACGAAGCGCCCGGCCATCAGGAAGTCGAAGCCGCAGCCCAAGAAGCAACTCCAGTTC
GAGCGCGCAGGCGGCGCGAGCACCTCGGCGTCGGCGACCCCGGGGAGACGTGGAGGCCGGACCCCG
GCGCGAGCGGCGGGTCAAGGGGCACCCGCACAACAGAAGCCAAAGAAGCCACACAGATTCCGGGCA
GGCACGGTCGCGCTGCGGGAGATCAGGAAGTACCAGAAATCCTCTGAGCTGCTCATCCCATTCGCA
CCCATGGTCCGTCTGATTAAGGAGATCAGTAATTTCTACTCACCAGAGATCTCGCGCTGGACTCCT
CAAGCTCTCGTTGCTTTGCAAGAGGCTGCAGAATACCACTTAGTAAACATATTTGAAAAGGCAAAT
TACTGCGCCATCCATGCGAAGCGTGTTACCATGATGCAAAAGGACATACAGCTCGCGAGGCGTATC
AGTGGGCACAGGGGGTAC
>bT.aestivum
ATGGCCCGCACCAAGCACCCGGCCGTCAGGAAGACCAAGGCGCTGCCCAAGAAGCAGCTCGGGACG
CGCCCCTCGGCCGGGACGCCGCGGCGGCAGGAGACAGATGGCGCGGGCACGTCGGCGACTCCGAGG
CGAGCCGGGCGGGCGGCGGCCCCAGGGGCGGCTGAAGGGGCAACTGGGCAACCCAAGCAGAGGAAG
CCACACCGGTTCAGGCCAGGCACGGTGGCACTGCGGGAGATCAGGAAGTACCAGAAATCGGTCGAC
TTTCTCATCCCGTTTGCACCATTTGTTCGTCTGATCAAGGAGGTCACCGACTTCTTCTGTCCTGAA
ATCAGCCGCTGGACTCCCCAAGCGCTCGTTGCAATTCAAGAGGCTGCAGAGTATCACCTCGTCGAC
GTATTTGAAAGGGCAAATCACTGTGCCATCCATGCAAAGCGTGTTACCGTCATGCAAAAGGACATA
CAGCTCGCAAGGCGTATCGGCGGGAGGAGGCTTTGG
>D.glomerata
ATGGCCCGCACCAAGCATACGGCGGAGAGGAGCACCAGGCCGCTGCCCAAGAAGCAGCTCCAGTTC
GCGCGCGAGACAGGCGGCCCGAGCACGTCCGCGGCACCGAGGCGAGGCGCGCGGAGGCCGGCAGCG
ACGGCGGCTCAAGGGGCACCTGCGCAACAGAAGCCAAGGAAGACGCACCGATTCAAGCCAGGCACG
GTGGCACTGCGTGAGATCAGGAAGTACCAGAAATCCACCGAGCTGCTCATCCCGTTCGCACCCTTT

```
GTCCGTCTGGTTAGGGAGCTCACTCGAAACGCGTCAATAGAGGTGGACCGCTGGACTCCTCAAGCG
CTCATTGCGATCCAACAGGCTGCAGAGTATCATTTGGTAGACTTGTTTGGAAAGGCAAATCTCTGC
GCCATCCATGCCAAGCGTGTTACTATCATGCAAAAGGACATCCAGCTCGCTAGGCGTATCGGGGGG
CTAAGG
>dT.aestivum
ATGGCCCGTACCAAGCACCCGGCCGTCAGGAAGACCAAGGCGCCGCCCAAGAAGCAGCTCGGGCCC
CGTCCCGCGCAGCGGCGGCAGGAGACAGATGGCGCGGGCACGTCGGCGACACCGAGGCGAGCCGGG
CGGGCGGCGGCCCCAGGGGGCGCTGAAGGGGCAACTGGGCAACCCAAGCAGAGGAAGCCACACCGG
TTCAGGCCAGGCACGGTGGCACTGCGGGAGATCAGGAGGTACCAGAAGTCGGTCGACTTTCTCATC
CCGTTTGCACCATTTGTCCGTCTGATCAAGGAGGTCACCGACTTCTTCTGTCCTGAAATCAGCCGC
TGGACTCCCCAAGCGCTCGTCGCGATTCAAGAGGCTGCAGAGTATCACCTCGTCGACGTATTTGAA
AGGGCAAATCACTGTGCCATCCATGCAAAGCGTGTTACCGTCATGCAAAAGGACATACAGCTCGCG
AGGCGTATCGGTGGGAGGAGGCTTTGG
>L.perenne
ATGGCTCGCACGAAGCACCCAGCTGCGAGGAACTCCAGGCCGCAGCCCAAAAAGCAGCTCCAGTTC
GGGCGCTCCCCCGGCCTGGGGCCGCAGCAGGAGACAGGCGGCACGAGTACGTCGGAGGCACCGAGG
CGAGGTGGGCGAAGGGCGGCTGCAGCGACGACTCAAGCAGTGGCACCTGTGCAACAGAGGGTGAAG
AAGCCGCACCGATTCAAGCCAGGCACTGTCGCACTGCAGCAGATCAGGAAGTACCAGAAGTCCACC
GAGCTTCTCATCCCGTTTGCACCCTTTGTCCGTCTGGTTAAGGAGGTCACTAACTTCTGCTCCACC
AAGGTGTACCGCTGGACACCTCAAGCTCTCGCTGCGTTGCAAGAGGCTGCAGAATATATGTTGGTA
GACTTATTTGAAAGGGCAAATCTCTGCTCCATCCATGCAAAGCGTGTTACCCTCATGCAGAAGGAC
ATCCATCTTGCTAGGCGTATCGGGGGGCCAAGGTGG
>M.nutans
ATGGCTCGCACAAAGCACGTGGCCGTGAGGAAGTCGAAGCATCAGCCCAAGAAAAAGCTCCAGTTC
GAGGGCTCTCCTCGCCAGAGGCAGCAGCAGAAGCAGTCAGGTGGCGCTAGCGCCTCAGCGACTCCG
ATGAGAGGTGCGCGGAGCCCCGCGGTGGGAGCGGCTCAAGCAGGGACGCCTGGGCAGCAGCAACAT
CTGAAGAAGAAGCCATACCGCTGGCGGGCAGGCACGGTGGCACTGCGGGAGATCAGGAAGTACCAG
AAAACCACCGAGATGCTTATCCCATTTGCACCGTTTGTCCGTCTGGTTAGAGAGATCTGTAATTTC
ATTTCAAAAAAAGAAGTGTCACGCTGGACTCCTCAAGCACTTATTGCATTGCAAGAGGCTGCAGAG
TACCACTTAGTAGACTTATTTGAAAGGACAAATCTCTGTGCCATCCATGCAAAGCGTGTTACCATC
ATGCAAAAGGACATGCAACTCGCGAGGCGTATTGGGGGGCGAAGACCGTGG
>N.stricta
ATGGCTCGCACGAAGCACCCGGCCGCGAGGAAGGTCAAGGCGCAGCCCAAGAAGCAGCTCCAGTTC
GAGCGCTCCCCTGGCCGGACAGGTAGCGCGAGCGCAGCGGCGACCCCAGGTGGCGGGAGCGCATCG
GCGACTCCGCAGACAGGTGGGCGCCGCCCAGCCGCTCCGAGGGCTAAAGGGACCCCTAAGAAGCAG
GATGAGCAGAAAAAGCACCGATGGAGGCCAGGCACGGTGGCACTGCGGGAGATTAGGAAGTACCAG
AAATCCACCGAATTGCTTTTCCCCTTAGCACCCTTCGCCCGTTTGGTTAGGGAGATTACTCACTAC
AATTCAAAGACCGTGGACCGCTGGACTGCGGGAGCGCTTGCGACGATACAAGAGGCAGCAGAGTAC
CACATCATAGACCTGTTCGAAGTGGCAAATCTATGTGCTATTCATGCGAAGCGTGTTACCATCATG
CAAAAGGACATGCAGCTTGCAAGGCGTATCGGGGGGCGCAGGCATTGGGGG
>O.sativa
ATGGCTCGCACGAAGCACCCGGCGGTGAGGAAGTCGAAGGCGGAGCCCAAGAAGAAGCTCCAGTTC
GAACGCTCCCCTCGGCCGTCGAAGGCGCAGCGCGCTGGTGGCGGCACGGGTACCTCGGCGACCACG
AGGAGCGCGGCTGGAACATCGGCTTCAGGGACGCCTAGGCAGCAAACGAAGCAGAGGAAGCCACAC
CGCTTCCGTCCAGGCACAGTGGCACTGCGGGAGATCAGGAAATTTCAGAAAACCACCGAACTGCTG
ATCCCGTTTGCACCATTTTCTCGGCTGGTCAGGGAGATCACTGATTTCTATTCAAAGGATGTGTCA
CGGTGGACCCTTGAAGCTCTCCTTGCATTGCAAGAGGCAGCAGAATACCACTTAGTGGACATATTT
GAAGTGTCAAATCTCTGCGCCATCCATGCTAAGCGTGTTACCATCATGCAAAAGGACATGCAACTT
GCCAGGCGTATCGGTGGGCGGAGGCCATGG
>P.virgatumK
ATGGCTCGCACCAAGCACCCGGCCGTGAGGAAATCGAAGGAGCAGCCCAAGAAGAAGCTCCAGTTC
GGGCGCTCCCCGCACGGGAGGGCGACGCCGACAGGTGGAGCGAGCACATCGGCGACTCCGGCAAGC
```

GCTGCAGGGACCGGGGAGAGAGCGGCGGCTGGAGGTACGGCGGGGCCGCAGCAGCAGAAGGTGAAG
AAACCACACCGTTGGAAGCCAGGGACTGTAGCGCTGCGGGAGATCAGGAAGTTCCAGAAATCCACC
GAGATGCTTATCCCCTTTGCACCATTTGCCCGTCTGGTGAGGGAGATCACTGAGTTCTACTCAAGG
GGGAATGTGACACGCTGGACCCCGGAAGCCATCCTTGCAATACAAGAGGCAGCAGAATTCCACCTG
ATAGAACTGTTCGAAGTGGCAAATCTTTGTGCCATCCACGCCAAACGTGTTACCATCATGCAAAGG
GACATACAGCTTGCAAGGCGTATCGGTGGAAGGCGCTGG
>P.virgatumN
ATGGCTCGCACCAAGCACGCGGCCGTGAGGAAATCCAAGGAGCAGCCGAAGAAGAAGCTCCAGTTC
GGGCGCTCCCCGAACCGGAGGGCGACGCCGACAGGTGGAGCGAGCACATCGGCGACTCCGGCAAGG
GCTGCAGGGACCGGGGAGAGGGCGGCGGCTGGAGGTACGGCAGGGCGGCAGCAGCAGAGGGTGAAG
AAACCGCACCGTTGGAGGCCAGGGACTGTAGCGCTGCGGGAGATCAGGAAGTACCAGAAATCCACC
GAGTTGCTTATCCCCTTTGCACCATTTGCCCGTCTGGTGAGGGAGATCACTGACTTCTACTCAAGG
GGGAATGTAACACGCTGGACCCCGGAAGCTCTCCTTGCGATACAAGAGGCAGCAGAATTCCACCTG
ATAGATCTGTTCGAAGTGGCAAATCTTTGTGCCATCCACGCCAAACGTGTTACTATAATGCAAAAG
GACATGCAGCTTGCAAGGCGTATCGGTGGAAGGCATTGG
>R.distichophylla
ATGGCTCGCACGAAGCACCCGGCGGCGAGGAAGGCGAAGCCGCAGCCCAAGAAGAAGCTCCAGTTC
GAGCGCTCCCCTGCTCGCCGGGCTGCGCCGCCGTCGCAGCCAGGTGAAGCGAGTGCCTCGGCGACG
CCGACGAGAGATCGGCGGGCGTCGACCGGAGGGGCGCCAGGGCAGCAGCAGAAGCAGAGGAAAAAA
AGGCGATACCGGCCGGGCACGGTGGCGCTGAAGGAGATCAGGAAGTTCCAGAAATCCACCGAGCTG
CTCATCCCGTTTGCACCCTTCATCCGTCTGGTTAGGGAGATCACTAGCTTCATGTCACTGGAAGTG
ACACGATGGACTCCTCAAGCGCTCATTGCATTGCAAGAGGCAGCAGAGTACCACTTAGTAGACTTA
TTTGAAGTGGCAAATCTATGTTCCATCCATGCTAAGCGTGTTACTATCATGCAAAAGGACATACAA
CTCGCAAGGCGCATTGGGGGGCGAAGGCCGTGG
>S.angustifolia
ATGGCACGCACCAAGCACCAGGTCTCCAAGACCCAGCCGAAGCCTAGGAAGCGGCTGCAGTACGAG
CTCTCCCCTCGCCGCGGCAGGTCGCAGCAGCAAGATGGCGCTGGTACCTCGTCGCAGGCCGGAGGG
GCGCCGGCGCAGCAGCGGCAGCGGCAGCGGCGGAAGCAGCTGCGCCGGTTCCGGCCGGGCACGGTG
GCGCTGCGGGAGATCAGGAAGTACCAGAAGTCAACGGATCTGCTCATTCCCTTCGCGCCGTTTGTT
CGATTGGTGAAGGAGATCTCGGACTTCTACACGAGGGGGTTGGTGTCCCGGTGGACCCCTGAAGCC
CTCCTGGCGTTGCAAGAGGCTGCAGAGTACCACGTCGTGGACCTATTTGAACAAGCAAATCTGTGT
GCCATCCACGCAAAGCGTGTTACCATCATGCAAAAGGACATACAGCTTGCAAGGCGTATTGGGGGG
AGAAGGTCGTGG
>S.breviflora
ATGGCTCGCTCGAAGCACCCGGCAGTGAGGAAGTTGAAGCCGCAGGCCAAGAAGCAGCTCCAGTTC
GAGCGCTCGCCTCGCCGGAGTGCGCAGCAGCAGCAGCAGCACTCAGGTGGCGCGAGCCCCTCGGCG
ACCCCGAGGAGAGGTGCGCGGAGCCCGGCCGGAGCGGCGGCTCAAGGGGCATCTGGGCAACAGAAG
CACAAGAAGGCCCACCGATTCCGGCAAGGCACGGTGGCACTGCGAGAGATCAGGAAGTACCAGAAG
ACCACGGAGATGCTCATCCCATTTGCTCCCTTTGTCCGTCTGGTTAGGGAGCTCACTACTAATGTT
ACCATTGAAGTCAGCCGCTGGGAACCTCAAGCGCTCGTTGCATTGCAAGAGGCAGCAGAGTACCAC
TTGGTGGATGTATTTGAAAGGGCAAATCTCTGCGCAATCCATGCGAAGCGTGTTACCATCATGCAA
AAGGACATACAACTCGCGAGGCGTATCGGGGGGCAAAGACTGTGG
>S.cereale
ATGGCCCGCACCAAGCACCCGGCCGTGAGGAAGACCAAGGTGCCGCCCAAGAAGAAGCTCGGGACG
CGCCCCTCGGGCGGGACGCAGCGGCGGCAGGACACAGATGGCGCGGGCACGTCGGCGACTCCGAGG
CGAGCCGGGCGTGCGGCGGCGCCAGGGGCGGCTGAAGGGGCAACTGGGCAACCCAAGCAGAGGAAG
CCACACCGGTTCAGGCCAGGCACGGTGGCACTGCGGGAGATCAGGAAGTACCAGAAGTCGGTCGAG
TTTCTCATCCCTTTTGCACCATTTGTCCGTCTGATCAAGGAGGTCACCGACTTCTTCTGTCCTGAA
ATCAGCCGCTGGACTCCCCAAGCGCTCGTCGCAATTCAAGAGGCTGCAGAGTATCACCTCGTCGAC
GTATTTGAAAGGGCAAATCACTGTGCCATCCATGCAAAGCGTGTTACCGTCATGCAAAAGGACATA
CAGCTCGCGAGGCGTATCGGCGGCAGGAGGCTTTGG
>S.sibirica

ATGGCTCGCACGAAGCACCCGGCAGTGAGGAAGTCGAAGCCGCTGCCCAAGAAGCAGCTCCAGTTC
GAGCGCTCCCCTCGCGGCAGGGCGGCGGCGGCGGCGCAGCAGCAGGAGCAGGAGCAGTCAGATGGA
ATGCAGAGGAGAGGTGCGCGGCGCCAGGCCGGAGCAGCGGCTCAAGGGACATCTGGGCAACGGAAG
CAGAAGAAGGCACACCGATTCCGGCCAGGCACGTTGGCGCTGAGGGAGATCAGGAAGTTCCAGAAG
ACCACGCAGCTGCTCATCCCATTTGCACCGTTTGTCCGTCTGGTTAGGGAGCTCACTAGTAATATT
ACCATTGAAGTCAACCGCTGGAATCCTGAAGCGCTCATTGCATTGCAAGAGGCAGCAGAGTACCAC
TTGGTGGACTTATTTGAAAGGGCAAATCTGTGCGCCATTCATGCAAAGCGTGTTACCATTATGCAA
AAGGACATACAGCTCGCGAGGCGTATTGGGGGGCAAAGGCTGTGG
>T.urartu
ATGGCCCGCACCAAGCACCCGGCCGTCAGGAAGACCAAGGCGCCGCCCAAGAAGCAGCTCGGGCCC
CGCCCCGCGCAGCGGCGGCAGGAGACAGATGGCGCGGGCACGTCGGCGACACCGAGGCGAGCCGGG
CGGGCGGCGGCCCCAGGGGGGGCTCAAGGGGCAACTGGGCAACCCAAGCAGAGGAAGCCACACCGG
TTCAGGCCAGGCACGGTGGCACTGCGGGAGATCAGGAGGTATCAGAAGTCGGTCGACTTTCTCATC
CCGTTTGCACCATTTGTCCGTCTGATCAAGGAGGTCACCGACTTCTTCTGTCCTGAAATCAGCCGC
TGGACTCCCCAAGCGCTCGTCGCGATTCAAGAGGCTGCAGAGTATCACCTCGTCGACGTATTTGAA
AGGGCAAATCACTGTGCCATCCATGCAAAGCGTGTTACCGTCATGCAAAAGGACATACAGCTTGCA
AGGCGTATCGGCGGGAGGAGGCTTTGG
>Z.mayz
ATGGCTCGAACCAAGCACCAGGCCGTGAGGAAGACGGCGGAGAAGCCCAAGAAGAAGCTCCAGTTC
GAGCGCTCAGGTGGTGCGAGTACCTCGGCGACGCCGGAAAGGGCTGCTGGGACCGGGGGAAGAGCG
GCGTCTGGAGGTGACTCAGTTAAGAAGACGAAACCACGCCACCGCTGGCGGCCAGGGACTGTAGCG
CTGCGGGAGATCAGGAAGTACCAGAAGTCCACTGAACCGCTCATCCCCTTTGCGCCTTTCGTCCGT
GTGGTGAGGGAGTTAACCAATTTCGTAACAAACGGGAAAGTAGAGCGCTATACCGCAGAAGCCCTC
CTTGCGCTGCAAGAGGCAGCAGAATTCCACTTGATAGAACTGTTTGAAATGGCGAATCTGTGTGCC
ATCCATGCCAAGCGTGTCACAATCATGCAAAAGGACATACAACTTGCAAGGCGTATCGGAGGAAGG
CGTTGGGCA
