# Supplementary material for: The origin and evolution of a two-component system of paralogous genes encoding the centromeric histone CENH3 in cereals
Source: BMC Plant Biol. 2021 Nov 18;21:541. doi: 10.1186/s12870-021-03264-3 (PMC8603533; doi:10.1186/s12870-021-03264-3)
Supplement: Supplementary file 6 — Additional file 6 The sequences of mRNA βCENH3 assembled for this study. [file 12870_2021_3264_MOESM6_ESM.pdf]

Additional file 6. The sequences of mRNA *βCENH3* assembled for this study.

>A.sativa1
ATGGCCCGCACGAAGCACCCTGTGGCGAGGATGCTCAGGCTGGATCCCAAGGAGGCGCCGCCCCGC
TTCGAGTGCTCCCGTCCCTGGAGGCCGCCGCCGCCGCTGCGGGTGGTGCCGCCGCAGCAGGGGGAG
GAGAAGAGCAAGAATAAGAAGAAGAAAAAGAGGGCGTACCGGTTCCGGCCGGGAACGGTGGCGCTG
CGTGAGATCAGGAAGTACCAGCGTTCCACCGGGCTGCTCCTCCCCTTCGCGCCCTTCGTCCGCCTG
GTTAAGGAGATCACCGGCTCCCTTTCTAAGACTGTGAACCGCTGGACTCCTGAAGCGCTCGTCCCG
TTGCAAGCGGCTGCAGAGTATAACTTGGTGGACATGTTTGAAAGGGCAAATCTCTGCGCCATCCAT
GCCAAGCGTGTTACCCTCATGCCAGCGGACATACAGCTTGCCAGGCGTATCGGGGGGAAAAAGCAT
>A.sativa2
ATGGCCCGCACGAAGCACCCTGTGGCGAGGATGCTCAGGCTGGATCCCAAGGAGGCGCCGCCCCGC
TTCGAGCGCTCCCGTCCCTGGAGGCCGCCGCCGCCGCTGCGGGTGGTGCCGCCGCAGCAGGGGGGT
GAGAAGAAGAAGAACAAGAAGAAAAAGAGGGCGTACCGGTTCCGGCCGGGAACGGTGGCGCTGCGG
GAGATCAGGAAGTACCAGCGTTCCACCGGGCTGCTCCTCCCCTTCGCGCCCTTCGTCCGCCTGGTT
AAGGAGATCACCGGCTCCCTTTCTAAGACTGTGAACCGCTGGACTCCTGAAGCGCTCGTCCCGTTG
CAAGCGGCTGCAGAGTATAACTTGGTGGACATGTTTGAAAGGGCAAATCTCTGCGCCATCCATGCC
AAGCGTGTTACCCTCATGCCAGCGGACATACAGCTTGCCAGGCGTATCGGGGGCAAAAAGCAT
>A.speltoides
ATGGGTCGCACGAAGCACGCGGTGGCGGCGACGACGGCGACGACGGCGACGAAGAAGCGGCTCCGC
TTCGAGCTCTCCCCGCGATGGAGGCCGCCGCCGCCGATGCGCCAGGTACCGCCGGAGCCGCAGCCG
CAGCCGCAGCCGGAGAAGAAGAAGCGGGCGTACCGGTTCCGGCCGGGCACGGTGGCGCTGCGGGAG
ATCAGGAAGTACCAGAAGTCCACCGAGCCGCTCATCCCCTTCGCGCCCTTCGTCCGCCTGGTGAGT
GTTAAGGAGATCACCGACGACCTCACCAAGGGAGGGCTGAACCACTGGACTCCTCGGGCGCTCTTA
TCGTTGCTAGAGGCTGCAGAGTATCACATAGTCGATGTATTTGAAGATGCAAATCTATGTGCCATC
CATGCTAAGCGTGTTACCGTCATGCAAAAGGACATACAGCTCGCGAGGCGTATCGGGGGGAGAAGG
CTTTGG
>A.tauschii
ATGGGTCGCACGAAGCACGCGGTGGCGGCGACGGCGACGACGACGACGACGGAGACGAAGAAGCGG
CTCCGCTTCGAGCTCTCCCCGCGATGGAGGCCGCCGCCGCCGATGCGCCAGGTACCGCCGGAGCCG
CAGCCGGAGAAGAAGAAGAAGCGGGCGTACCGGTTCCGGCCGGGCACGGTGGCGCTGCGGGAGGTC
AGGAAGTACCAGAAGTCCACCGGGCCGCTCATCCCCTTCGCGCCCTTCGTCCGCCTGGTTAAGGAG
ATCACCAACGACCTCACGAAGGGCGAGCTGAACCACTGGACTCCTCAGGCGCTATTCGCGCTGCAA
GAGGCTGCAGAGTATCACATAGTCGATGTATTTGAAAAGGCAAATCTATGTGCCATCCATGCTAAG
CGTGTTACCATCATGCAAAAGGACATACAGCTCGCGAGGCGTATCGGGGGGAGAAGGCTTTGG
>aT.aestivum
ATGGGTCGCACGAAGCACGCGGTGGCGGCGACGGCGGCGACGACGGAGACGAAGAAGCGGCTCCGC
TTCGAGCTCTCCCCGCGCTGGAGGCCGCCGCCGGCGCTGCGCCAGGTACCGCCGGAGCCGCAGCCG
GAGAAGAAGAAGAAGCGGGCGTACCGGTTCCGGCCGGGCACGGTAGCGCTGCGGGAGGTCAGGAAG
TACCAGAAGTCCACCGGGCCGCTCATCCCCTTCGCGCCCTTCGTCCGCCTGGTTAAGGAGATCACC
GACGACTTGACGAAGGGCGAGATGAACCACTGGACTCCTCAGGCGCTCTTCTCGTTGCAGGAGGCT
GCAGAGTATCACATAGTCGATGTATTTGAAAAGGCAAATCTATGTGCCATCCATGCTAAGCGTGTT
ACCATCATGCAAAAGGACATACAGCTCGCGAGGCGTATCGGGGGGAGAAGGCCTTGG
>B.sylvaticum
ATGGCTCGCACGAAGCACCCGGTGTCGAGGATTCTGAGGCAGGGGGAGCAGCCCAAGAAGCGGGTT
CAGTACGAGCGCTCCCCTCGCTGGACTGCGCCGCCGCCGATGCGGCAGTACCCGCCGCCGCCGCTG
CCGAAGCGGAAGAAGGCGCGCCGGTCCCGGCCGGGCACGGCGGCGCTGCGGGAGATCAGGAAGCTC
CAGAGCTCCGCCGGGCTGCTCACCGCGTTCGCGCCCTTCGTCCGCCTGGTGAGGGAGATCACCGAC
TTCTATTCGAGCAGCGGTTCGTCGGGAGTGTCGCGCTGGACTCCTCAGGCGCTCGTTGCGTTGCAG
GAGGCAGCAGAGTACTACGTAGTTGACTTGTTCGGGGCTGCGAATCTCTTGGCCATCCATGCCAAG
CGTGTCACCATAACTCAAAAGGACATACAGCTGGCAAGGCGAATCAGCGGCCGATTTCTG
>bT.aestivum
ATGGGTCGCACGAAGCACGCGGTGGCGGCGACGACGGAGACGAAGAAGCGGCTCCGCTTCGAGCTA
TCCCCGCGATGGAGGCCGCCGCCGCCGATGCGCCAGGTACCGCCGGAGCCGCAGCCGCAGCCGGAG

AAGAAGAAGAAGCGGGCGTACCGGTTCCGGCCGGGCACGGTGGCGCTGCGGGAGATCAGGAAGTAC
CAGAAGTCCACCGAGCCGCTCATCCCCTTCGCGCCCTTCGTCCGCCTGGTTAAGGAGATCACCAAC
GACCTCACGAAGGGAGAGCTGAACCACTGGACTCCTCAGGCGCTCATCTCGTTGCAAGAGGCTGCA
GAGTATCACATAGTCGATGTATTTGAAGAGGCAAATCTATGTGCCATCCATGCTAAGCGTGTTACC
ATCATGCAAAAGGACATACAGCTTGCGAGGCGTATCGGGGGGAGATGGCTTTGG
>D.glomerata
ATGGCCCGCACGAAGCACCCCGCGGCGAGGATGGAGCCCAAGGAGGCGCGGCCCCGCTTCGAGCGC
TCCCGTCCCTGGAGGCCGCCGCCGCCGCTGCAGGTGGTGCCGCCGGAGCCAAGGGAGGAGAAGAAG
AGGAAGAAGAGGGCCCACCGATGGAGGCCGGGCACGCTGGCGCTGCAGGAGATCAGGAAGTACCAG
GGCTCCACCGGCCTGCTCCTCCCCTTCGCGCCCTTCATCCGCCTGGTCAAGATGATCACCGGCTCC
CTGAAGACCGACGTGACCCGCTGGACTCCTGAAGCGCTCGTCCCGTTGCAAGCGGCTGCAGAGTAT
CATTTGGTAGACTTATTTCAAAATGCACATCTCTGTGCCATCCATGCGAACCGTGTTACCATCATG
CAAGAGGACATACAACTTGCCAGGCGCATCGGGGGAGAAAGGCTTTGG
>dT.aestivum
ATGGGTCGCACGAAGCACGCGGTGGCGGCGACGGCGACGACGACGACGACGGAGACGAAGAAGCGG
CTCCGCTTCGAGCTCTCCCCGCGATGGAGGCCGCCGCCGCCGATGCGCCAGGTACCGCCGGAGCCG
CAGCCGGAGAAGAAGAAGAAGCGGGCGTACCGGTTCCGGCCGGGCACGGTGGCGCTGCGGGAGGTC
AGGAAGTACCAGAAGTCCACCGGGCCGCTCATCCCCTTCGCGCCCTTCGTCCGCCTGGTTAAGGAG
ATCACCAACGACCTCACGAAGGGCGAGCTGAACCACTGGACTCCTCAGGCGCTATTCGCGCTGCAA
GAGGCTGCAGAGTATCACATAGTCGATGTATTTGAAAAGGCAAATCTATGTGCCATCCATGCTAAG
CGTGTTACCATCATGCAAAAGGACATACAGCTCGCGAGGCGTATCGGGGGGAGAAGGCTTTGG
>L.perenne
ATGGCCCGCACGAAGCACACCGTGGCGAGGATGCCCAGGCTGGAGCCCAGGGAGGCGCCGCCCTGC
TTCGAGCGCTCCCGTCCCTGGAGGCCGCCGCCGCCGCTACGGATGGTGTCGCCAGAGCCTCGGCCG
GAGCCGGAGAAGAAGAAGAGGGCGCACCGGTCCCGCCCGGGCGCGGTGGCGCTGCGGGAGATCAGG
AAATACCAGAGCTTCACCGGTCTGCTCCTCCCCTTCGCGCCATTTGTGCGCCTGGTTAAGGAGATC
ACCAACTCCTTCTCGACCGATGTGAACCGCTGGACTCCTGAAGCGCTCGTCGCGCTGCAAGAGGCT
GCAGAGTATCGCTTGGTAGACTTATTTGAAAAGGCAAATATCTGCGCCATCCACGCCAAGCGAGTT
ACCATCATGCAAAAGGACATACATCTTGCCAGGCGCATCGGGGGGCAAAGGCATTGG
>S.breviflora
ATGGCCCGCACGAAGCGTCCGGCGGCGAGGATGTCGAGGCCGGAGCCCAAGAAGCGGCTCCAGCTC
GAGCGCTCCCCTCGCTGGAGGGCGCCGCCGCCGCTGCAGCAGCAGCCAGGGACGACACCTGCATGG
CAGCCGAAGCAGAAGAAGGCACACCGATTCCAGCCAGGCACGGTGGCGCTGCAGGAGATTAGGAAG
TTCCAGAAAACCACCGAGCTTCTCATCCCGTTTGCACCCTTCGTCCGTCTGGTTAGGGTGATCACT
AACTTCTTTTCAAAGAGGAATGCGTCGGATGTGTTGCGCTGGAATCCTCAAGCACTCATTGCATTG
CAGGAGGCAGCAGAGTACCACATAATTGACTTGTTCGAAACTGCAAATCTCTGCACCATCCATGCT
AAGCGTGTTACCGTCATGCAAAAGGACATACAGCTAGCTTGCAAGGCGTATGAGGGGGCGGAATCC
ATGGGGATAGAGATGAACGGCATT
>S.cereale
ATGGGTCGCACGAAGCACGCGGTGGCGGCGACGGCGACGACGCCGGAGACGAAGAAGCGGCTCCGC
TTCGAGCTCTCCCCGCGCTGGAGGCCGCCGCCGCCTCTATGCCAGGTACCGCCGCAGCCGCAGCCG
GAGAAGAAGAAGAAGCGGGCGTACCGGTTCCGGCCGGGCACGGTGGCGCTGCGGGAGATCAGGAAG
TACCAGAAGTCCACCGAGCCGCTCATCCCCTTCGCGCCCTTCGTCCGCCTGGTTAAGGAGATCACC
ACCGACCTCACCAAGGGAGAGATAAACCACTGGACGCCTCAGGCGCTCGTCTCGCTGCAAGAGGCT
GCAGAGTATCACATAGTCGATGTATTTGAAAAGGCAAATCTATGTGCCATCCATGCTAAGCGTGTT
ACCATCATGCAAAAGGACATACAGCTCGCGAGGCGTATCGGGGGGAGAAGGCTTTGG
>S.sibirica
ATGGCCCGCACGAAGCACCCGGCGGCGAGGATGTCGAGGCCGGAGCCCAAGAAGCGGCTCCAGTTC
GAGCGCTCCCCTCGCTGGCGGGCGCCGCCGCCGCTGCAGCAGCACCAGCAGACAGGGAAGCCTGCG
CGGCAGCAGAAGCAGAAGAAGGCACACCGGTTCCGGCCAGGCACGGTGGCGCTGAGGGAGATCAGG
AAGTTCCAGAAATCCTCCGAGCTGCTCATCCCGTTTGCACCGTTCGTGCGTCTGGTTCGGGAGATC
ACTGACTTCTATTCAAAGAGGAATGCGTGGGAAGTGTCGCGCTGGACTCCTCAAGCGCTCGTTGCA

TTGCAGGAGGCTGCAGAGTACCACATAGTAGACTTATTTGAAACTGCCAATCTCTTCGCCATCCAT
GCGAAGCGTGTTACTGTCATGCAAAAGGACATACAGCTCGTAAGGCGTATCAGGGGGCGGAATCCA
TGGGGA
>T.urartu
ATGGGTCGCACGAAGCACGCGGTGGCGGCGACGGCGGCGACGACGGAGACGAAGAAGCGGCTCCGC
TTCGAGCTCTCCCCGCGCTGGAGGCCGCCGCCGGCGCTGCGCCAGGTACCGCCGGAGCCGCAGCCG
GAGAAGAAGAAGAAGCGGGCGTACCGGTTCCGGCCGGGCACGGTGGCGCTGCGGGAGGTCAGGAAG
TACCAGAAGTCCACCGGGCCGCTCATCCCCTTCGCGCCCTTCGTCCGCCTGGTTAAGGAGATCACC
GACGACTTGACGAAGGGCGAGATGAACCACTGGACTCCTCAGGCGCTCTTCTCGTTGCAGGAGGCT
GCAGAGTATCACATAGTCGATGTATTTGAAAAGGCAAATCTATGTGCCATCCATGCTAAGCGTGTT
ACCATCATGCAAAAGGACATACAGCTCGCGAGGCGTATCGGGGGGAGAAGGCCTTGG
>H.brevisubulatum
ATGGGTCGCACGAAGCACGCGGTGGCGGCGACTACGGAGACGTCGACGACGAAGAAGCGGCTCCGC
TTCGAGCGCTCGCCACGCTGGAGGCCGCCGCCGCCGCTGCGCCAGGTAACGCCGGAGCCGCAGCCG
CAGCCGGAGAAGAAGAAGAAGAAGCGGGCGTACCGGTTCCGGCCGGGCACGGTGGCGCTGCGGGAG
ATCAGGAAGTACCAGAAGTCCACCGGGCTGCTCATCCCCTTCGCGCCCTTCGTCCGGCTGGTTAAG
GAGATCACCACCGACTTAACGAAGGGAGAGCTGAACCACTGGACTCCTCAGGCGCTCGTCTCGTTG
CAAGAGGCTGCAGAGTATCACATAATCAATGTATTCGAAAAGGCAAATCTATGTGCCATCCATGCT
AAGCGTGTTACCATGATGCAAAAGGACATACAGCTCGCGAGGCGTATCGGGGGGAGAAGGCTTTGG
>H.vulgare_1H
ATGGATCGCACGAAGCACACGGTGGGGGCGACGAAGAGGCGGCTCATCTTCGAGCGCTCCCCTCCC
CCAAGGTCGCCGCCGCCGCTGCAGGAGATAACGCCGGAGCCGCAGTCGCAGCCGGAGAAAAAGAAG
AAGCGGGCGTACCGGTTCCGGCCGGGTACGGTGGCGCTGCGGGAGATCAGGAAGTGCCGGAAGTCC
ACCGATCTGCTCATCCCCTTTGCGCCCTTCGTCCGCCTGGTTAGGGACATCGCCACCAACTACGCG
AAGGACGGGAAGCCGATGCCATGGACTCCTCACGCGCTCCTCGCGTTGCAAGAGGCTGCAAAGTAT
GACATGGTTGATGTATTTGAAAAGGCAATTCTGTGTCTCATTTATGCG
>H.vulgare_6H
ATGGCTCGCACGAAGAAAACGGTGGCGGCGAAGGAGAAGCGCCCCCCTTGCTCCAAGTCGGAGCCG
CAGTCGCAGCCGAAGAAGAAGGAGAAGCGGGCGTACCGGTTCCGGCCGGGCACGGTGGCGCTGCGG
GAGATCCGGAAGTACCGCAAGTCCACCAATATGCTCATCCCCTTTGCGCCCTTCGTCCGCCTGGTC
AGGGACATCGCCGACAACTTGACGCCATTGTCGAACAAGAAGGAGAGCAAGCCGACGCCATGGACT
CCTCTCGCGCTCCTCTCGTTGCAAGAGTCTGCAGAGTATCACTTGGTCGATCTATTTGGAAAGGCA
AATCTGTGTGCCATTCATTCGCACCGTGTTACCATCATGCTAAAGGACATGCAGCTTGCGAGGCGT
ATCGGGACGAGAAGCCTTTGG
>H.spontaneum_1H
ATGGGTCGCACGAAGCACACGGTGGGGGCGACGAAGAGGCGGCTCATCTTCGAGCGCTCCCCTCCC
CCAAGGTCGCCGCCGCCGCTGCAGGAGATAACGCCGGAGCCGCAGTCGCAGCCGGAGAAGAAGAAG
CGGGCGTACCGGTTCCGGCCGGGTACGGTGGCGCTGCGGGAGATCAGGAAGTGCCGGAAGTCCACC
GATCTGCTCATCCCCTTTGCGCCCTTCGTCCGCCTGGTTAGGGACATCGCCACCAACTACGCGAAG
GACGGGAAGCCGATGCCATGGACTCCTCACGCGCTCCTCGCGTTGCAAGAGGCTGCAAAGTATGAC
ATGGTTGATGTATTTGAAAAGGCAATTCTGTGTCTCATTTATGCG
>H.spontaneum_6H
ATGGCTCGCACGAAGAAAACGGTGGCGGCGAAGGAGAAGCGCCCCCCTTGCTCCAAGTCGGAGCCG
CAGTCGCAGCCGAAGAAGAAGGAGAAGCGGGCGTACCGGTTCCGGCCGGGCACGGTGGCGCTGCGG
GAGATCCGGAAGTACCGCAAGTCCACCAATATGCTCATCCCCTTTGCGCCCTTCGTCCGCCTGGTC
AGGGACATCGCCGACAACTTGACGCCATTGTCGAACAAGAAGGAGAGCAAGCCGACGCCATGGACT
CCTCTCGCGCTCCTCTCGTTGCAAGAGTCTGCAGAGTATCACTTGGTCGATCTATTTGGAAAGGCA
AATCTGTGTGCCATTCATTCGCACCGTGTTACCATCATGCTAAAGGACATGCAGCTTGCGAGGCGT
ATCGGGACGAGAAGCCTTTGG
