# Supplementary material for: The origin and evolution of a two-component system of paralogous genes encoding the centromeric histone CENH3 in cereals
Source: BMC Plant Biol. 2021 Nov 18;21:541. doi: 10.1186/s12870-021-03264-3 (PMC8603533; doi:10.1186/s12870-021-03264-3)
Supplement: Supplementary file 7 — Additional file 7. The amino acid sequences of αCENH3 assembled for this study. [file 12870_2021_3264_MOESM7_ESM.pdf]

Additional file 7. The amino acid sequences of αCENH3 assembled for this study.

>S.angustifolia
MARTKHQVSKTQPKPRKRLQYELSPRRGRSQQQDGAGTSSQAGGAPAQQRQRQRRKQLR
RFRPGTVALREIRKYQKSTDLLIPFAPFVRLVKEISDFYTRGLVSRWTPEALLALQEAAEYHV
VDLFEQANLCAIHAKRVTIMQKDIQLARRIGGRRSW
>P.virgatum_K
MARTKHPAVRKSKEQPKKKLQFGRSPHGRATPTGGASTSATPASAAGTGERAAAGGTAGP
QQQKVKKPHRWKPGTVALREIRKFQKSTEMLIPFAPFARLVREITEFYSRGNVTRWTPEAIL
AIQEAAEFHLIELFEVANLCAIHAKRVTIMQRDIQLARRIGGRRW
>P.virgatum_N
MARTKHAAVRKSKEQPKKKLQFGRSPNRRATPTGGASTSATPARAAGTGERAAAGGTAGR
QQQRVKKPHRWRPGTVALREIRKYQKSTELLIPFAPFARLVREITDFYSRGNVTRWTPEALL
AIQEAAEFHLIDLFEVANLCAIHAKRVTIMQKDMQLARRIGGRHW
>Z.mays
MARTKHQAVRKTAEKPKKKLQFERSGGASTSATPERAAGTGGRAASGGDSVKKTKPRHR
WRPGTVALREIRKYQKSTEPLIPFAPFVRVVRELTNFVTNGKVERYTAEALLALQEAAEFHLI
ELFEMANLCAIHAKRVTIMQKDIQLARRIGGRRWA
>O.sativaMARTKHPAVRKSKAEPKKKLQFERSPRPSKAQRAGGGTGTSATTRSAAGTSASGT
PRQQTKQRKPHRFRPGTVALREIRKFQKTTELLIPFAPFSRLVREITDFYSKDVSRWTLEALL
ALQEAAEYHLVDIFEVSNLCAIHAKRVTIMQKDMQLARRIGGRRPW
>R.distichophylla
MARTKHPAARKAKPQPKKKLQFERSPARRAAPPSQPGEASASATPTRDRRASTGGAPGQQQ
KQRKKRRYRPGTVALKEIRKFQKSTELLIPFAPFIRLVREITSFMSLEVTRWTPQALIALQEAA
EYHLVDLFEVANLCSIHAKRVTIMQKDIQLARRIGGRRPW
>N.stricta
MARTKHPAARKVKAQPKKQLQFERSPGRTGSASAAATPGGGSASATPQTGGRRPAAPRAK
GTPKKQDEQKKHRWRPGTVALREIRKYQKSTELLFPLAPFARLVREITHYNSKTVDRWTAG
ALATIQEAAEYHIIDLFEVANLCAIHAKRVTIMQKDMQLARRIGGRRHWG
>M.nutans
MARTKHVAVRKSKHQPKKKLQFEGSPRQRQQQKQSGGASASATPMRGARSPAVGAAQAG
TPGQQQHLKKKPYRWRAGTVALREIRKYQKTTEMLIPFAPFVRLVREICNFISKKEVSRWTP
QALIALQEAAEYHLVDLFERTNLCAIHAKRVTIMQKDMQLARRIGGRRPW
>S.sibirica
MARTKHPAVRKSKPLPKKQLQFERSPRGRAAAAAQQQEQEQSDGMQRRGARRQAGAAA
QGTSGQRKQKKAHRFRPGTLALREIRKFQKTTQLLIPFAPFVRLVRELTSNITIEVNRWNPEA
LIALQEAAEYHLVDLFERANLCAIHAKRVTIMQKDIQLARRIGGQRLW
>S.breviflora
MARSKHPAVRKLKPQAKKQLQFERSPRRSAQQQQQHSGGASPSATPRRGARSPAGAAAQG
ASGQQKHKKAHRFRQGTVALREIRKYQKTTEMLIPFAPFVRLVRELTTNVTIEVSRWEPQAL
VALQEAAEYHLVDVFERANLCAIHAKRVTIMQKDIQLARRIGGQRLW
>L.perenne
MARTKHPAARNSRPQPKKQLQFGRSPGLGPQQETGGTSTSEAPRRGGRRAAAATTQAVAPV
QQRVKKPHRFKPGTVALQQIRKYQKSTELLIPFAPFVRLVKEVTNFCSTKVYRWTPQALAA
LQEAAEYMLVDLFERANLCSIHAKRVTLMQKDIHLARRIGGPRW
>D.glomerata

MARTKHTAERSTRPLPKKQLQFARETGGPSTSAAPRRGARRPAATAAQGAPAQQKPRKTHR
FKPGTVALREIRKYQKSTELLIPFAPFVRLVRELTRNASIEVDRWTPQALIAIQQAAEYHLVD
LFGKANLCAIHAKRVTIMQKDIQLARRIGGLR
>A.sativa
MARTKHPAVRNSRPPPKKKLRFAARTAEQETGGASTSAAPRRGARRPTATPAPGAPEQQRTR
KPHRFKPGTVALREIRKYQKSTELLIPFAPFVRLVKEVTDWASPKVTRWTPQALVGLQEAAE
YMLVDLFERANLCAIHAKRVTLMQKDIHLARRIGGPRW
>B.distachyon
MARTKRPAIRKSKPQPKKQLQFERTGGASTSASATPGRRGGRTPARAAGQAAPAQQKPKKP
HRFRAGTVALREIRKYQKSPELLIPFAPFVRLIKEISNFYSPEISRWTPQALVALQEAAEYHLV
NIFEKANYCAIHAKRVTMMQKDIQLARRISGHRGY
>B.sylvaticum
MARTKRPAIRKSKPQPKKQLQFERAGGASTSASATPGRRGGRTPARAAGQGAPAQQKPKKP
HRFRAGTVALREIRKYQKSSELLIPFAPMVRLIKEISNFYSPEISRWTPQALVALQEAAEYHLV
NIFEKANYCAIHAKRVTMMQKDIQLARRISGHRGY
>B.stacei
MARTKRPAIRKSKPVPKKQLQFERAGGASTSASATPGRRGGRTPARAAGQGTPAQQKPKKP
HRFRPGTVALREIRKYQKSSELLIPLAPFVRLIKEISNFYSPEISRWTPQALLALQEAAEYHLV
NIFEKANYCAIHAKRVTMMQKDIQLARRISGHRGY
>H.brevisubulatum
MARTKHPAVRKSKVPPKKKIGSARSPGSAQRRQETDGAGTSATPRRAGRGAAAEGAPGQP
KQRKPHRFRPGTVALREIRKYQKSVEFLIPFAPFVRLVKEFSELYCPGITRWTPQALVAVQEA
AEYHLVDVFERANHCAIHAKRVTVMQKDIQLARRIGGRRLW
>H.vulgare
MARTKHPAVRKSKAPPKKKIGSASSPSAAQRRQETDGAGTSETPRRAGQGPAPAAAEGAPG
EPTKRKPHRFRPGTVALREIRKYQKSVNFLIPFAPFVRLVREITEYYCPRVKRWTPQALLAVQ
EATEYHLVDIFERAHLCAIHAKRVTVMQKDIQLARRIGGSKLW
>H.spontaneumMARTKHPAVRKSKAPPKKKIGSASSPSAAQRRQETDGAGTSETPRRAGRGP
APAAAEGAPGEPTKRKPHRFRPGTVALREIRKYQKSVNFLIPFAPFVRLVREITEYYCPRVKR
WTPQALLAVQEATEYHLVDIFERAHLCAIHAKRVTVMQKDIQLARRIGGSKLW
>S.cerealeMARTKHPAVRKTKVPPKKKLGTRPSGGTQRRQDTDGAGTSATPRRAGRAAAPG
AAEGATGQPKQRKPHRFRPGTVALREIRKYQKSVEFLIPFAPFVRLIKEVTDFFCPEISRWTP
QALVAIQEAAEYHLVDVFERANHCAIHAKRVTVMQKDIQLARRIGGRRLW
>T.urartu
MARTKHPAVRKTKAPPKKQLGPRPAQRRQETDGAGTSATPRRAGRAAAPGGAQGATGQPK
QRKPHRFRPGTVALREIRRYQKSVDFLIPFAPFVRLIKEVTDFFCPEISRWTPQALVAIQEAAE
YHLVDVFERANHCAIHAKRVTVMQKDIQLARRIGGRRLW
>A.speltoides
MARTKHPAVRKTKAPPKKQLGPRPAQRRQETDGAGTSATPVRAGRAAAPGAAEGATGQPK
QRKPHRFRPGTVALREIRKYQKSVDFLIPFAPFVRLIKEVTDFFCPEISRWTPQALVAIQEAAE
YHLVDVFERANHCAIHAKRVTVMQKDIQLARRIGGRRLW
>A.tauschii
MARTKHPAVRKTKAPPKKQLGPRPAQRRQETDGAGTSATPRRAGRAAAPGGAEGATGQPK
QRKPHRFRPGTVALREIRRYQKSVDFLIPFAPFVRLIKEVTDFFCPEISRWTPQALVAIQEAAE
YHLVDVFERANHCAIHAKRVTVMQKDIQLARRIGGRRLW
>T.aestivum_A

MARTKHPAVRKTKAPPKKQLGPRPAQRRQETDGAGTSATPRRAGRAAAPGGAQGATGQPK
QRKPHRFRPGTVALREIRRYQKSVDFLIPFAPFVRLIKEVTDFFCPEISRWTPQALVAIQEAAE
YHLVDVFERANHCAIHAKRVTVMQKDIQLARRIGGRRLW
>T.aestivum_B
MARTKHPAVRKTKALPKKQLGTRPSAGTPRRQETDGAGTSATPRRAGRAAAPGAAEGATG
QPKQRKPHRFRPGTVALREIRKYQKSVDFLIPFAPFVRLIKEVTDFFCPEISRWTPQALVAIQE
AAEYHLVDVFERANHCAIHAKRVTVMQKDIQLARRIGGRRLW
>T.aestivum_D
MARTKHPAVRKTKAPPKKQLGPRPAQRRQETDGAGTSATPRRAGRAAAPGGAEGATGQPK
QRKPHRFRPGTVALREIRRYQKSVDFLIPFAPFVRLIKEVTDFFCPEISRWTPQALVAIQEAAE
YHLVDVFERANHCAIHAKRVTVMQKDIQLARRIGGRRLW
