# Supplementary material for: The origin and evolution of a two-component system of paralogous genes encoding the centromeric histone CENH3 in cereals
Source: BMC Plant Biol. 2021 Nov 18;21:541. doi: 10.1186/s12870-021-03264-3 (PMC8603533; doi:10.1186/s12870-021-03264-3)
Supplement: Supplementary file 8 — Additional file 8. The amino acid sequences of βCENH3 assembled for this study. [file 12870_2021_3264_MOESM8_ESM.pdf]

Additional file 8. The amino acid sequences of βCENH3 assembled for this study.

>S.sibirica
MARTKHPAARMSRPEPKKRLQFERSPRWRAPPPLQQHQQTGKPARQQKQKKAHRFRPGTV
ALREIRKFQKSSELLIPFAPFVRLVREITDFYSKRNAWEVSRWTPQALVALQEAAEYHIVDLF
ETANLFAIHAKRVTVMQKDIQLVRRIRGRNPWG
>S.breviflora
MARTKRPAARMSRPEPKKRLQLERSPRWRAPPPLQQQPGTTPAWQPKQKKAHRFQPGTVA
LQEIRKFQKTTELLIPFAPFVRLVRVITNFFSKRNASDVLRWNPQALIALQEAAEYHIIDLFET
ANLCTIHAKRVTVMQKDIQLACKAYEGAESMGIEMNGI
>L.perenne
MARTKHTVARMPRLEPREAPPCFERSRPWRPPPPLRMVSPEPRPEPEKKKRAHRSRPGAVAL
REIRKYQSFTGLLLPFAPFVRLVKEITNSFSTDVNRWTPEALVALQEAAEYRLVDLFEKANIC
AIHAKRVTIMQKDIHLARRIGGQRHW
>D.glomerata
MARTKHPAARMEPKEARPRFERSRPWRPPPPLQVVPPEPREEKKRKKRAHRWRPGTLALQ
EIRKYQGSTGLLLPFAPFIRLVKMITGSLKTDVTRWTPEALVPLQAAAEYHLVDLFQNAHLC
AIHANRVTIMQEDIQLARRIGGERLW
>A.sativa_1
MARTKHPVARMLRLDPKEAPPRFECSRPWRPPPPLRVVPPQQGEEKSKNKKKKKRAYRFRP
GTVALREIRKYQRSTGLLLPFAPFVRLVKEITGSLSKTVNRWTPEALVPLQAAAEYNLVDMF
ERANLCAIHAKRVTLMPADIQLARRIGGKKH
>A.sativa_2
MARTKHPVARMLRLDPKEAPPRFERSRPWRPPPPLRVVPPQQGGEKKKNKKKKRAYRFRP
GTVALREIRKYQRSTGLLLPFAPFVRLVKEITGSLSKTVNRWTPEALVPLQAAAEYNLVDMF
ERANLCAIHAKRVTLMPADIQLARRIGGKKH
>B.sylvaticum
MARTKHPVSRILRQGEQPKKRVQYERSPRWTAPPPMRQYPPPPLPKRKKARRSRPGTAALR
EIRKLQSSAGLLTAFAPFVRLVREITDFYSSSGSSGVSRWTPQALVALQEAAEYYVVDLFGA
ANLLAIHAKRVTITQKDIQLARRISGRFL
>H.brevisubulatum
MGRTKHAVAATTETSTTKKRLRFERSPRWRPPPPLRQVTPEPQPQPEKKKKKRAYRFRPGTV
ALREIRKYQKSTGLLIPFAPFVRLVKEITTDLTKGELNHWTPQALVSLQEAAEYHIINVFEKA
NLCAIHAKRVTMMQKDIQLARRIGGRRLW
>H.vulgare_1H
MGRTKHTVGATKRRLIFERSPPPRSPPPRQEITPEPQSQPEKKKKRAYRFRPGTVALREIRKC
RKSTDLLIPFAPFVRLVRDIATNYAKDGKPMPWTPHALLALQEAAKYDMVDVFEKAILCLIY
A
>H.spontaneum_1H
MGRTKHTVGATKRRLIFERSPPPRSPPPLQEITPEPQSQPEKKKRAYRFRPGTVALREIRKCR
KSTDLLIPFAPFVRLVRDIATNYAKDGKPMPWTPHALLALQEAAKYDMVDVFEKAILCLIY
A
>H.vulgare_6H
MARTKKTVAAKEKRPPCSKSEPQSQPKKKEKRAYRFRPGTVALREIRKYRKSTNMLIPFAPF
VRLVRDIADNLTPLSNKKESKPTPWTPLALLSLQESAEYHLVDLFGKANLCAIHSHRVTIML
KDMQLARRIGTRSLW
>H.spontaneum_6H

MARTKKTVAAKEKRPPCSKSEPQSQPKKKEKRAYRFRPGTVALREIRKYRKSTNMLIPFAPF
VRLVRDIADNLTPLSNKKESKPTPWTPLALLSLQESAEYHLVDLFGKANLCAIHSHRVTIML
KDMQLARRIGTRSLW
>S.cereale
MGRTKHAVAATATTPETKKRLRFELSPRWRPPPPLCQVPPQPQPEKKKKRAYRFRPGTVALR
EIRKYQKSTEPLIPFAPFVRLVKEITTDLTKGEINHWTPQALVSLQEAAEYHIVDVFEKANLC
AIHAKRVTIMQKDIQLARRIGGRRLW
>T.urartu
MGRTKHAVAATAATTETKKRLRFELSPRWRPPPALRQVPPEPQPEKKKKRAYRFRPGTVALR
EVRKYQKSTGPLIPFAPFVRLVKEITDDLTKGEMNHWTPQALFSLQEAAEYHIVDVFEKAN
LCAIHAKRVTIMQKDIQLARRIGGRRPW
>A.speltoides
MGRTKHAVAATTATTATKKRLRFELSPRWRPPPPMRQVPPEPQPQPQPEKKKRAYRFRPGTV
ALREIRKYQKSTEPLIPFAPFVRLVSVKEITDDLTKGGLNHWTPRALLSLLEAAEYHIVDVFE
DANLCAIHAKRVTVMQKDIQLARRIGGRRLW
>A.tauschii
MGRTKHAVAATATTTTTETKKRLRFELSPRWRPPPPMRQVPPEPQPEKKKKRAYRFRPGTVA
LREVRKYQKSTGPLIPFAPFVRLVKEITNDLTKGELNHWTPQALFALQEAAEYHIVDVFEKA
NLCAIHAKRVTIMQKDIQLARRIGGRRLW
>T.aestivum_A
MGRTKHAVAATAATTETKKRLRFELSPRWRPPPALRQVPPEPQPEKKKKRAYRFRPGTVALR
EVRKYQKSTGPLIPFAPFVRLVKEITDDLTKGEMNHWTPQALFSLQEAAEYHIVDVFEKAN
LCAIHAKRVTIMQKDIQLARRIGGRRPW
>T.aestivum_B
MGRTKHAVAATTETKKRLRFELSPRWRPPPPMRQVPPEPQPQPEKKKKRAYRFRPGTVALR
EIRKYQKSTEPLIPFAPFVRLVKEITNDLTKGELNHWTPQALISLQEAAEYHIVDVFEEANLC
AIHAKRVTIMQKDIQLARRIGGRWLW
>T.aestivum_D
MGRTKHAVAATATTTTTETKKRLRFELSPRWRPPPPMRQVPPEPQPEKKKKRAYRFRPGTVA
LREVRKYQKSTGPLIPFAPFVRLVKEITNDLTKGELNHWTPQALFALQEAAEYHIVDVFEKA
NLCAIHAKRVTIMQKDIQLARRIGGRRLW
